# Global, regional and national epidemiological trends of multiple myeloma from 1990 to 2021: a systematic analysis of the Global Burden of Disease study 2021

**DOI:** 10.3389/fpubh.2025.1527198

**Published:** 2025-01-27

**Authors:** Linmin Zhuge, Xiaowu Lin, Ziwei Fan, Mengxian Jia, Chaowei Lin, Minyu Zhu, Honglin Teng, Guoliang Chen

**Affiliations:** ^1^Department of Gastrointestinal Surgery, The Second Affiliated Hospital of Wenzhou Medical University, Wenzhou, Zhejiang, China; ^2^Department of Orthopedics (Spine Surgery), The First Affiliated Hospital of Wenzhou Medical University, Wenzhou, Zhejiang, China

**Keywords:** Global Burden of Disease, multiple myeloma, prevalence, incidence, mortality, disability-adjusted life-years

## Abstract

**Background:**

Multiple myeloma (MM) is a growing global public health challenge. Known epidemiological data suggest that MM accounts for approximately 10% of all hematologic malignancies and remains the second most common hematologic cancer worldwide. This study utilized data from the 2021 Global Burden of Diseases (GBD) study to evaluate the prevalence, incidence, mortality, disability-adjusted life years (DALYs), and attributable risk factors of MM from 1990 to 2021, and to project future trends for the next 15 years.

**Methods:**

GBD 2021 data were analyzed to assess MM’s global burden using four key epidemiological measures: prevalence, incidence, mortality, and DALYs. Estimates are reported per 100,000 population with uncertainty intervals (UI). Temporal trends were assessed through estimated annual percentage change (EAPC) and 95% confidence intervals (CI). All analyses were conducted using R version 4.2.1.

**Results:**

From 1990 to 2021, global MM prevalence, incidence, mortality, and DALYs more than doubled, particularly among males. All Social-Demographic Index (SDI) regions showed increases in ASPR, ASIR, ASMR, and ASDR (all EAPCs >0), with the middle SDI regions exhibiting the most rapid growth. ARIMA model predictions suggest that the MM burden will continue rising over the next 15 years. The proportion of MM cases attributable to high BMI also increased globally, from 6.40% in 1990 to 7.96% in 2021. MM primarily affects older adults, with the highest incidence observed in the 70–74 age group and the highest mortality rate recorded in the same age range.

**Conclusion:**

MM presents an escalating global health challenge. Targeted preventive interventions and improvements in diagnosis, treatment, and care are critical, especially in underdeveloped regions, to address the growing global burden of MM.

## 1 Introduction

Multiple myeloma (MM) is a malignant plasma cell disease that accounts for about 10% of all hematological cancers, with associated end-organ damage and significant morbidity, including renal impairment, hypercalcemia, lytic bone lesions, and anemia (1–3). Although MM is considered as a rare disease, it is the second most common hematologic malignancy among the adult population worldwide, following non-Hodgkin lymphoma (4). The median age at diagnosis is approximately 70 years, with the disease predominantly affecting older adults. MM incidence also exhibits significant geographic variation, with higher rates reported in Western Europe and North America and relatively lower rates in Asia and sub-Saharan Africa, highlighting the influence of genetic, environmental, and healthcare factors on its distribution. Studies have estimated between 25,000 and 30,000 new cases and 12,650 deaths from MM per year in the United States (5, 6), with a global 5-year incidence of approximately 230,000 patients (7, 8). Despite advances in therapeutic strategies, the prognosis for many MM patients remains poor, with a median survival of approximately 10 years. However, in regions with limited access to these therapies, such as low- and middle-income countries, the median survival may still be considerably lower, reflecting significant disparities in treatment outcomes globally.

MM is associated with several known risk factors, including advanced age, male sex, African ancestry, and genetic predispositions. Environmental exposures, such as radiation and certain chemicals, and lifestyle factors, including high body mass index (BMI), have also been implicated as contributors to MM development [9–11]. The GBD database identifies high BMI as a significant modifiable risk factor, highlighting the potential for preventive interventions to mitigate disease burden.

MM imposes a substantial economic burden globally, with treatment costs varying significantly across regions, reflecting disparities in healthcare access and resources. Additionally, MM can also lead to a wide range of extramedullary diseases affecting the immune system, nervous system and musculoskeletal system. Secondary damage to multiple organ systems contributes substantially to the direct and indirect costs of MM treatment, further exacerbating the socioeconomic burden, particularly in regions with limited resources. The standard of care for MM depends on the patient’s overall health status. For patients younger than 70–75 years of age who are otherwise healthy, the preferred treatment for newly diagnosed MM involves a triple-drug regimen, typically including immunomodulatory drugs (IMiDs) and proteasome inhibitors (PIs) in combination with glucocorticoids, followed by autologous stem cell transplantation (ASCT) and low-dose IMiDs or PIs maintenance therapy. For patients ineligible for ASCT, standard care includes induction with novel agents and low-dose maintenance therapy (9).

The introduction of novel therapies, such as proteasome inhibitors, immunomodulatory drugs, and monoclonal antibodies (10), has significantly shifted the treatment landscape for MM (11). However, while advancements in treatment strategies have significantly improved outcomes in high-income countries, these benefits are not equally distributed, particularly in regions with limited healthcare access (7). This inequity underscores the need for globally coordinated strategies to reduce the burden of MM. As a result, disparities in treatment outcomes and survival rates across different regions remain prominent (9). Despite numerous studies on the epidemiological characteristics and economic burden of MM, updated global data remain limited. The Global Burden of Disease (GBD) 2021 study provides a comprehensive overview of the current epidemiological status of MM worldwide. Therefore, we aim to use the GBD 2021 database to describe the global epidemiology and burden of MM, predict future trends, analyze risk factors, and propose strategies for targeted prevention policies.

## 2 Methods

### 2.1 Data source and case definition

This study utilized data from GBD 2021, which provides extensive information on MM-related prevalence, incidence, mortality, and disability-adjusted life years (DALYs). Detailed descriptions of the dataset, methodologies, and statistical models are outlined in previous reports (12). MM was identified using the International Classification of Diseases (ICD-10) codes, including C90.00 (Multiple myeloma not having achieved remission), C90.01 (Multiple myeloma in remission), and C90.02 (Multiple myeloma in relapse). The analysis stratified MM cases by age, sex, and geographic location to ensure comprehensive subgroup assessments. For GBD 2021 analysis, DisMod-MR 2.1- a Bayesian meta-regression tool- was employed as the primary method to model disease metrics, ensuring internal consistency across prevalence, incidence, prevalence, and mortality estimates (13).

Prevalence and incidence were estimated through a combination of systematic reviews, population-based surveys, and health facility records. To address variability in data and adjust for heterogeneity across studies, a Bayesian Meta-regression model was applied. Mortality estimates were obtained from vital registration systems and verbal autopsy data, with correction for under-reporting and misclassification using the cause-of-death ensemble model. The years of life lost (YLLs) were calculated by multiplying the number of deaths by the standard life expectancy at the age of death, while the years lived with disability (YLDs) were calculated by multiplying disease prevalence by the corresponding disability weights, which were assigned based on population surveys and expert consultations. The disability-adjusted life years (DALYs), representing the total healthy years lost from onset to death, that is, the sum of YLL and YLD, serving as a critical measure to assess the burden of disease (14).

### 2.2 Socio-demographic index (SDI)

The Socio-Demographic Index (SDI) is a composite measure widely used in global health research, particularly in GBD studies, to assess the socio-economic development of a region or country. It integrates three key indicators: income per capita, average educational attainment in the population over 15 years of age, and total fertility rate under the age of 25. The SDI ranges from 0 to 1, with higher values reflecting greater socio-economic development. Based on SDI values, regions are categorized into five groups: low-SDI, low-middle SDI, middle SDI, high-middle SDI, and high SDI regions, facilitating comparisons of health outcomes across different levels of socio-economic development (15, 16).

### 2.3 Risk estimation for high body mass index (BMI)

High body mass index (BMI) is identified as a significant risk factor in GBD studies, defined as a BMI exceeding the theoretical minimum risk level (TMREL) of 20–25 kg/m^2^ in adults aged 20 years or older. Population-Attributable Fractions (PAF) for MM attributable to high BMI were calculated by combining population exposure estimates with relative risk metrics derived from meta-analyses. The methods for estimating population exposure to high BMI, including the use of standardized anthropometric data and survey adjustments, are detailed in prior studies (16, 17).

### 2.4 Autoregressive integrated moving average (ARIMA) model

The autoregressive integrated moving average (ARIMA) model is a statistical tool used for time series forecasting in GBD studies to model residuals after primary patterns are captured by mixed-effects models or spline interpolation. ARIMA assumes linearity, stationarity, and no autocorrelation in residuals, which were verified through diagnostic checks, including the Augmented Dickey-Fuller test and autocorrelation function analysis. While ARIMA was chosen for its suitability in handling unexplained variability, alternative forecasting models, such as exponential smoothing state-space models, were also tested. The final selection of ARIMA was based on its lower Akaike Information Criterion (AIC) values and superior predictive performance for MM-specific trends (18). Following established methodologies from prior research, we used ARIMA to project epidemiological trends for multiple myeloma (MM) across various regions over the next 15 years (19).

### 2.5 Statistical analyses

The burden of MM was quantified using ASR, including age-standardized prevalence rate (ASPR), age-standardized incidence rate (ASIR), age-standardized mortality rate (ASMR), and age-standardized DALYs rate (ASDR). Age-standardized rates are used to eliminate the impact of population age composition and ensure the comparability of research indicators. In the GBD database, these indicators are estimated using the world population age standard calculated with the following formula: $ASR=\frac{\sum_{i=1}^{A}\mathrm{aiwi}}{\sum_{i=1}^{A}wi}\times 100,000$ . In additional, we calculated estimated annual percentage change (EAPC) for ASR of prevalence, incidence, mortality, and DALYs to assess the trend change of disease burden from 1990 to 2021. The calculation methods of the ASRs and EPAC and their 95% uncertainty interval (UI) were detailed in a previous study. The calculation methods of the ASRs and EAPC and their 95% uncertainty interval (UI) were detailed in a previous study (14, 20). Trends in EAPC were interpreted by 95%CI, where the lower limit of the 95%CI greater than 0 indicated an upward trend, while the upper limit of the 95%CI less than 0 indicated a downward trend. No statistically significant difference in trend change was indicated if the 95% CI included 0 (13). Furthermore, we used percentage changes to reflect changes in prevalence, incidence, mortality, and DALYs in 2021 compared with 1990. $\mathrm{Percentage changes}=\left(2021\;\mathrm{cases}-1990\;\mathrm{cases}\right)÷1990\;\mathrm{cases}$ .

All statistical analyses (data cleaning, calculations, and plotting) were performed with R software (version 4.2.1) and final editing was performed with Adobe Illustrator software (version Adobe Illustrator CC 2022).

## 3 Results

### 3.1 Global level

In 2021, there were approximately 394.48 × 10^3^ prevalent cases of MM worldwide, with a 95%UI ranging from 355.59 × 10^3^ to 425.50 × 10^3^, representing a 218.20% increase since 1990. The ASPR in 2021 was 4.55/100,000 (95%UI 4.1/100,000–4.91/100,000), with a consistent upward trend observed from 1990 to 2021, as reflected by an EAPC of 1.24 (95%UI 1.03–1.46). Male individuals exhibited a higher prevalence [233.10 × 10^3^ (95%UI 194.94 × 10^3^–243.09 × 10^3^)] compared to females [171.38× 10^3^ (95%UI 146.60 × 10^3^–191.05 × 10^3^)]. Furthermore, the ASPR and EAPC of ASPR were both higher in males than in females (Table 1).

**Table 1:** Global prevalence, incidence, number of deaths and DALYs of multiple myeloma from 1990 to 2021.

Year	Total	Male	Female
1990			
Prevalence/1000 (95% UI)	123.97 (117.34–130.81)	62.99 (59.13–67.51)	60.98 (56.97–65.26)
Incidence/1000 (95% UI)	55.71 (52.02–59.69)	28.46 (26.27–31.12)	27.25 (25.20–29.86)
Deaths/1000 (95% UI)	47.57 (44.14–51.42)	24.08 (21.92–26.72)	23.49 (21.57–25.97)
DALYs/1000 (95% UI)	1122.52 (1041.40–1227.73)	592.33 (532.47–665.08)	530.19 (488.24–601.39)
ASPR/100,000 persons (95% UI)	3.13 (2.96–3.3)	2.86 (2.66–3.05)	3.47 (3.25–3.71)
ASIR/100,000 persons (95% UI)	1.47 (1.37–1.57)	1.7 (1.57–1.85)	1.3 (1.2–1.43)
ASMR/100,000 persons (95% UI)	1.29 (1.20–1.39)	1.5 (1.38–1.66)	1.14 (1.04–1.26)
ASDR/100,000 persons (95% UI)	28.34 (26.33–30.83)	32.57 (29.55–36.21)	24.89 (22.94–28.15)
2021			
Prevalence/1000 (95% UI)	394.48(355.59–425.50)	223.10 (194.94–243.09)	171.38 (146.60–191.05)
Incidence/1000 (95% UI)	148.76 (131.78–162.05)	82.45 (71.46–90.74)	66.30 (56.02–75.29)
Deaths/1000 (95% UI)	116.36 (103.08–128.47)	63.12 (54.44–70.18)	53.24 (44.83–60.88)
DALYs/1000 (95% UI)	2595.59 (2270.48–2889.97)	1444.32 (1219.07–1614.79)	1151.27 (940.29–1337.06)
ASPR/100,000 persons (95% UI)	4.55 (4.1–4.91)	5.53 (4.85–6.01)	3.72 (3.18–4.15)
ASIR/100,000 persons (95% UI)	1.74 (1.54–1.89)	2.12 (1.83–2.34)	1.43 (1.21–1.62)
ASMR/100,000 persons (95% UI)	1.37 (1.22–1.52)	1.67 (1.44–1.86)	1.14 (0.96–1.31)
ASDR/100,000 persons (95% UI)	30 (26.22–33.37)	35.82 (30.46–39.98)	25.04 (20.39–29.1)
1990–2021			
Prevalence (%)	218.20%	254.16%	181.04%
Incidence (%)	167.02%	189.76%	143.26%
Deaths (%)	145%	162%	127%
DALYs (%)	131%	144%	117%
EAPC of ASPR (95% CI)	1.24 (1.03–1.46)	1.57 (1.36–1.78)	0.85 (0.63–1.07)
EAPC of ASIR (95% CI)	0.48 (0.37–0.6)	0.7 (0.6–0.81)	0.2 (0.07–0.33)
EAPC of ASMR (95% CI)	0.09 (−0.01–0.18)	0.27 (0.19–0.35)	−0.14 (−0.25--0.03)
EAPC of ASDR (95% CI)	0.06 (−0.04–0.15)	0.22 (0.14–0.3)	−0.16 (−0.26--0.05)

DALYs, disability-adjusted life-years; ASPR, age-standardized prevalence rate; ASIR, age-standardized incidence rate; ASMR, age-standardized mortality rate; ASDR, age-standardized disability-adjusted life-year rate; EAPC, estimated annual percentage change; SDI, sociodemographic index; UI, uncertainty interval; CI, confidence interval.

In 2021, an estimated 148.76 × 10^3^ new cases of MM were reported worldwide, with a 95% UI ranged from 131.78 × 10^3^ to 162.05 × 10^3^, marking a 167.02% increase compared to 1990. The ASIR in 2021 was 1.74/100,000 (95%UI 1.54/100,000–1.89/100,000), up from 1.47/100,000 (95%UI 1.37/100,000–1.57/100,000) in 1990. The EAPC for ASIR from 1990 to 2021 was 0.48 (95% CI 0.37–0.6), indicating a modest increase. Among males, there were 82.45 × 10^3^ (95% UI 71.46 × 10^3^–90.74 × 10^3^) new incident cases in 2021, a 24.36% higher incidence compared to females [66.30 × 10^3^ (95%UI 56.02 × 10^3^–75.29 × 10^3^)]. Both the increased in incidence (189.76% vs. 143.26%) and EAPC for ASIR (0.7 vs. 0.2) were more pronounced in males compared to females (Table 1).

The mortality of MM increased by 145% between 1990 to 2021. In 2021, there were an estimated 116.36 × 10^3^ deaths due to MM (95%UI 103.08 × 10^3^–128.47 × 10^3^), with an ASMR of 1.37/100,000 (95%UI 1.22/100,000–1.52/100,000). The ASMR rose slightly from 1.29/100,000 (95%UI 1.20/100,000–1.39/100,000) in 1990 to 1.37/100,000 (95%UI 1.22/100,000–1.52/100,000) in 2021, with an EAPC of 0.09 (95%CI −0.01–0.15), indicating a non-significant upward trend. Mortality was higher in males [63.12 × 10^3^ (95%UI 54.44 × 10^3^–70.18 × 10^3^)] compared to females [53.24 × 10^3^ (95%UI 44.830 × 10^3^–60.88 × 10^3^)]. Similarly, the ASMR was greater in males [1.67/100,000 (95%UI 1.44/100,000–1.86/100,000)] than in females [1.14/100,000 (95%UI 0.96/100,000–1.31/100,000)] (Table 1).

Globally, DALYs due to MM increased from 1122.52 × 10^3^ (95%UI 1041.40 × 10^3^–1227.73 × 10^3^) in 1990 to 2595.59 × 10^3^ (95% UI 2270.48 × 10^3^–2889.97 × 10^3^) in 2021, reflecting a rise of 131% rise. Although the ASDR showed an increase, the EAPC was 0.06 (95% CI = −0.04 to 0.15), indicating a non-significant upward trend. Notably, the decrease in DALYs among females was more significant [−0.16 (95% CI −0.26 to −0.05)] compared to males [0.22 (95% CI 0.14 to −0.3)] (Table 1).

### 3.2 Regional level

**Figure 1:**
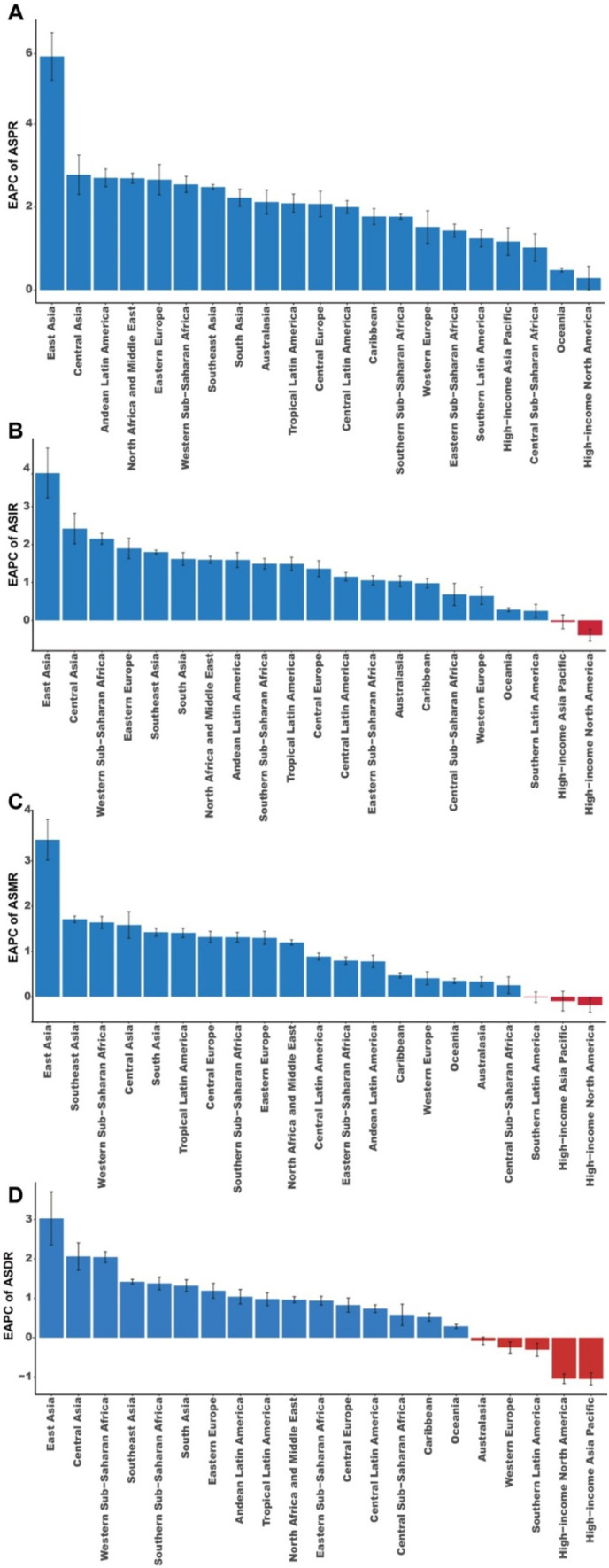
EAPCs of the ASRs for MM. **(A)** EAPCs of the ASPR for MM in 21 regions. **(B)** EAPCs of the ASIR for MM in 21 regions. **(C)** EAPCs of the ASMR for MM in 21 regions. **(D)** EAPCs of the ASDR for MM in 21 regions. ASR, age-standardized rate; ASPR, age-standardized prevalence rate; ASIR, age-standardized incidence rate; ASMR, age-standardized mortality rate; ASDR, age-standardized disability-adjusted life-year rate; EAPC, estimated annual percentage change; MM, multiple myeloma.

In 2021, the highest ASPR of MM in 2021 was observed in Australia, at 23.18/100,000 (95%UI 20.05/100,000–26.52/100,000), followed by Western Europe [15.99/100,000 (95%UI 14.79/100,00–17.02/100,000)] and the Caribbean [10.47/100,000 (95%UI 8.91/100,000–12.05/100,000)]. In contrast, the lowest ASPR values were observed in Central Africa [0.53/100,000 (95%UI 0.29/100,000–0.8/100,000)], Oceania [0.59/100,000 (95%UI 0.35/100,000–0.82/100,000)], and western sub-Saharan Africa [0.74/100,000 (95%UI 0.3/100,000–1.09/100,000)] (Supplementary Table S2). As illustrated in Figure 1A, a significant increase in ASPR was observed across all regions (EPAC >0), with the largest rise in East Asia, where the EAPC reached 5.63 (95%CI 5.06 to 6.20) (Supplementary Table S3). Additionally, in most regions, the increase in ASPR was more pronounced in males than in females (Supplementary Table S3).

Regarding incidence, the five regions with the highest incidence of MM cases in 2021 were Western Europe [41.19 × 10^3^ (95%UI 36.91 × 10^3^–44.03 × 10^3^)], High-income North America [20.90 × 10^3^ (95%UI 19.02 × 10^3^–22.01 × 10^3^)], East Asia [18.19 × 10^3^ (95%UI 11.88 × 10^3^–23.58 × 10^3^)], South Asia [15.91 × 10^3^ (95%UI 12.55 × 10^3^–21.56 × 10^3^)], and High-income Asia-Pacific [9.74 × 10^3^ (95%UI 8.16 × 10^3^–10.91 × 10^3^)] (Supplementary Table S1). The highest ASIR was observed in Western Europe [4.3/100000 (95%UI 3.91/100,000–4.57/100,000)], and the lowest ASIR was recorded in Central Africa [0.35/100,000 (0.19/100,000–0.53/100,000)] (Supplementary Table S2). Notably, ASIR was consistently higher in men than in women across all regions (Supplementary Table S2). An upward trend in ASIR was observed in most regions, except in High-income Asia Pacific and High-income North America (Figure 1B). The most notable increase in ASIR was in East Asia, with an EAPC of 3.88 (95% CI 3.23–4.54), while High-income North America showed a significant downward trend, with an EAPC of −0.39 (95% CI −0.55 to −0.24) (Supplementary Table S3 and Figure 1B). Interestingly, in Oceania, ASIR increased among women while slightly decreasing among men. Conversely, in Southern Latin America, ASIR showed a slight increase in men but decreased in women. In other regions, ASIR trends remained stable for both men and women (Supplementary Table S3).

In terms of mortality, Western Europe had the highest number of MM-ralated deaths in 2021 [95.18 × 10^3^ (95%UI 70.28 × 10^3^–119.16 × 10^3^)]. The highest ASMR was observed in Australia, at 2.89/100000 (95%UI 2.52/100000–3.23/100000), followed by High-income North America [2.81/100000 (95%UI 2.56/100000–2.96/100000)], and Western Europe [2.59/100,000 (95%UI 2.33/100,000–2.76/100,000)] (Supplementary Table S1). The largest increase in ASMR was noted in East Asia, with an EAPC of 3.46 (95%CI 3.02–3.91) (Supplementary Table S3 and Figure 1C). A rising ASMR trend was observed across most regions, except for Southern Latin America, High-income Asia Pacific, and High-income North America (Figure 1C). In regions such as Australasia, Oceania, Southern Latin America, and Western Europe, ASMR trends were similar between males and females (Supplementary Table S3).

In 2021, the regions with the highest DALYs due to MM were Western Europe [492.17 × 10^3^ (95%UI 447.36 × 10^3^–523.56 × 10^3^)], High-income North America [374.04 × 10^3^ (95%UI 348.99 × 10^3^–390.78 × 10^3^)] and East Asia [354.33 × 10^3^ (95%UI 348.99 × 10^3^–390.78 × 10^3^)] (Supplementary Table S1). High-income North America [57.28/100,000 (95%UI 53.80/100,000–59.76/100,000)], southern sub-Saharan Africa [55.55/100,000 (95%UI 36.8/100,000–67.46/100,000)], and Western Europe [53.56/100,000 (95%UI 36.8/100,000–67.46/100,000)] reported the highest ASDR in 2021 while Oceania [8.43/100,000 (95%UI 4.77/100,000–12.16/100,000)], Central Africa [8.65/100,000 (95% UI 4.58/100,000–13.35/100,000)] and Western Sub-Saharan Africa [11.1/100,000 (95%UI 4.68/100,000–16.12/100,000)] had the lowest ASDR (Supplementary Table S2). Most regions, excluding Australasia, Western Europe, Southern Latin America, high-income North America, and high-income Asia Pacific, demonstrated an upward trend in ASDR. Notably, the largest decline was observed in East Asia, with an EAPC of 3.03 (95% CI 2.35–3.70) (Figure 1D and Supplementary Table S3). ASDR in Australasia showed a negative trend in female individuals but a positive trend in male individuals. On the contrary, ASDR in Oceania showed a negative trend in male individuals and a positive trend in female individuals. The trend of ASDR in other regions was consistent in men and women (Supplementary Table S3).

### 3.3 National level

China [47.00 × 10^3^ (95%UI 29.54 × 10^3^–62.1 × 10^3^)], Germany [32.01 × 10^3^ (95%UI 27.92 × 10^3^–36.44 × 10^3^)], and the United States of America [30.70 × 10^3^ (95%UI 28.51 × 10^3^–32.09 × 10^3^)] had the highest number of MM patients in 2021 (Supplementary Table S4). New Zealand [25.35/100,000 (95%UI 21.98/100,000–29.15/100,000)], Monaco [23.46/100,000 (95%UI 11.54/100,000–39.15/100,000)], and Australia [22.75/100,000 (95%UI 19.29/100,000–26.69/100,000)] recorded the highest ASPR for 2021. Conversely, countries with the lowest ASPR included Mali [0 (95%UI 0–0)], Niger [0.08/100,000 (95%UI 0.03/100,000–0.16/100,000)], and Kiribati [0.1/100,000 (95%UI 0.0/100,0006–0.15/100,000)] (Supplementary Table S5). The percentage change in ASPR from 1990 to 2021 varied widely significantly across countries. The largest increases were observed in Georgia [6.31 (95%CI 5.56–7.07)], China [5.96 (95%CI 5.35–6.56)], and Ghana [5.18 (95% 4.98–5.38)] (Supplementary Table S5). In contrast, countries like Madagascar [−0.12 (95%CI -0.34 to 0.1)], Somalia [−0.36 (95%CI -0.41 to −0.31)], Tajikistan [−0.570 (95%CI -0.87 to −0.27)], Burundi [−0.86 (95%CI -1.07 to −0.66)], and the Northern Mariana Islands [−1.12 (95%CI -1.4 to −0.83)] showed a slight but nonsignificant downward trend in ASPR (Supplementary Table S5).

In 2021, the top four countries with the highest incidence of MM were the United States [17.69 × 10^3^ (95%UI 16.12 × 10^3^–18.61 × 10^3^)], China [17.25 × 10^3^ (95%UI 11.02 × 10^3^–22.66 × 10^3^)], Qatar [15 × 10^3^ (95%UI 9 × 10^3^–25 × 10^3^)], and India [12.59 × 10^3^ (95% UI 9.86 × 10^3^–16.60 × 10^3^)] (Supplementary Table S4). Principality of Monaco [6.86/100,000 (95%UI 3.49/100,000–10.95/100,000)], Commonwealth of the Bahamas [6.55/100,000 (95%UI 3.49/100,000–10.95/100,000)], and New Zealand [6/100,000 (95%UI 5.19/100,000–6.74/100,000)] had the highest ASIR (Supplementary Table S5). From 1990 to 2021, ASIR increased in most of the 204 countries, with Georgia [6.2 (95%CI 5.45–6.96)], Turkmenistan [6.12 (95%CI 5.52–6.72)], and Ghana [4.98 (95%CI 4.76–5.21)] had the largest increase in MM ASIR. In contrast, countries such as Argentina, Burundi, Canada, Central African Republic, Greenland, Guam, Japan, Jordan, Madagascar, Northern Mariana Islands, Republic of Nauru, Republic of San Marino, Rwanda, Singapore, Somalia, South Sudan, Sweden, Tajikistan, United States of America showed a downward trend in ASIR (EAPCs <0). The largest decreases in ASIR were observed in Burundi, Northern Mariana Islands and Tajikistan (Supplementary Table S5).

In 2021, the highest number of MM deaths were reported in China [12.98 × 10^3^ (95%UI 8.45 × 10^3^–17.11 × 10^3^)], India [11.64 × 10^3^ (95%UI 9.18 × 10^3^–15.47 × 10^3^)], and Japan [5.8 × 10^3^ (95%UI 4.80 × 10^3^–6.37 × 10^3^)] (Supplementary Table S4). The three countries with the highest ASMR of MM were the Commonwealth of the Bahamas [4.71/100,000 (95%UI 3.86/100,000–5.7/100,000)], Principality of Monaco [4.40/100,000 (95%UI 2.29/100,000–6.84/100,000)], and Grenada [3.57/100,000 (95%UI 3.10/100,000–4.04/100,000)]. Conversely, the countries with the lowest ASMR were Mali [0 (95%CI 0–0)], Republic of Palau [0.05/100,000 (95%UI 0.03/100,000–0.07/100,000)], and Niger [0.06/100,000 (95%CI 0.02/100,000–0.11/100,000)] (Supplementary Table S5). In addition, Georgia [6.18 (95% CI 5.43–6.95)], Turkmenistan [5.92 (95%CI 5.33–6.52)], and Ghana [4.93 (95% CI 4.69–5.17)] had the highest increase in the ASMR of SAH. In contrast, the Northern Mariana Islands [1.45 (95%CI −1.69 to −1.21)], Singapore [−1.44 (95%CI −1.55 to −1.33)], and Burundi [−1.2 (95%CI −1.4 to −1.01)] had the largest decrease in MM AMSR from 1990 to 2021 (Supplementary Table S5).

In 2021, the countries with the highest DALYs were China [338.36 × 10^3^ (95%UI 213.67 × 10^3^–447.64 × 10^3^)], India [299.44 × 10^3^ (95%UI 236.13 × 10^3^–397.86 × 10^3^)], and Germany [96.37 × 10^3^ (95%UI 86.55 × 10^3^–104.51 × 10^3^)] (Supplementary Table S4). Commonwealth of the Bahamas [117.52/100,000 (95%UI 94.29/100,000–145.13/100,000)], Principality of Monaco [93.34/100,000 (95%UI 47.42/100,000–150.07/100,000)], and Zimbabwe [88.68/100,000 (95%UI 48.75/100,000–126.74/100,000)] had the highest MM ASDR. The largest increase in ASDR was reported in Turkmenistan [6.04 (95% CI 5.45–6.64)], Georgia [5.98 (95% CI 5.23–6.73)]. and Ghana [4.75 (95%CI 4.52–4.98)]. In contrast, Singapore had the largest reduction in ASDR with an EPAC of −1.74 (95% CI −1.8 to −1.6) (Supplementary Table S5).

### 3.4 Burden of MM based on SDI

**Figure 2:**
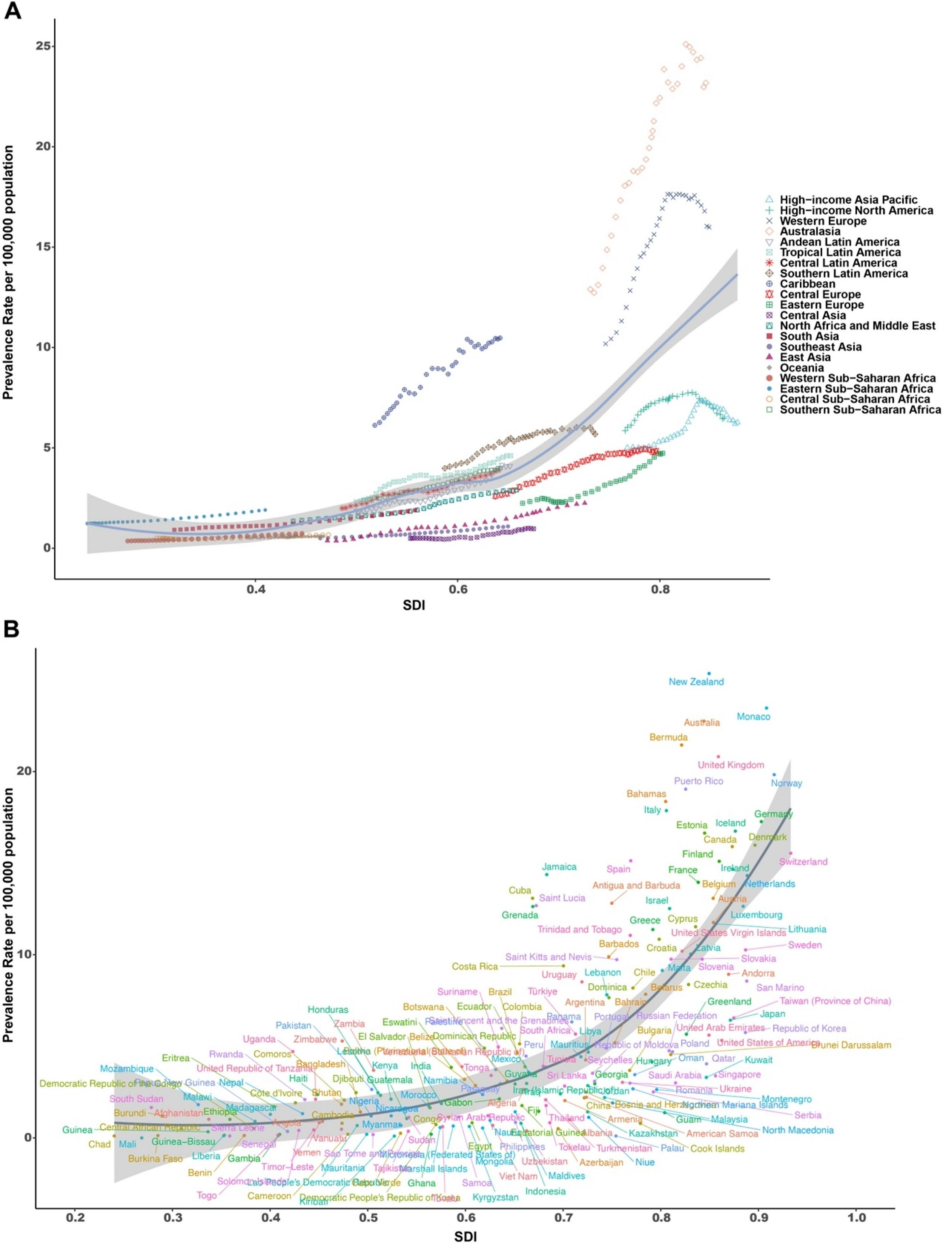
MM ASPR (based on SDI) for 21 regions and 204 countries and regions. **(A)** MM ASPR for 21 regions from 1990 to 2021 based on SDI. **(B)** MM ASPR for 204 countries and regions based on SDI (2021). ASPR, age-standardized prevalence; MM, multiple myeloma; SDI, sociodemographic index.

Regions with medium and high SDI exhibited the largest number of MM patients, as well as the highest number of new cases, deaths, and DALYs (Table 2). Across all regions, a positive correlation between the ASPR and SDI was evident, with higher SDI regions generally showing increased ASPR. However, in some high-SDI regions such as Oceania, Western Europe, high-income Asia-Pacific, and high-income North America, ASPR exhibited a negative correlation with SDI, indicating that these regions have a relatively lower prevalence despite higher SDI levels (Figure 2A). This trend was mirrored in the age-standardized incidence rate (ASIR), where regions like Western Europe, high-income Asia-Pacific, and high-income North America exhibited a similar negative correlation with SDI (Supplementary Figure S1A). The ASMR showed a positive correlation with SDI in most regions, with the exception of high-income Asia-Pacific, high-income North America, and Western Europe, where a negative correlation was observed (Supplementary Figure S2A). These regions exhibited lower MM mortality rates despite higher SDI levels, reflecting the potentially better healthcare systems or early detection methods in place. Similarly, the age-standardized DALY rate (ASDR) followed the same trend as ASMR, with a positive correlation in most regions, except for these high-SDI areas, where a negative correlation was seen (Supplementary Figure S3A). At the national level, a significant positive correlation was found between ASPR, ASIR, ASMR, ASDR, and SDI in 2021, with higher SDI countries generally exhibiting higher rates (Figure 2B and Supplementary Figures S1B, S2B, S3B). However, a few high-SDI countries, such as Monaco, Bahamas, and New Zealand, showed the highest incidence rates (ASIR) and DALYs, highlighting specific national-level health burdens (Supplementary Figures S1B, S3B).

**Table 2:** EAPC of ASPR, ASIR, ASMR, and ASDR for MM in countries with five SDI levels from 1990 to 2021.

Region	ASPR/100,000 persons (95% UI) (1990/2021)	EAPC of ASPR (95%)	ASIR/100,000 persons (95% UI) (1990/2021)	EAPC of ASIR (95% CI)	ASMR/100,000 persons (95% UI) (1990/2021)	EAPC of ASMR (95% CI)	ASDR/100,000 persons (95% UI) (1990/2021)	EAPC of ASDR (95% CI)
Low SDI	0.79 (0.44–1.11) /1.19 (0.8–1.59)	1.28 (1.09–1.48)	0.56 (0.32–0.79)/0.77 (0.51–1.02)	0.95 (0.78–1.13)	0.58 (0.33–0.81)/0.77 (0.52–1.02)	0.87 (0.7–1.03)	14.26 (7.97–20.1)/18.43 (12.25–24.91)	0.75 (0.59–0.91)
Low-middle SDI	0.8 (0.58–1.05)/1.6 (1.37–2.23)	2.23 (2.14–2.33)	0.54 (0.39–0.71)/0.92 (0.79–1.3)	1.72 (1.64–1.8)	0.55 (0.39–0.71)/0.89 (0.76–1.24)	1.53 (1.46–1.61)	13.45 (9.7–17.75)/21.51 (18.34–29.96)	1.49 (1.42–1.56)
Middle SDI	0.89 (0.78–1.12) /2.38 (1.91–2.78)	3.08 (2.92–3.24)	0.51 (0.45–0.66)/1.05 (0.84–1.23)	2.15 (1.98–2.33)	0.49 (0.43–0.63)/0.88 (0.71–1.04)	1.72 (1.54–1.91)	12.23 (10.59–15.76)/21.85 (17.5–25.56)	1.68 (1.49–1.87)
High-middle SDI	2.99 (2.82–3.19)/5.08 (4.38–5.66)	1.75 (1.59–1.91)	1.27 (1.2–1.37)/1.75 (1.52–1.95)	1.01 (0.89–1.13)	1.05 (0.99–1.14)/1.28 (1.12–1.43)	0.59 (0.48–0.71)	25.02 (23.59–27.25)/29.65 (25.53–33.21)	0.47 (0.37–0.56)
High SDI	6.96 (6.64–7.2) /9.61 (8.91–10.17)	1.14 (0.81–1.46)	2.98 (2.83–3.08)/3.16 (2.87–3.34)	0.15 (−0.02–0.33)	2.5 (2.36–2.57)/2.28 (2.05–2.41)	−0.43 (−0.54--0.32)	55.37 (53.27–56.75)/47.33 (44–49.82)	−0.64 (−0.76--0.52)

ASPR, age-standardized prevalence rate; ASIR, age-standardized incidence rate; ASMR, age-standardized mortality rate; ASDR, age-standardized disability-adjusted life-year rate; EAPC, estimated annual percentage change; MM, multiple myeloma; SDI, sociodemographic index; UI, uncertainty interval; CI, confidence interval.

### 3.5 Burden of MM based on age and sex

**Figure 3:**
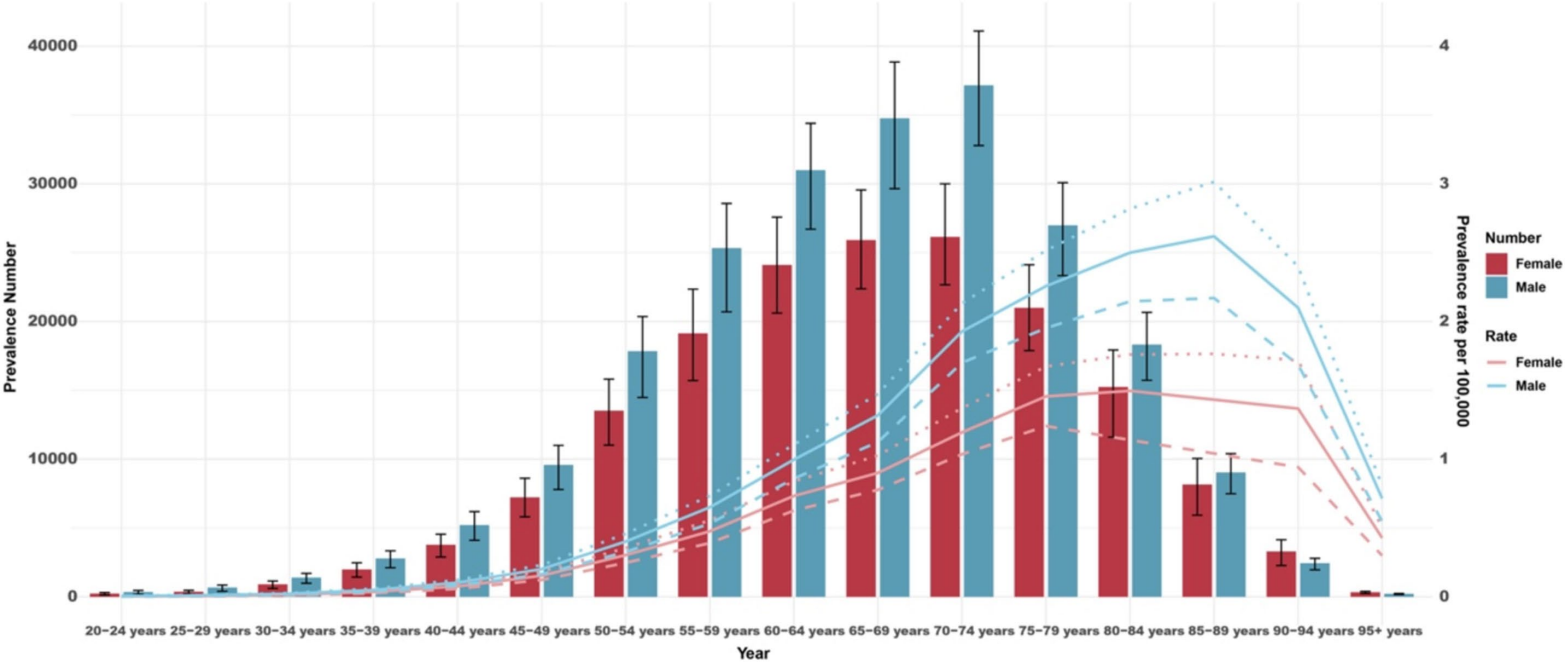
Global number of prevalent cases and prevalence estimates of multiple myeloma per 100,000 population by age and sex, 2021. Dotted and dashed lines indicate 95% upper and lower uncertainty intervals, respectively.

In 2021, the global prevalence of MM showed a clear age-dependent increase, peaking in the 70–74 group for both men and women before declining in older populations. The prevalence were higher in men, reaching its maximum in the 85–89 group, whereas for women, it peaked slightly earlier, in the 80–84 group (Figure 3). Similarly, the global incidence of MM was higher in men and increased with age, peaking in the 90–94 group. However, the number of new cases reached its maximum in the 70–74 group for both men and women, followed by decline in the older age cohorts (Supplementary Figure S4). The global mortality rates for MM also showed an age-dependent rise, increasing progressively with age for both men and women, and peaking in the 70–74 group. This trend continued into the oldest age groups, indicating a persistent and significant impact of the disease on mortality as patients age. Notably, the number of deaths aligned with this pattern, with a peak observed at 70–74 years for both sexes, before experiencing a gradual decline in subsequent age groups (Supplementary Figure S5). Additionally, the DALYs rates displayed a pattern consistent with incidence rates across different age groups and sexes (Supplementary Figure S6).

### 3.6 Predictions for MM in next 15 years

Projections from the ARIMA model suggest that the global burden of multiple myeloma (MM) will continue to rise over the next 15 years. Both prevalence and incidence rates are expected to increase for men and women, although slight declines in the age-standardized prevalence rate (ASPR) and age-standardized incidence rate (ASIR) may occur (Supplementary Figures S7, S8). Mortality and disability-adjusted life years (DALYs) attributable to MM are projected to plateau, along with stable age-standardized mortality rate (ASMR) and age-standardized DALY rate (ASDR) (Supplementary Figures S9, S10). This trend reflected the persistent challenges in managing this malignancy, particularly among men, who have significantly higher ASMR and ASDR compared to women. Moreover, the widening confidence intervals in the projections highlight the uncertainty inherent in long-term forecasting, underscoring the need for continued research and data collection to refine these estimates and better inform future healthcare strategies.

### 3.7 Attributable risk factors

**Figure 4:**
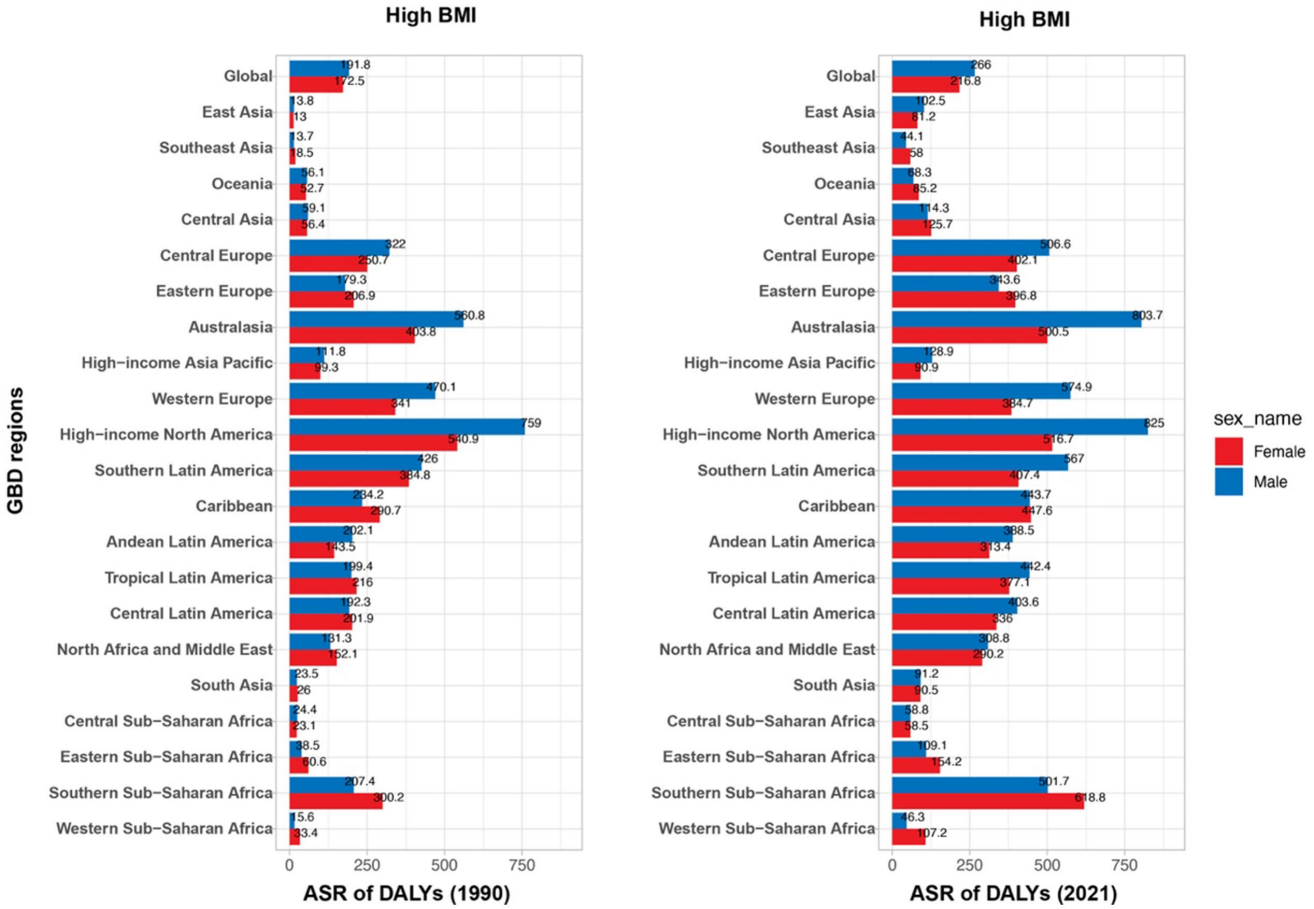
ASDR of multiple myeloma attributed to high BMI in 1990 and 2021: a comparative analysis by gender and GBD regions.

According to the GBD database, high BMI was identified as the primary risk factor contributing to MM, with the global age-standardized population attributable fraction due to high BMI increasing from 6.40% (−2.18 to 16.23) in 1990 and 7.96% (−3.16 to 19.97) in 2021 (Table 3). Over this period, there was a significant rise in the ASDR attributable to high BMI across most regions, particularly in high-income areas (Figure 4). In 2021, the global age-standardized population attributable fraction was 8.64% (−3.66 to 21.25) for females and 7.43% (−2.80 to 18.78) for males. The highest population attributable fraction was observed in high-income North America (11.52%, −5.39 to 28.00), while the lowest was in South Asia (3.61%, −1.02 to 9.35). Between 1990 and 2021, the ASDR due to high BMI significantly increased across all regions, with the most pronounced rises observed in high-income North America, Australasia, and Western Europe. This upward trend highlights the growing global burden of high BMI on MM, with men generally showing higher ASDRs than women across all regions (Figure 4). Despite some variations, the consistent increase across both sexes underscores the importance of addressing BMI as a modifiable risk factor in managing MM.

**Table 3:** Attributable age-standardized DALY rates by MM risk factors (High BMI) in 21 regions or countries in 2021.

Location	1990			2021		
	Both	Male	Female	Both	Male	Female
Global	6.40% (−2.18–16.23)	5.90% (−1.88–15.03)	6.95% (−2.43–17.79)	7.96% (−3.16–19.97)	7.43% (−2.80–18.78)	8.64% (−3.66–21.25)
East Asia	2.44% (−0.48–6.42)	2.24% (−0.39–5.93)	2.68% (−0.56–6.94)	5.61% (−1.75–14.39)	5.06% (−1.52–12.95)	6.41% (−2.15–16.60)
Southeast Asia	2.24% (−0.49–5.92)	1.79% (−0.33–4.57)	2.68% (−0.61–7.09)	4.40% (−1.32–11.33)	3.42% (−0.91–8.81)	5.44% (−1.72–14.12)
Oceania	7.16% (−2.55–18.91)	6.45% (−2.11–17.27)	8.19% (−3.19–20.79)	9.17% (−3.77–23.09)	8.52% (−3.32–21.73)	9.87% (−4.30–24.34)
Central Asia	7.41% (−2.76–19.01)	6.28% (−2.07–16.21)	8.56% (−3.36–21.97)	9.21% (−4.01–23.38)	7.97% (−3.07–20.25)	10.37% (−4.76–25.89)
Central Europe	8.54% (−3.37–21.89)	8.20% (−3.18–20.82)	8.86% (−3.55–22.67)	10.25% (−4.57–25.86)	9.79% (−4.24–24.87)	10.69% (−4.93–26.98)
Eastern Europe	8.08% (−3.11–20.73)	6.28% (−2.08–15.83)	9.59% (−4.26–24.54)	10.87% (−5.08–27.18)	9.19% (−3.94–23.58)	12.20% (−5.99–30.00)
Australasia	7.47% (−2.69–19.10)	7.15% (−2.46–18.50)	7.81% (−2.91–20.00)	10.63% (−4.81–26.70)	10.48% (−4.75–26.28)	10.81% (−4.87–26.93)
High-income Asia Pacific	3.11% (−0.70–7.97)	2.74% (−0.56–7.05)	3.46% (−0.84–8.88)	4.27% (−1.15–10.90)	4.06% (−1.08–10.49)	4.47% (−1.24–11.43)
Western Europe	6.94% (−2.42–17.74)	6.76% (−2.30–17.36)	7.09% (−2.52–18.20)	8.83% (−3.65–22.53)	8.65% (−3.50–21.88)	9.04% (−3.78–23.11)
High-income North America	8.36% (−3.34–21.20)	8.70% (−3.50–22.07)	8.99% (−3.68–22.67)	11.52% (−5.39–28.00)	11.46% (−5.35–27.98)	11.56% (−5.40–27.90)
Southern Latin America	8.19% (−3.17–20.98)	7.42% (−2.65–19.15)	9.00% (−3.85–22.79)	10.95% (−5.05–27.63)	10.37% (−4.70–26.29)	11.66% (−5.35–28.94)
Caribbean	6.14% (−2.06–15.54)	5.28% (−1.63–13.31)	7.00% (−2.45–17.64)	8.92% (−3.65–22.44)	7.99% (−3.17–20.08)	9.95% (−4.25–24.97)
Andean Latin America	6.23% (−2.05–16.05)	5.81% (1.88–15.06)	6.92% (−2.48–17.88)	9.40% (−3.96–23.61)	8.74% (−3.55–21.94)	10.28% (−4.53–25.29)
Tropical Latin America	6.68% (−2.30–17.25)	5.90% (−1.90–15.44)	7.48% (−2.74–19.13)	9.38% (−3.95–24.14)	8.94% (−3.75–22.80)	9.85% (−4.15–25.20)
Central Latin America	7.48% (−2.73–19.07)	6.62% (−2.21–17.09)	8.46% (−3.31–21.54)	10.67% (−4.86–26.82)	10.08% (−4.39–25.46)	11.34% (−5.32–28.27)
North Africa and Middle East	7.20% (−2.67–18.29)	5.90% (−1.84–14.84)	8.97% (−3.72–22.66)	11.44% (−5.51–28.30)	10.40 (−4.65–26.40)	12.84% (−6.38–31.46)
South Asia	1.58% (−0.34–4.18)	1.33% (−0.24–3.46)	1.96% (−0.46–5.21)	3.61% (−1.02–9.35)	3.08% (−0.83–8.04)	4.34% (−1.30–11.02)
Central Sub-Saharan Africa	2.55% (−0.60–6.66)	2.12% (−0.41–5.75)	3.16% (−0.86–8.16)	5.34% (−1.66–13.46)	4.74% (−1.41–12.07)	6.00% (−2.09–15.10)
Eastern Sub-Saharan Africa	2.25% (−0.50–6.04)	1.60% (−0.24–4.21)	3.01% (−0.78–7.66)	4.39% (−1.28–11.51)	3.43% (−0.92–8.98)	5.35% (−1.71–13.81)
Southern Sub-Saharan Africa	6.94% (−2.42–17.24)	4.97% (−1.49–12.58)	8.87% (−3.63–22.05)	10.16% (−4.57–24.90)	8.07% (−3.26–20.93)	12.13% (−5.82–29.90)
Western Sub-Saharan Africa	3.99% (−1.07–10.06)	3.05% (−0.72–7.78)	4.76% (−1.51–12.24)	7.04% (−2.41–17.27)	5.88% (−1.87–14.69)	7.69% (−2.83–19.03)

## 4 Discussion

Our study revealed that the global burden of MM has increased substantially over the past three decades. From 1990 to 2021, the global prevalence, incidence, mortality, and DALYs of MM have risen by approximately one to two-fold. All age-standardized rates (ASPR, ASIR, ASMR, and ASDR) showed an upward trend, with a more pronounced increase observed in males and middle-SDI regions. Although ASMR and ASDR in women did not decrease, their estimated annual percentage changes (EAPCs) were less than zero, indicating that the growth rate of the global female population may have outpaced the increase in mortality and disability rates for women with MM. Alternatively, there may have been periods of significant declines in mortality and disability rates for women during certain years within this time frame (21).

While previous studies have analyzed the epidemiological trends of hematologic neoplasms including MM, using various global databases such as the Global Cancer Observatory, WHO database, these studies face challenges due to differences in data sources, definitions, and quality (22). Earlier research has employed the GBD database to evaluate the burden of MM from 1990 to 2016 (9). Our study, based on the GBD 2021 data, provides an updated and comprehensive analysis of the global trends in MM burden. We utilized the ARIMA model to forecast the future burden of MM, and the results suggest that, over the next 15 years, MM will remain a significant global health challenge. Although prevalence and incidence rates are expected to rise, a slight decline in age-standardized rates, particularly among men, may signal improvements in early diagnosis, treatment strategies, and quality of care. However, the stabilization of mortality and DALYs underscores the ongoing challenges in managing this malignancy, especially among men, who continue to exhibit higher ASMR and ASDR compared to women.

During the period of 1990–2021, the ASPR, ASIR, ASMR, and DALYs of MM have shown an increasing trend globally, with the ASPR showing the most significant rise, reflected in its EAPC of 1.24 (95% CI 1.03–1.46). Due to the increasing incidence and mortality of MM with age, the phenomenon of the increasing age-standardized rates mentioned above might be attributed to the developing aging population which was led by the gradual development of the global economy and medical level (9, 23). Additionally, this trend could also reflect limitations in reporting and documentation, particularly in regions with underdeveloped healthcare systems, where the true incidence of MM may be underreported. By contrast, in regions like Western Europe and the United States, where healthcare systems are more advanced, improved reporting standards and greater diagnostic sensitivity have likely contributed to the observed higher incidence rates. Furthermore, the continuous refinement of international diagnostic criteria for MM, including more sensitive and specific definitions, has resulted in an increased number of patients being diagnosed with MM, further driving the observed trends (24–26). A previous study indicated that the global burden of malignant hematologic diseases, including leukemia, MM, non-Hodgkin lymphoma, and Hodgkin lymphoma, is generally higher in males (27). As in previous studies, we found that the burden of MM is higher in males than in females. However, in the 90 years and older age group, this gender disparity reversed, with more cases observed in women, possibly due to the shorter survival period of male patients compared to females (28, 29). It is noteworthy that, despite the substantial challenges in the treatment and management of MM, the global ASIR and ASDR for males have remained stable over the past three decades, whereas those for females have shown a downward trend. This trend may be closely associated with recent advancements in global healthcare quality (30).

Regionally, the most significant growth in age-standardized rates was observed in East Asia, with an EAPC exceeding 3.0. As East Asia, particularly China, continues to experience rapid economic development, it will likely face substantial challenges related to population aging and healthcare management (23, 31). Interestingly, in high-SDI regions such as high-income North America and high-income Asia-Pacific, declining trends were observed for ASIR, ASMR, and ASDR, likely reflecting advancements in treatment and healthcare quality (30, 32, 33). These findings emphasize the need for improved diagnostic, treatment, and care strategies, particularly in underdeveloped regions where healthcare access and quality remain limited. Our analysis also highlighted significant regional discrepancies in MM burden. Consequently, this underscored the necessity of establishing effective diagnosis, prevention, treatment, and care strategies for MM between both developed and underdeveloped nations. The prevalence cases, incidence cases, mortality cases, and DALYs of MM in China, the United States of America, Japan, Germany, and India rank among the highest globally. The elevated cases observed in the first four countries may be attributed to their large population bases and the increasing aging of their populations. In contrast, India’s elevated cases could be linked to inadequate healthcare management coupled with its substantial population size.

We observed significant discrepancies in the disease burden of MM across regions with varying levels of economic and social development. The age-standardized rates of MM demonstrated the highest growth in the middle SDI regions, which might be attributed to deficiencies in treatment levels, and care quality in these areas (26, 30). Furthermore, the relatively rapid economic development may contribute to an increased burden of MM. In contrast, high SDI regions reported the highest number of MM patients, newly incident cases, deaths, and DALYs. As previously mentioned, this trend is closely associated with the larger population sizes and the increasingly aging demographics in these high SDI regions (25). Additionally, this study showed that in some high SDI regions (such as Oceania, Western Europe, high-income Asia-Pacific, high-income North America), ASPR, ASIR, ASMR, and ASDR of MM exhibited a negative correlation with their SDI levels. This contrasted with the trends observed in other regions regarding age-standardized rates. The pronounced imbalance in the disease burden of MM and economic conditions across different countries might be attributed to inadequate fiscal healthcare spending, per capita income, and overall healthcare standards in countries with lower SDI compared to those with higher SDI (7). High BMI was identified as the only MM risk factor quantified in the GBD study, accounting for 7.96% of the MM burden globally. Studies have shown that high BMI increases MM risk and contributes to poorer prognosis, likely through mechanisms involving chronic low-grade inflammation, altered adipokine levels, and insulin resistance (34, 35). Although high BMI is an important risk factor, it only explains part of the MM burden. Future research should explore the contributions of other modifiable risk factors, such as environmental exposures (35, 36), occupational risks (37, 38), diet (39), and other potentially modifiable risk factors to the MM burden will be an important direction in the future.

Our ARIMA model predictions indicate that the global burden of MM will continue to rise over the next 15 years, although a slight decline in age-standardized rates is expected, particularly among men. His contrasts with the current scenario where MM predominantly affects males, suggesting potential improvements in early diagnosis, treatment strategies, and quality of care (40–42). Despite these projections, mortality and DALYs are expected to plateau, underscoring the challenges in managing MM, particularly in men. Future efforts should focus on strengthening MM surveillance and refining treatment protocols to address these ongoing challenges.

However, there were several limitations in this study. The objective of the GBD study was to provide effective, systematic, and relevant assessments of disease outcomes on a global scale (43). Although the methodologies and outcomes of GBD study were widely recognized and applied (such as sepsis, headache, atopic dermatitis, and so on), their reliability and accuracy were depended on the comparability and transparency of data regarding the targeted diseases (in the present study, MM) collected by countries and regions over the past two to three decades (43–46). Therefore, caution should be exercised when analyzing and interpreting the MM GBD results across different countries and regions in this study. Second, as previously mentioned, the diagnostic standards, assessment criteria, and therapeutic protocols for MM continually evolved over time alongside advancements in medical science (26, 40). This evolution might have implications for the comprehensiveness and accuracy of disease assessments at the initiation of data collection in relation to GBD’s research outcomes. Additionally, due to the heterogeneity observed in the symptoms of MM (typical manifestations include hypercalcemia, renal failure, anemia, and bone lesions), as well as variations in the characteristics of the cytogenetic abnormalities and the serum biomarkers, the disease burden of MM might be underestimated, particularly in lower SDI regions with the slower development of medical science (25, 26, 47). Furthermore, in recent years, the treatment protocols for MM have continuously evolved, leading to an improvement in both the disease remission rates and the median survival durations (26, 48). Looking ahead, conducting stratified analyses of ASMR and ASDR based on different treatment regimens for MM will be highly valuable.

Despite these limitations, this study provides valuable insights into the global trends and future projections for MM. Our analysis highlights the importance of preventive strategies, such as promoting weight management and lifestyle interventions, and underscores the need for targeted efforts in high-risk populations. The findings from this study could inform the development of more effective MM prevention and management strategies, particularly in regions with high disease burden and limited healthcare resources.

## 5 Conclusion

This study reveals a significant rise in the global burden of multiple myeloma (MM) from 1990 to 2021, particularly in males and middle-SDI regions. High BMI is identified as a key modifiable risk factor, contributing notably to MM burden. Although age-standardized rates are projected to stabilize, MM prevalence and incidence will continue to rise over the next 15 years, emphasizing the need for improved global prevention, early detection, and treatment strategies. Focused efforts are required in underdeveloped regions to address healthcare disparities and mitigate future challenges.

## Data Availability

The original contributions presented in the study are included in the article/Supplementary material, further inquiries can be directed to the corresponding author.
