# Supplementary material for: Global, regional and national epidemiological trends of multiple myeloma from 1990 to 2021: a systematic analysis of the Global Burden of Disease study 2021
Source: Front Public Health. 2025 Jan 27;13:1527198. doi: 10.3389/fpubh.2025.1527198 (PMC11807829; doi:10.3389/fpubh.2025.1527198)
Supplement: Supplementary file 1 [file Table_1.docx]

**TableS1. Regional prevalence, incidence, deaths, DALYs of multiple myeloma (MM) in 2021**

| Location | Prevalence/1000(95% UI) | Incidence/1000(95% UI) | Deaths/1000(95% UI) | DALYs/1000(95% UI) |
| --- | --- | --- | --- | --- |
| Andean Latin America | 2.46 (1.89-3.27) | 1.06 (0.83-1.38) | 0.88 (0.69-1.15) | 22.22 (17.27-29.04) |
| Australasia | 12.09 (10.45-13.85) | 2.99 (2.61-3.39) | 1.66 (1.44-1.86) | 32.24 (28.60-35.87) |
| Caribbean | 5.65 (4.81-6.52) | 1.71 (1.46-1.96) | 1.11 (0.957-1.26) | 26.90 (23.07-30.71) |
| Central Africa | 0.39 (0.21-0.59) | 0.23 (0.12-0.35) | 0.22 (0.12-0.34) | 6.36 (3.32-9.94) |
| Central Asia | 0.89 (0.78-1.01) | 0.44 (0.39-0.49) | 0.39 (0.34-0.43) | 11.94 (10.64-13.42) |
| Central Europe | 10.28 (9.32-11.35) | 4.95 (4.51-5.37) | 4.42 (4.03-4.79) | 95.12(86.99-103.20) |
| Central Latin America | 10.08 (8.99-11.33) | 4.14 (3.70-4.65) | 3.33(2.99-3.73) | 88.78 (79.14-99.66) |
| Central Sub-Saharan Africa | 0.41 (0.22-0.60) | 0.24 (0.13-0.35) | 0.23(0.12-0.33) | 6.65 (3.62-9.69) |
| East Asia | 49.93 (32.37-65.19) | 18.19(11.88-23.58) | 13.63(9.02-17.74) | 354.33 (228.71-464.09) |
| Eastern Europe | 16.30 (15.05-17.75) | 6.17 (5.70-6.69) | 4.65 (4.30-5.04) | 118.76 (109.04-128.80) |
| Eastern Sub-Saharan Africa | 3.49 (2.11-4.77) | 2.06 (1.25-2.82) | 1.97 (1.21-2.70) | 55.54 (33.19-77.26) |
| High-income Asia Pacific | 28.77 (24.10-33.31) | 9.74 (8.16-10.91) | 6.98 (5.83-7.78) | 118.27 (101.40-130.30) |
| High-income North America | 41.95 (38.92-44.44) | 20.90 (19.02-22.01) | 19.38(17.54-20.47) | 374.04 (348.99-390.78) |
| North Africa and Middle East | 14.22 (10.50-19.00) | 5.84 (4.39-8.01) | 4.71(3.54-6.49) | 124.88 (93.51-171.85) |
| Oceania | 0.05 (0.03-0.07) | 0.026 (0.015-0.037) | 0.024 (0.013-0.034) | 0.70 (0.38-1.01) |
| South Asia | 30.08 (23.58-40.60) | 15.91 (12.55-21.56) | 14.79 (11.66-20.03) | 383.53 (302.58-511.79) |
| Southeast Asia | 7.60 (5.85-12.20) | 3.51 (2.72-5.68) | 2.99(2.31-4.85) | 80.58 (62.89-128.42) |
| Southern Latin America | 4.85 (4.40-5.30) | 2.03 (1.88-2.19) | 1.68 (1.54-1.79) | 37.80 (35.38-40.29) |
| Southern Sub-Saharan Africa | 2.50 (1.64-3.02) | 1.35 (0.89-1.64) | 1.24(0.82-1.49) | 34.72 (23.14-42.58) |
| Tropical Latin America | 12.10 (11.32-12.80) | 5.41 (5.05-5.69) | 4.59(4.25-4.82) | 113.48 (107.33-118.28) |
| Western Europe | 140.19 (128.05-149.40) | 41.19 (36.91-44.03) | 26.88(23.76-28.88) | 492.17 (447.36-523.56) |
| Western Sub-Saharan Africa | 1.53 (0.62-2.31) | 0.90 (0.37-1.32) | 0.86(0.36-1.24) | 22.96 (9.46-33.91) |

***DALYs*** disability-adjusted life-years, ***ASPR***age-standardized prevalence rate,  ***ASIR***age-standardized incidence rate, ***ASMR*** age-standardized mortality rate, ***ASDR*** age-standardized disability-adjusted life-year rate, ***EAPC*** estimated annual percentage change, ***MM***  multiple myeloma, ***SDI***sociodemographic index, ***UI***uncertainty interval, ***CI*** confidence interval

**TableS2. ASPR, ASIR, ASMR, and ASDR for multiple myeloma (MM) in 21 regions in 2021**

| Location | ASPR/100,000 persons (95% UI) | | | ASIR/100,000 persons (95% UI) | | | ASMR/100,000 persons (95% UI) | | | ASDR /100,000 persons (95% UI) | | |
| --- | --- | --- | --- | --- | --- | --- | --- | --- | --- | --- | --- | --- |
|  | Both | Male | Female | Both | Male | Female | Both | Male | Female | Both | Male | Female |
| Andean Latin America | 4.09 (3.14-5.43) | 4.53(3.33-6.23) | 3.69(2.75-5.09) | 1.8 (1.4-2.33) | 2.11(1.58-2.85) | 1.52(1.15-2.04) | 1.51 (1.19-1.96) | 1.82(1.36-2.48) | 1.23(0.96-1.61) | 36.98 (28.79-48.2) | 44.29(32.96-60.00) | 30.25(23.24-39.33) |
| Australasia | 23.18 (20.05-26.52) | 29.22(24.87-33.56) | 17.70(14.42-21.60) | 5.48 (4.77-6.21) | 6.95(6.05-7.84) | 4.19(3.44-5.02) | 2.89 (2.52-3.23) | 3.67(3.25-4.06) | 2.23(1.87-2.62) | 60.65 (54.09-67.37) | 76.70(68.76-83.75) | 46.35(39.79-53.65) |
| Caribbean | 10.47 (8.91-12.05) | 9.53(8.02-11.22) | 11.36(9.54-13.31) | 3.17 (2.71-3.63) | 3.28(2.79-3.76) | 3.09(2.60-3.62) | 2.05 (1.77-2.33) | 2.30(1.97-2.66) | 1.84(1.57-2.16) | 49.86 (42.79-56.92) | 55.38(47.15-64.42) | 44.94(38.24-53.26) |
| Central Asia | 0.97 (0.85-1.09) | 1.00(0.88-1.14) | 0.95(0.82-1.08) | 0.5 (0.45-0.56) | 0.55(0.49-0.62) | 0.47(0.41-0.52) | 0.45 (0.41-0.51) | 0.51(0.46-0.57) | 0.42(0.37-0.47) | 13.06 (11.63-14.64) | 14.30(12.73-16.01) | 12.10(10.59-13.67) |
| Central Europe | 4.85 (4.38-5.37) | 5.44(4.86-6.05) | 4.40(3.85-5.00) | 2.21 (2.01-2.4) | 2.59(2.31-2.83) | 1.94(1.73-2.15) | 1.92 (1.75-2.08) | 2.29(2.06-2.50) | 1.66(1.50-1.84) | 43.87 (40.13-47.69) | 51.75(46.47-56.52) | 37.56(34.11-41.33) |
| Central Latin America | 3.9 (3.47-4.38) | 4.12(3.59-4.74) | 3.70(3.24-4.20) | 1.63 (1.46-1.83) | 1.84(1.60-2.09) | 1.45(1.28-1.63) | 1.33 (1.19-1.49) | 1.54(1.35-1.75) | 1.15(1.01-1.28) | 34.39 (30.68-38.61) | 39.98(34.87-45.62) | 29.53(26.07-33.22) |
| Central Sub-Saharan Africa | 0.67 (0.37-0.98) | 0.75(0.41-1.30) | 0.62(0.23-1.02) | 0.44 (0.24-0.63) | 0.51(0.28-0.89) | 0.39(0.14-0.64) | 0.44(0.23-0.63) | 0.52(0.28-0.90) | 0.38(0.13-0.63) | 10.89 (5.93-15.83) | 12.43(6.73-21.91) | 9.73(3.44-16.06) |
| East Asia | 2.25 (1.45-2.93) | 2.96(1.67-4.07) | 1.58(0.69-2.25) | 0.83 (0.54-1.07) | 1.08(0.63-1.49) | 0.61(0.27-0.85) | 0.63 (0.41-0.81) | 0.80(0.48-1.11) | 0.48(0.21-0.67) | 16.31 (10.44-21.37) | 20.32(11.72-28.08) | 12.59(5.48-17.69) |
| Eastern Europe | 4.73 (4.36-5.15) | 5.04(4.47-5.57) | 4.54(4.04-5.05) | 1.76 (1.63-1.9) | 1.91(1.70-2.11) | 1.66(1.49-1.84) | 1.31 (1.21-1.42) | 1.43(1.27-1.57) | 1.24(1.11-1.37) | 34.51 (31.74-37.42) | 37.42(33.21-41.46) | 32.48(28.97-36.30) |
| Eastern Sub-Saharan Africa | 1.9 (1.17-2.59) | 1.96(1.13-2.97) | 1.85(1.02-2.49) | 1.24 (0.77-1.68) | 1.29(0.74-1.96) | 1.19(0.66-1.58) | 1.25 (0.78-1.69) | 1.30(0.75-1.98) | 1.20(0.67-1.59) | 30.11 (18.31-41.16) | 31.75(18.01-48.29) | 28.59(15.69-38.15) |
| High-income Asia Pacific | 6.27 (5.38-7.21) | 7.30(6.13-8.65) | 5.38(4.18-6.56) | 1.93 (1.66-2.16) | 2.35(2.07-2.66) | 1.59(1.27-1.89) | 1.28 (1.09-1.41) | 1.61(1.43-1.78) | 1.03(0.82-1.19) | 25.57 (22.47-27.98) | 31.78(28.03-35.49) | 20.31(16.71-23.38) |
| High-income North America | 6.48 (6.04-6.86) | 8.74(8.12-9.32) | 4.58(4.16-4.95) | 3.1 (2.83-3.26) | 4.05(3.77-4.25) | 2.32(2.08-2.47) | 2.81 (2.56-2.96) | 3.58(3.32-3.75) | 2.20(1.95-2.35) | 57.28 (53.8-59.76) | 72.09(68.56-75.03) | 44.77(40.96-47.09) |
| North Africa and Middle East | 2.91 (2.14-3.9) | 3.23(2.21-4.33) | 2.58(1.66-3.69) | 1.3 (0.97-1.77) | 1.47(1.01-1.99) | 1.12(0.70-1.59) | 1.1 (0.83-1.51) | 1.26(0.87-1.72) | 0.94(0.61-1.30) | 26.02 (19.48-35.88) | 29.51(20.20-39.83) | 22.47(14.27-31.19) |
| Oceania | 0.59 (0.35-0.82) | 0.57(0.30-0.94) | 0.63(0.34-0.88) | 0.36 (0.21-0.5) | 0.37(0.19-0.62) | 0.35(0.19-0.49) | 0.34 (0.2-0.49) | 0.37(0.19-0.63) | 0.32(0.18-0.46) | 8.43 (4.77-12.16) | 8.25(4.12-14.15) | 8.72(4.65-12.78) |
| South Asia | 1.9 (1.49-2.58) | 2.20(1.46-2.98) | 1.59(1.03-2.35) | 1.09 (0.86-1.47) | 1.29(0.87-1.77) | 0.90(0.59-1.37) | 1.04 (0.83-1.41) | 1.24(0.85-1.73) | 0.86(0.57-1.31) | 24.96 (19.65-33.42) | 29.47(19.96-40.56) | 20.67(13.52-30.78) |
| Southeast Asia | 1.09 (0.84-1.75) | 1.16(0.81-1.97) | 1.03(0.71-1.73) | 0.53 (0.41-0.86) | 0.58(0.40-0.99) | 0.49(0.34-0.81) | 0.46 (0.36-0.75) | 0.52(0.36-0.88) | 0.42(0.29-0.71) | 11.53 (8.96-18.56) | 12.74(8.86-21.81) | 10.50(7.35-17.70) |
| Southern Latin America | 5.68 (5.15-6.21) | 6.99(6.22-7.84) | 4.57(3.93-5.24) | 2.33 (2.15-2.5) | 2.88(2.64-3.14) | 1.88(1.66-2.08) | 1.89 (1.75-2.02) | 2.33(2.16-2.51) | 1.54(1.37-1.68) | 43.97 (41.24-46.9) | 54.65(50.38-58.71) | 34.98(31.83-37.80) |
| Southern Sub-Saharan Africa | 3.99 (2.61-4.82) | 4.36(2.57-5.47) | 3.73(2.22-4.94) | 2.3 (1.51-2.77) | 2.58(1.54-3.22) | 2.11(1.26-2.77) | 2.18 (1.44-2.62) | 2.46(1.47-3.05) | 2.00(1.19-2.60) | 55.55 (36.8-67.46) | 62.08(37.24-78.30) | 50.76(30.44-66.55) |
| Tropical Latin America | 4.61 (4.31-4.88) | 4.80(4.49-5.13) | 4.46(4.09-4.86) | 2.1 (1.95-2.21) | 2.33(2.18-2.48) | 1.91(1.73-2.06) | 1.8 (1.66-1.89) | 2.06(1.92-2.18) | 1.59(1.44-1.71) | 43.43 (41-45.28) | 49.57(46.56-52.30) | 38.28(35.24-40.63) |
| Western Europe | 15.99 (14.79-17.02) | 19.86(18.25-21.40) | 12.61(11.00-13.61) | 4.3 (3.91-4.57) | 5.38(4.94-5.76) | 3.41(2.97-3.69) | 2.59 (2.33-2.76) | 3.24(2.96-3.45) | 2.09(1.81-2.26) | 53.56 (49.55-56.61) | 66.51(61.74-70.68) | 42.56(38.01-45.41) |
| Western Sub-Saharan Africa | 0.74 (0.3-1.09) | 0.51(0.25-0.70) | 0.95(0.27-1.57) | 0.48 (0.2-0.69) | 0.34(0.17-0.46) | 0.60(0.18-0.97) | 0.48 (0.2-0.69) | 0.34(0.18-0.46) | 0.60(0.18-0.96) | 11.1 (4.68-16.12) | 7.87(3.85-10.91) | 13.97(4.09-22.82) |

**TableS3. EAPC of ASPR, ASIR, ASMR, and ASDR for multiple myeloma (MM) in 21 regions from 1990 to 2021**

| Location | EAPC of ASPR (95% CI) | | | EAPC of ASIR (95% CI) | | | EAPC of ASMR (95% CI) | | | EAPC of ASDR (95% CI) | | |
| --- | --- | --- | --- | --- | --- | --- | --- | --- | --- | --- | --- | --- |
|  | Both | Male | Female | Both | Male | Female | Both | Male | Female | Both | Male | Female |
| Andean Latin America | 2.70(2.49-2.92) | 2.44(2.18-2.71) | 3.03(2.85-3.20) | 1.59(1.40-1.79) | 1.44(1.19-1.69) | 1.82(1.67-1.96) | 0.78(0.64-0.91) | 0.98(0.75-1.22) | 1.24(1.11-1.37) | 1.04(0.85-1.22) | 0.91(0.68-1.15) | 1.23(1.10-1.36) |
| Australasia | 2.12(1.83-2.41) | 2.33(2.06-2.60) | 1.76(1.43-2.08) | 1.03(0.89-1.18) | 1.13(0.99-1.28) | 0.76(0.60-0.92) | 0.33(0.23-0.44) | 0.17(0.04-0.31) | -0.09(-0.20-0.02) | -0.08(-0.18-0.02) | 0.02(-0.09-0.14) | -0.31(-0.40--0.22) |
| Caribbean | 1.77(1.58-1.96) | 1.98(1.74-2.23) | 1.60(1.42-1.78) | 0.98(0.85-1.10) | 1.12(0.96-1.28) | 0.85(0.72-0.97) | 0.47(0.42-0.53) | 0.61(0.49-0.74) | 0.29(0.17-0.40) | 0.52(0.42-0.62) | 0.71(0.58-0.84) | 0.32(0.20-0.44) |
| Central Asia | 2.78(2.30-3.25) | 2.42(1.97-2.88) | 3.10(2.58-3.63) | 2.42(2.01-2.82) | 2.10(1.71-2.50) | 2.70(2.26-3.15) | 1.58(1.29-1.88) | 1.98(1.61-2.36) | 2.54(2.12-2.96) | 2.06(1.72-2.41) | 1.75(1.40-2.09) | 2.39(2.00-2.78) |
| Central Europe | 2.07(1.76-2.38) | 2.21(1.93-2.50) | 1.95(1.62-2.28) | 1.36(1.15-1.57) | 1.46(1.27-1.66) | 1.26(1.03-1.50) | 1.32(1.19-1.44) | 1.18(1.02-1.35) | 0.98(0.78-1.18) | 0.83(0.65-1.01) | 0.90(0.73-1.07) | 0.75(0.55-0.94) |
| Central Latin America | 2.00(1.84-2.15) | 2.07(1.91-2.24) | 1.94(1.77-2.10) | 1.15(1.04-1.26) | 1.29(1.15-1.43) | 1.03(0.92-1.13) | 0.89(0.81-0.96) | 0.92(0.79-1.05) | 0.56(0.47-0.64) | 0.73(0.64-0.83) | 0.89(0.77-1.02) | 0.58(0.49-0.68) |
| Central Sub-Saharan Africa | 1.02(0.69-1.35) | 0.78(0.44-1.12) | 1.37(1.06-1.67) | 0.68(0.39-0.98) | 0.49(0.18-0.79) | 1.04(0.79-1.30) | 0.25(0.07-0.44) | 0.43(0.14-0.72) | 0.94(0.70-1.17) | 0.58(0.31-0.85) | 0.33(0.05-0.62) | 0.94(0.71-1.18) |
| East Asia | 5.63(5.06-6.20) | 6.19(5.65-6.73) | 4.76(4.14-5.38) | 3.88(3.23-4.54) | 4.39(3.76-5.03) | 3.12(2.42-3.82) | 3.46(3.02-3.91) | 3.45(2.76-4.15) | 2.34(1.60-3.09) | 3.03(2.35-3.70) | 3.50(2.85-4.16) | 2.36(1.65-3.08) |
| Eastern Europe | 2.66(2.29-3.02) | 2.58(2.15-3.01) | 2.66(2.34-2.99) | 1.90(1.63-2.17) | 1.69(1.37-2.01) | 1.97(1.73-2.21) | 1.30(1.16-1.44) | 1.24(0.97-1.51) | 1.61(1.41-1.80) | 1.19(1.00-1.38) | 1.05(0.81-1.28) | 1.26(1.08-1.43) |
| Eastern Sub-Saharan Africa | 1.43(1.27-1.59) | 1.33(1.18-1.48) | 1.55(1.38-1.71) | 1.06(0.93-1.18) | 0.94(0.82-1.06) | 1.20(1.07-1.33) | 0.80(0.72-0.88) | 0.84(0.73-0.95) | 1.10(0.99-1.22) | 0.94(0.83-1.05) | 0.84(0.73-0.94) | 1.07(0.95-1.19) |
| High-income Asia Pacific | 1.17(0.83-1.50) | 0.93(0.69-1.18) | 1.31(0.87-1.75) | -0.04(-0.22-0.15) | -0.17(-0.32--0.02) | -0.02(-0.26-0.22) | -0.10(-0.31-0.12) | -0.78(-0.91--0.65) | -0.89(-1.05--0.73) | -1.05(-1.20--0.90) | -1.01(-1.15--0.86) | -1.21(-1.38--1.04) |
| High-income North America | 0.29(0.00-0.57) | 0.49(0.24-0.75) | -0.12(-0.44-0.21) | -0.39(-0.55--0.24) | -0.29(-0.42--0.16) | -0.65(-0.84--0.46) | -0.18(-0.35--0.02) | -0.62(-0.71--0.52) | -0.86(-1.01--0.71) | -1.04(-1.16--0.92) | -0.96(-1.06--0.85) | -1.23(-1.38--1.08) |
| North Africa and Middle East | 2.69(2.57-2.81) | 2.72(2.57-2.86) | 2.66(2.56-2.76) | 1.60(1.50-1.69) | 1.62(1.50-1.73) | 1.56(1.49-1.63) | 1.20(1.14-1.25) | 1.12(1.02-1.22) | 1.06(1.00-1.12) | 0.96(0.89-1.04) | 1.00(0.90-1.10) | 0.91(0.86-0.97) |
| Oceania | 0.48(0.43-0.53) | -0.30(-0.38--0.22) | 1.39(1.30-1.48) | 0.28(0.23-0.33) | -0.41(-0.50--0.33) | 1.17(1.08-1.27) | 0.35(0.29-0.40) | -0.44(-0.53--0.35) | 1.07(0.97-1.17) | 0.29(0.23-0.34) | -0.41(-0.48--0.34) | 1.12(1.02-1.21) |
| South Asia | 2.22(2.02-2.43) | 2.35(2.15-2.54) | 2.14(1.92-2.36) | 1.62(1.45-1.79) | 1.77(1.61-1.93) | 1.52(1.34-1.70) | 1.42(1.33-1.51) | 1.58(1.43-1.72) | 1.29(1.13-1.46) | 1.32(1.17-1.47) | 1.45(1.31-1.59) | 1.23(1.07-1.40) |
| Southeast Asia | 2.48(2.42-2.54) | 2.61(2.55-2.68) | 2.35(2.30-2.41) | 1.80(1.75-1.85) | 1.97(1.92-2.02) | 1.64(1.59-1.69) | 1.71(1.64-1.78) | 1.69(1.64-1.74) | 1.31(1.26-1.37) | 1.42(1.36-1.48) | 1.61(1.55-1.67) | 1.23(1.17-1.29) |
| Southern Latin America | 1.24(1.04-1.45) | 1.55(1.38-1.72) | 0.88(0.63-1.14) | 0.25(0.07-0.42) | 0.52(0.39-0.65) | -0.05(-0.29-0.18) | -0.01(-0.13-0.10) | 0.04(-0.08-0.16) | -0.50(-0.73--0.27) | -0.31(-0.47--0.14) | -0.06(-0.19-0.07) | -0.60(-0.82--0.38) |
| Southern Sub-Saharan Africa | 1.77(1.71-1.83) | 1.64(1.48-1.80) | 1.94(1.79-2.08) | 1.49(1.35-1.63) | 1.40(1.14-1.65) | 1.64(1.56-1.72) | 1.31(1.20-1.42) | 1.28(1.00-1.56) | 1.48(1.38-1.57) | 1.38(1.22-1.54) | 1.22(0.94-1.51) | 1.59(1.48-1.69) |
| Tropical Latin America | 2.09(1.87-2.31) | 2.34(2.14-2.54) | 1.87(1.62-2.12) | 1.49(1.31-1.67) | 1.78(1.61-1.94) | 1.23(1.04-1.43) | 1.41(1.30-1.51) | 1.56(1.41-1.71) | 0.95(0.78-1.13) | 0.98(0.81-1.14) | 1.27(1.10-1.43) | 0.70(0.53-0.88) |
| Western Europe | 1.52(1.13-1.91) | 1.83(1.43-2.22) | 1.07(0.68-1.46) | 0.64(0.42-0.87) | 0.81(0.59-1.03) | 0.33(0.10-0.57) | 0.41(0.27-0.55) | 0.08(-0.04-0.19) | -0.22(-0.36--0.08) | -0.25(-0.39--0.11) | -0.15(-0.28--0.01) | -0.48(-0.63--0.32) |
| Western Sub-Saharan Africa | 2.54(2.35-2.74) | 1.90(1.80-1.99) | 2.87(2.63-3.11) | 2.15(2.00-2.29) | 1.64(1.57-1.71) | 2.43(2.25-2.62) | 1.64(1.51-1.77) | 1.57(1.50-1.64) | 2.27(2.10-2.44) | 2.04(1.91-2.18) | 1.43(1.37-1.50) | 2.38(2.20-2.57) |

***ASPR*** age-standardized prevalence rate, ***ASIR*** age-standardized incidence rate, ***ASMR*** age-standardized mortality rate, ***ASDR*** age-standardized disability-adjusted life-year rate, ***EAPC*** estimated annual percentage change, ***CI*** confidence interval

**TableS4. Prevalence, incidence, deaths, and DALYs for (MM) in 204 countries in 2021**

| **Country** | **Prevalence/1000(95% UI)** | **Incidence cases/1000 (95% UI)** | **Deaths/1000 (95% UI)** | **DALYs/1000 (95% UI)** |
| --- | --- | --- | --- | --- |
| Afghanistan | 113 (45-258) | 0.065 (0.03-0.14) | 0.06 (0.03-0.13) | 1907 (760-4322) |
| Albania | 44 (28-61) | 0.024 (0.016-0.034) | 0.02 (0.02-0.03) | 488 (319-677) |
| Algeria | 992 (669-1443) | 0.44 (0.28-0.61) | 0.38 (0.23-0.52) | 9417 (6092-13003) |
| American Samoa | 1 (1-2) | 0.001 (0-0.001) | 0.001 (0-0.001) | 14 (10-19) |
| Andorra | 14 (8-20) | 0.004 (0.003-0.006) | 0.002 (0.002-0.003) | 51 (34-73) |
| Angola | 119 (72-174) | 0.07 (0.04-0.10) | 0.07 (0.04-0.1) | 1936 (1173-2855) |
| Antigua and Barbuda | 14 (12-16) | 0.005 (0.004-0.005) | 0.003 (0.003-0.003) | 74 (68-80) |
| Argentina | 2324 (2031-2629) | 1.06(0.96-1.16) | 1.38 (1.19-1.55) | 20843 (19318-22375) |
| Armenia | 87 (70-106) | 0.042 (0.034-0.051) | 0.04 (0.03-0.04) | 996 (806-1185) |
| Australia | 9992 (8417-11687) | 2.48 (2.12-2.85) | 1.38 (1.19-1.55) | 26582 (23280-29987) |
| Austria | 2033 (1742-2377) | 0.63 (0.55-0.71) | 0.43 (0.37-0.47) | 7905 (7044-8664) |
| Azerbaijan | 61 (38-90) | 0.03 (0.02-0.05) | 0.03 (0.02-0.04) | 840 (529-1252) |
| Bahrain | 57 (33-85) | 0.02 (0.01-0.03) | 0.01 (0.008-0.02) | 423 (240-628) |
| Bangladesh | 2022 (1184-3504) | 1.10(0.67-1.89) | 1.03(0.62-1.77) | 25872 (15147-44344) |
| Barbados | 52 (38-68) | 0.02 (0.01-0.02) | 0.01 (0.01-0.01) | 250 (188-317) |
| Belarus | 1231 (953-1535) | 0.40 (0.32-0.50) | 0.26 (0.21-0.32) | 6764 (5376-8335) |
| Belgium | 2835 (2380-3292) | 0.96 (0.82-1.08) | 0.69 (0.59-0.77) | 12601 (11136-13828) |
| Belize | 9 (7-11) | 0.003 (0.003-0.004) | 0.002 (0.002-0.003) | 64 (56-73) |
| Benin | 7 (2-12) | 0.004 (0.001-0.007) | 0.004 (0.001-0.01) | 115 (34-189) |
| Bermuda | 29 (23-37) | 0.007 (0.006-0.009) | 0.004 (0.003-0.004) | 76 (64-95) |
| Bhutan | 11 (6-20) | 0.007 (0.003-0.01) | 0.006 (0.003-0.01) | 152 (76-278) |
| Bolivarian Republic of Venezuela | 1221 (872-1650) | 0.53 (0.39-0.70) | 0.44 (0.33-0.57) | 11618 (8526-15424) |
| Bosnia and Herzegovina | 154 (104-207) | 0.08 (0.06-0.11) | 0.08 (0.05-0.1) | 1706 (1145-2270) |
| Botswana | 51 (34-77) | 0.03 (0.02-0.04) | 0.03 (0.02-0.04) | 763 (511-1126) |
| Brazil | 11923 (11168-12626) | 5.33 (4.97-5.61) | 4.52 (4.19-4.75) | 111742 (105604-116276) |
| Brunei Darussalam | 17 (12-23) | 0.008 (0.006-0.01) | 0.007 (0.005-0.01) | 175 (126-232) |
| Bulgaria | 474 (364-641) | 0.21 (0.16-0.27) | 0.17 (0.13-0.22) | 3977 (3071-5131) |
| Burkina Faso | 13 (4-21) | 0.008 (0.003-0.01) | 0.008 (0.003-0.01) | 209 (69-355) |
| Burundi | 63 (29-124) | 0.04 (0.02-0.08) | 0.04 (0.02-0.07) | 1095 (499-2149) |
| Cambodia | 108 (60-214) | 0.06 (0.03-0.01) | 0.05 (0.03-0.11) | 1491 (843-3012) |
| Cameroon | 27 (8-45) | 0.02 (0.005-0.03) | 0.02 (0.01-0.03) | 430 (126-708) |
| Canada | 11252 (9727-13084) | 3.20 (2.81-3.61) | 2.01 (1.77-2.20) | 38083 (34566-41522) |
| Central African Republic | 13 (7-20) | 0.008 (0.005-0.01) | 0.008 (0.004-0.01) | 249 (136-390) |
| Chad | 7 (2-11) | 0.004 (0.001-0.007) | 0.004 (0.001-0.007) | 115 (39-192) |
| Chile | 2090 (1813-2401) | 0.78 (0.70-0.86) | 0.60 (0.54-65) | 13450 (12422-14630) |
| China | 47004 (29544-62136) | 17.25 (11.02-22.66) | 12984 (8448-17114) | 338359 (213669-447635) |
| Colombia | 2848 (2285-3502) | 1.10 (0.91-1.32) | 854 (708-1032) | 21476 (17779-26065) |
| Commonwealth of the Bahamas | 78 (59-101) | 0.03 (0.02-0.03) | 19 (15-23) | 504 (403-631) |
| Comoros | 11 (6-16) | 0.006 (0.003-0.01) | 6 (3-9) | 168 (89-250) |
| Congo | 34 (21-52) | 0.020 (0.01-0.03) | 18 (12-28) | 540 (333-817) |
| Cook Islands | 0 (0-0) | 0 (0-0) | 0 (0-0) | 2 (1-2) |
| Costa Rica | 521 (427-624) | 0.19 (0.16-0.21) | 136 (119-153) | 3531 (3093-3974) |
| Croatia | 914 (744-1145) | 0.31 (0.26-0.37) | 216 (185-254) | 4455 (3808-5280) |
| Cuba | 2538 (2112-3039) | 0.70 (0.59-0.81) | 409 (350-464) | 9746 (8423-11124) |
| Cyprus | 240 (149-337) | 0.08 (0.05-0.10) | 51 (32-69) | 1010 (648-1356) |
| Czech Republic | 1710 (1405-2060) | 0.66 (0.55-0.78) | 507 (423-594) | 10623 (8938-12257) |
| Democratic People's Republic of Korea | 249 (145-423) | 0.11 (0.07-0.19) | 97 (54-164) | 2705 (1535-4603) |
| Democratic Republic of the Congo | 208 (78-351) | 0.12 (0.05-0.21) | 118 (43-200) | 3428 (1257-5929) |
| Denmark | 1869 (1603-2140) | 0.56 (0.49-0.63) | 360 (322-402) | 6610 (5987-7300) |
| Djibouti | 18 (10-28) | 0.01 (0.006-0.02) | 9 (5-15) | 279 (153-435) |
| Dominica | 7 (4-9) | 0.003 (0.002-0.004) | 2 (1-3) | 52 (36-72) |
| Dominican Republic | 494 (329-687) | 0.20 (0.14-0.27) | 151 (106-208) | 3897 (2724-5401) |
| Ecuador | 646 (491-828) | 0.29 (0.23-0.38) | 249 (199-311) | 6196 (4887-7857) |
| Egypt | 588 (377-1091) | 0.28 (0.18-0.50) | 235 (152-427) | 6636 (4303-11948) |
| El Salvador | 99 (65-137) | 0.04 (0.03-0.06) | 34 (23-44) | 889 (591-1185) |
| Equatorial Guinea | 10 (5-16) | 0.005 (0.003-0.01) | 5 (3-8) | 139 (76-225) |
| Eritrea | 53 (28-83) | 0.03 (0.02-0.05) | 31 (16-46) | 925 (492-1436) |
| Estonia | 410 (336-499) | 0.12 (0.10-0.14) | 74 (63-84) | 1644 (1413-1885) |
| Ethiopia | 462 (236-808) | 0.28 (0.14-0.49) | 272 (138-484) | 7189 (3680-12753) |
| Federated States of Micronesia | 1 (0-1) | 0 (0-0) | 0 (0-0) | 8 (4-13) |
| Fiji | 12 (8-17) | 0.007 (0.004-0.009) | 6 (4-8) | 170 (110-238) |
| Finland | 1786 (1510-2066) | 0.54 (0.46-0.61) | 350 (304-390) | 6423 (5747-7070) |
| France | 17933 (15040-21380) | 6.01 (5.15-6.99) | 4412 (3689-5044) | 76888 (66153-87074) |
| Gabon | 25 (14-37) | 0.01 (0.008-0.02) | 13 (7-18) | 355 (195-528) |
| Georgia | 161 (134-193) | 0.08 (0.07-0.10) | 76 (65-88) | 2072 (1779-2401) |
| Germany | 32011 (27915-36438) | 8.66 (7.51-9.59) | 5140 (4504-5624) | 96367 (86551-104512) |
| Ghana | 36 (10-72) | 0.02 (0.006-0.04) | 20 (6-39) | 548 (152-1087) |
| Greece | 2420 (2079-2764) | 0.87 (0.78-0.96) | 662 (592-709) | 11851 (10824-12573) |
| Greenland | 4 (3-6) | 0.002 (0.001-0.003) | 1 (1-2) | 36 (27-52) |
| Grenada | 15 (12-18) | 0.006 (0.005-0.006) | 4 (3-5) | 100 (87-115) |
| Guam | 3 (2-4) | 0.001 (0.001-0.002) | 1 (1-1) | 28 (21-36) |
| Guatemala | 123 (99-146) | 0.06 (0.05-0.07) | 52 (44-61) | 1462 (1245-1713) |
| Guinea | 20 (11-30) | 0.01 (0.007-0.02) | 13 (7-19) | 335 (182-514) |
| Guinea-Bissau | 1 (0-2) | 0.001 (0-0.001) | 1 (0-1) | 21 (7-34) |
| Guyana | 20 (15-27) | 0.01 (0.007-0.01) | 7 (5-9) | 193 (146-251) |
| Haiti | 180 (101-358) | 0.1 (0.05-0.19) | 86 (49-162) | 2400 (1352-4599) |
| Honduras | 156 (100-233) | 0.08 (0.05-0.12) | 74 (49-109) | 1993 (1292-2976) |
| Hungary | 757 (637-895) | 0.38 (0.33-0.45) | 347 (300-396) | 7514 (6434-8692) |
| Iceland | 93 (78-110) | 0.03 (0.02-0.03) | 17 (15-19) | 331 (290-371) |
| India | 23357 (18084-30275) | 12.59 (9.86-16.60) | 11635 (9184-15470) | 299444 (236130-397864) |
| Indonesia | 2113 (1379-3872) | 1.09 (0.70-2.023) | 973 (623-1834) | 27451 (17979-50395) |
| Iraq | 800 (518-1195) | 0.34 (0.22-0.48) | 274 (180-390) | 7790 (5027-11145) |
| Ireland | 1135 (945-1331) | 0.34 (0.29-0.39) | 220 (191-247) | 4302 (3805-4831) |
| Islamic Republic of Iran | 2006 (1230-2606) | 0.77 (0.49-0.96) | 593 (385-717) | 15873 (10103-18859) |
| Israel | 1489 (1248-1729) | 0.51 (0.44-0.57) | 373 (325-411) | 7151 (6387-7821) |
| Italy | 23451 (20361-26540) | 6.67 (5.84-7.40) | 4218 (3671-4577) | 76798 (68659-82550) |
| Jamaica | 438 (325-576) | 0.15 (0.12-0.20) | 107 (83-137) | 2575 (1955-3324) |
| Japan | 22973 (18942-26585) | 7.97 (6.71-8.88) | 5807 (4800-6373) | 93334 (80942-101131) |
| Jordan | 244 (150-364) | 0.09 (0.06-0.13) | 69 (43-100) | 1941 (1202-2855) |
| Kazakhstan | 284 (231-348) | 0.13 (0.11-0.16) | 113 (95-134) | 3505 (2928-4136) |
| Kenya | 530 (340-734) | 0.30 (0.19-0.42) | 284 (183-393) | 7939 (5140-11148) |
| Kingdom of Eswatini | 24 (15-37) | 0.01 (0.01-0.02) | 13 (9-20) | 400 (255-620) |
| Kiribati | 0 (0-0) | 0 (0-0) | 0 (0-0) | 1 (1-2) |
| Kuwait | 122 (94-158) | 0.04 (0.03-0.04) | 22 (18-27) | 680 (563-807) |
| Kyrgyzstan | 36 (27-47) | 0.02 (0.01-0.02) | 15 (11-18) | 487 (376-614) |
| Lao People's Democratic Republic | 34 (18-65) | 0.02 (0.01-0.04) | 18 (10-36) | 514 (274-998) |
| Latvia | 357 (293-433) | 0.13 (0.11-0.16) | 95 (81-112) | 2221 (1894-2614) |
| Lebanon | 458 (309-632) | 0.19 (0.13-0.26) | 156 (105-213) | 3387 (2363-4647) |
| Lesotho | 29 (18-43) | 0.02 (0.01-0.03) | 18 (11-28) | 510 (319-780) |
| Liberia | 3 (1-6) | 0.002 (0-0.003) | 1 (0-3) | 43 (12-89) |
| Libya | 271 (177-398) | 0.12 (0.08-0.17) | 97 (61-136) | 2681 (1753-3848) |
| Lithuania | 599 (495-717) | 0.20 (0.18-0.24) | 142 (124-160) | 3352 (2930-3804) |
| Luxembourg | 131 (111-151) | 0.04 (0.04-0.05) | 28 (25-31) | 541 (479-597) |
| Madagascar | 163 (82-242) | 0.1 (0.05-0.14) | 90 (46-133) | 2710 (1358-3988) |
| Malawi | 72 (44-105) | 0.04 (0.03-0.06) | 42 (26-60) | 1186 (708-1719) |
| Malaysia | 662 (506-967) | 0.30 (0.23-0.43) | 246 (195-359) | 6564 (5203-9456) |
| Maldives | 5 (4-7) | 0.002 (0.002-0.003) | 2 (1-2) | 47 (33-66) |
| Mali | 0 (0-0) | 0 (0-0) | 0 (0-0) | 0 (0-0) |
| Malta | 83 (69-98) | 0.03 (0.02-0.03) | 19 (16-22) | 368 (316-421) |
| Marshall Islands | 0 (0-0) | 0 (0-0) | 0 (0-0) | 4 (2-6) |
| Mauritania | 4 (1-8) | 0.003 (0.001-0.004) | 2 (1-4) | 62 (19-106) |
| Mauritius | 85 (74-95) | 0.04 (0.03-0.04) | 31 (29-33) | 813 (743-860) |
| Mexico | 4773 (4126-5374) | 2.01 (1.77-2.27) | 1636 (1450-1841) | 45020 (39620-50814) |
| Mongolia | 15 (11-21) | 0.008 (0.006-0.01) | 7 (5-10) | 239 (168-329) |
| Montenegro | 26 (18-35) | 0.01 (0.009-0.02) | 11 (8-15) | 254 (178-336) |
| Morocco | 627 (364-990) | 0.31 (0.19-0.50) | 279 (172-441) | 7412 (4432-11649) |
| Mozambique | 222 (110-355) | 0.14 (0.07-0.22) | 134 (69-215) | 3905 (1977-6317) |
| Myanmar | 356 (217-668) | 0.19  (0.12-0.37) | 178 (109-344) | 4862 (2966-9280) |
| Namibia | 36 (24-50) | 0.02 (0.01-0.03) | 18 (12-26) | 513 (345-742) |
| Nepal | 321 (161-539) | 0.19 (0.10-0.31) | 178 (91-296) | 4520 (2285-7635) |
| Netherlands | 4680 (4083-5398) | 1.58 (1.38-1.76) | 1188 (1042-1302) | 22592 (20230-24422) |
| New Zealand | 2096 (1803-2402) | 0.51 (0.44-0.58) | 279 (241-308) | 5658 (4998-6190) |
| Nicaragua | 62 (42-83) | 0.03 (0.02-0.04) | 22 (15-28) | 602 (422-780) |
| Niger | 7 (2-14) | 0.004 (0.001-0.01) | 4 (1-9) | 120 (35-229) |
| Nigeria | 1144 (380-1849) | 0.67 (0.24-1.06) | 644 (233-1000) | 16975 (5807-26929) |
| Northern Mariana Islands | 2 (1-2) | 0.001 (0.001-0.001) | 1 (0-1) | 15 (12-22) |
| Norway | 1927 (1684-2171) | 0.57 (0.51-0.63) | 373 (327-403) | 6837 (6183-7311) |
| Oman | 83 (54-128) | 0.03 (0.02-0.04) | 22 (14-31) | 636 (415-946) |
| Pakistan | 3450 (2406-5047) | 2.02 (1.43-2.90) | 1940 (1368-2797) | 53543 (37492-77399) |
| Palestine | 106 (62-142) | 0.05 (0.03-0.06) | 37 (22-49) | 1035 (602-1367) |
| Panama | 279 (215-352) | 0.11 (0.09-0.13) | 86 (68-105) | 2193 (1728-2678) |
| Papua New Guinea | 21 (8-37) | 0.01 (0.004-0.02) | 11 (4-19) | 325 (124-599) |
| Paraguay | 177 (116-253) | 0.08 (0.05-0.12) | 68 (45-99) | 1738 (1156-2458) |
| Peru | 1510 (1042-2206) | 0.61 (0.42-0.86) | 483 (338-693) | 12138 (8456-17526) |
| Philippines | 764 (546-1012) | 0.39 (0.29-0.52) | 349 (261-467) | 9968 (7374-12984) |
| Plurinational State of Bolivia | 305 (206-457) | 0.16 (0.11-0.25) | 151 (103-227) | 3882 (2647-5805) |
| Poland | 3228 (2865-3613) | 1.88 (1.70-2.06) | 1847 (1666-2019) | 38828 (35468-42402) |
| Portugal | 1067 (932-1205) | 0.64 (0.56-0.72) | 662 (566-735) | 12071 (10726-13280) |
| Principality of Monaco | 21 (10-33) | 0.007 (0.003-0.01) | 5 (2-7) | 87 (45-137) |
| Puerto Rico | 1271 (1025-1584) | 0.33 (0.27-0.39) | 186 (154-218) | 4009 (3334-4737) |
| Qatar | 55 (31-98) | 15 (9-25) | 9 (6-15) | 319 (192-525) |
| Republic of Cabo Verde | 1 (0-2) | 0.001 (0-0.001) | 1 (0-1) | 14 (5-23) |
| Republic of C么te d'Ivoire | 232 (142-336) | 0.13 (0.09-0.19) | 127 (82-180) | 3585 (2241-5191) |
| Republic of Korea | 5482 (3085-7443) | 1.67 (0.96-2.21) | 1101 (625-1456) | 23446 (13794-30965) |
| Republic of Moldova | 183 (154-214) | 0.07 (0.06-0.08) | 50 (44-56) | 1314 (1173-1476) |
| Republic of Nauru | 0 (0-0) | 0 (0-0) | 0 (0-0) | 1 (0-2) |
| Republic of Niue | 0 (0-0) | 0 (0-0) | 0 (0-0) | 0 (0-0) |
| Republic of Palau | 0 (0-0) | 0 (0-0) | 0 (0-0) | 0 (0-0) |
| Republic of San Marino | 6 (3-9) | 0.002 (0.001-0.003) | 1 (1-2) | 23 (14-35) |
| Republic of the Gambia | 2 (1-3) | 0.001 (0.001-0.002) | 1 (1-2) | 30 (18-45) |
| Romania | 1030 (862-1193) | 0.54 (0.47-0.62) | 497 (429-562) | 11383 (9768-12834) |
| Russian Federation | 11276 (10225-12265) | 4.35 (4.01-4.72) | 3326 (3066-3592) | 84050 (77360-90885) |
| Rwanda | 144 (80-211) | 0.08 (0.05-0.13) | 81 (47-121) | 2262 (1280-3398) |
| Saint Kitts and Nevis | 7 (5-9) | 0.003 (0.002-0.003) | 2 (1-2) | 46 (36-56) |
| Saint Lucia | 31 (25-38) | 0.01 (0.01-0.01) | 8 (7-10) | 197 (162-239) |
| Saint Vincent and the Grenadines | 9 (7-10) | 0.003 (0.003-0.004) | 3 (2-3) | 64 (56-73) |
| Samoa | 2 (1-3) | 0.001 (0.001-0.001) | 1 (1-1) | 22 (15-35) |
| Sao Tome and Principe | 0 (0-0) | 0 (0-0) | 0 (0-0) | 3 (1-5) |
| Saudi Arabia | 775 (506-1120) | 0.27 (0.18-0.37) | 193 (124-262) | 6317 (4140-8694) |
| Senegal | 13 (4-21) | 0.01 (0.003-0.001) | 7 (2-13) | 196 (62-334) |
| Serbia | 407 (286-541) | 0.27 (0.19-0.36) | 279 (196-366) | 6143 (4283-8093) |
| Seychelles | 4 (3-6) | 0.002 (0.002-0.003) | 2 (1-2) | 46 (34-60) |
| Sierra Leone | 4 (1-7) | 0.003 (0.001-0.005) | 3 (1-4) | 69 (20-123) |
| Singapore | 293 (248-341) | 0.09 (0.08-0.10) | 61 (54-68) | 1311 (1184-1444) |
| Slovakia | 918 (619-1299) | 0.32  (0.22-0.43) | 228 (152-304) | 5206 (3533-7070) |
| Slovenia | 401 (330-479) | 0.15 (0.13-0.18) | 121 (104-141) | 2400 (2056-2803) |
| Socialist Republic of Viet Nam | 714 (493-948) | 0.32 (0.21-0.42) | 261 (175-349) | 6737 (4589-9320) |
| Solomon Islands | 2 (1-3) | 0.001 (0-0.002) | 1 (0-2) | 26 (12-48) |
| Somalia | 50 (20-105) | 0.03 (0.01-0.07) | 31 (13-67) | 939 (370-2036) |
| South Africa | 1951 (1262-2445) | 1.03(0.66-1.27) | 926 (604-1134) | 25640 (16743-32198) |
| South Sudan | 71 (36-114) | 0.04 (0.02-0.07) | 41 (21-65) | 1184 (598-1910) |
| Spain | 14003 (11845-16354) | 3.90 (3.33-4.41) | 2412 (2047-2684) | 44224 (38811-48573) |
| Sri Lanka | 797 (480-1241) | 0.33 (0.20-0.51) | 264 (163-392) | 6617 (3967-9816) |
| Sudan | 248 (147-402) | 0.13 (0.07-0.20) | 112 (66-185) | 3283 (1910-5253) |
| Suriname | 32 (22-45) | 0.001 (0.001-0.02) | 11 (8-15) | 295 (201-405) |
| Sweden | 2032 (1712-2377) | 0.79 (0.68-0.90) | 651 (561-737) | 11022 (9684-12449) |
| Switzerland | 2751 (2317-3237) | 0.78 (0.66-0.90) | 483 (414-547) | 8839 (7742-9927) |
| Syrian Arab Republic | 179 (107-276) | 0.07 (0.05-0.11) | 58 (36-85) | 1590 (959-2366) |
| Taiwan (Province of China) | 2681 (2281-3117) | 0.83 (0.73-0.94) | 544 (490-598) | 13269 (12023-14507) |
| Tajikistan | 27 (17-39) | 0.01 (0.01-0.02) | 13 (9-19) | 456 (301-666) |
| Thailand | 1941 (1208-3726) | 0.77 (0.47-1.44) | 603 (374-1142) | 15301 (9448-28857) |
| The former Yugoslav Republic of Macedonia | 66 (42-89) | 0.04 (0.02-0.05) | 33 (21-43) | 762 (494-1011) |
| Timor-Leste | 4 (2-8) | 0.002 (0.001-0.004) | 2 (1-4) | 57 (34-113) |
| Togo | 6 (2-10) | 0.003 (0.001-0.01) | 3 (1-5) | 87 (24-154) |
| Tokelau | 0 (0-0) | 0 (0-0) | 0 (0-0) | 0 (0-0) |
| Tonga | 3 (2-4) | 0.001 (0.001-0.002) | 1 (1-2) | 34 (23-48) |
| Trinidad and Tobago | 219 (161-285) | 0.08 (0.06-0.1) | 53 (41-68) | 1352 (1015-1749) |
| Tunisia | 535 (329-801) | 0.22 (0.14-0.33) | 180 (112-260) | 4410 (2786-6411) |
| Turkey | 5537 (3785-7665) | 2.22 (1.61-3.06) | 1768 (1299-2436) | 44486 (32418-60248) |
| Turkmenistan | 99 (73-136) | 0.05 (0.04-0.07) | 44 (33-60) | 1530 (1139-2106) |
| Tuvalu | 0 (0-0) | 0 (0-0) | 0 (0-0) | 1 (1-1) |
| Uganda | 746 (498-1159) | 0.44 (0.30-0.67) | 425 (294-651) | 11793 (7851-18724) |
| Ukraine | 2243 (1642-2968) | 0.90 (0.66-1.18) | 703 (511-916) | 19419 (13971-25315) |
| United Arab Emirates | 279 (182-421) | 0.11 (0.07-0.15) | 79 (54-111) | 2665 (1814-3864) |
| United Kingdom of Great Britain and Northern Ireland | 26053 (24224-27444) | 6.98 (6.35-7.34) | 4102 (3698-4304) | 76844 (71548-80593) |
| United Republic of Tanzania | 581 (327-866) | 0.34 (0.20-0.51) | 329 (188-487) | 9084 (5069-13717) |
| United States of America | 30695 (28513-32090) | 17.69 (16.12-18.61) | 17370 (15720-18340) | 335912 (313409-350499) |
| United States Virgin Islands | 18 (11-28) | 0.006 (0.004-0.01) | 4 (2-6) | 93 (57-150) |
| Uruguay | 433 (378-487) | 0.20 (0.18-0.22) | 168 (152-184) | 3505 (3210-3823) |
| Uzbekistan | 121 (88-161) | 0.06 (0.05-0.08) | 55 (42-70) | 1812 (1358-2327) |
| Vanuatu | 1 (0-1) | 0.001 (0-0.001) | 0 (0-1) | 15 (7-24) |
| Yemen | 130 (69-226) | 0.07 (0.04-0.13) | 65 (35-116) | 1877 (986-3288) |
| Zambia | 296 (118-544) | 0.17 (0.07-0.03) | 159 (66-279) | 4834 (1871-8851) |
| Zimbabwe | 408 (222-596) | 0.25 (0.13-0.36) | 233 (128-336) | 6892 (3793-9955) |

***UI*** uncertainty interval

**TableS5**. **ASPR, ASIR, ASMR, and ASDR for multiple myeloma (MM) in 204 countries in 2021 and their variation trends from 1990 to 2021**

| Country | ASPR/100,000 (95% UI) | EAPC of ASPR (95% UI) | ASIR/100,000 (95% UI) | EAPC of ASIR (95% UI) | ASMR/100,000 (95% UI) | EAPC of ASMR (95% CI) | ASDR/100,000 persons（95% UI） | EAPC of ASDR (95% CI) |
| --- | --- | --- | --- | --- | --- | --- | --- | --- |
| Afghanistan | 1.01 (0.42-2.19) | 1.47 (1.13-1.82) | 0.65 (0.27-1.37) | 1.15 (0.85-1.46) | 0.64 (0.27-1.34) | 1.07 (0.78-1.36) | 16.66 (6.86-35.61) | 1.04 (0.75-1.32) |
| Albania | 0.99 (0.63-1.39) | 2.89 (2.65-3.14) | 0.54 (0.35-0.76) | 2.05 (1.87-2.24) | 0.51 (0.33-0.71) | 1.71 (1.54-1.88) | 11.07 (7.24-15.35) | 1.64 (1.46-1.82) |
| Algeria | 2.64 (1.75-3.8) | 2.23 (2.1-2.36) | 1.3 (0.81-1.79) | 1.36 (1.23-1.49) | 1.18 (0.72-1.62) | 1.02 (0.88-1.16) | 25.79 (16.29-34.87) | 0.9 (0.8-1) |
| American Samoa | 2.22 (1.6-3.09) | 0.91 (0.66-1.16) | 1.2 (0.86-1.61) | 0.68 (0.43-0.93) | 1.11 (0.8-1.47) | 0.59 (0.34-0.85) | 27.53 (20.16-37.13) | 0.62 (0.36-0.88) |
| Andorra | 8.93 (5.54-13.03) | 1.27 (0.97-1.56) | 2.51 (1.64-3.66) | 0.43 (0.21-0.65) | 1.57 (1.05-2.21) | -0.19 (-0.39-0.01) | 33.47 (21.97-47.6) | -0.24 (-0.44--0.05) |
| Angola | 0.9 (0.55-1.3) | 2.11 (1.92-2.31) | 0.59 (0.36-0.84) | 1.72 (1.56-1.88) | 0.59 (0.36-0.84) | 1.62 (1.47-1.77) | 14.62 (8.96-21.25) | 1.55 (1.39-1.7) |
| Antigua and Barbuda | 12.81 (10.96-14.8) | 1.81 (1.57-2.05) | 4.24 (3.81-4.79) | 1.05 (0.81-1.29) | 2.87 (2.63-3.11) | 0.57 (0.33-0.82) | 66.61 (61.13-72.52) | 0.48 (0.26-0.7) |
| Argentina | 4.26 (3.71-4.83) | 0.62 (0.43-0.82) | 1.9 (1.72-2.07) | -0.12 (-0.28-0.04) | 2.84 (2.46-3.2) | 0.14 (0.02-0.25) | 37.91 (35.14-40.71) | -0.49 (-0.64--0.35) |
| Armenia | 2.04 (1.62-2.48) | 5.16 (4.47-5.85) | 0.96 (0.8-1.17) | 4.51 (3.87-5.16) | 0.84 (0.68-1) | 4.23 (3.61-4.85) | 23.4 (18.96-27.89) | 4.14 (3.51-4.77) |
| Australia | 22.75 (19.29-26.69) | 2.29 (1.99-2.6) | 5.38 (4.61-6.21) | 1.13 (0.98-1.27) | 2.84 (2.46-3.2) | 0.14 (0.02-0.25) | 59.3 (52.35-66.69) | -0.07 (-0.17-0.03) |
| Austria | 11.76 (10.07-13.7) | 1.38 (1.02-1.74) | 3.37 (2.94-3.8) | 0.64 (0.41-0.87) | 2.15 (1.9-2.35) | 0.16 (0.02-0.31) | 43.81 (39.55-47.79) | -0.13 (-0.3-0.05) |
| Azerbaijan | 0.51 (0.31-0.75) | 2.24 (1.76-2.73) | 0.27 (0.17-0.4) | 1.9 (1.47-2.34) | 0.24 (0.15-0.36) | 1.76 (1.34-2.18) | 7.12 (4.47-10.61) | 1.49 (1.07-1.92) |
| Bahrain | 5.19 (3.11-7.46) | 1.98 (1.89-2.06) | 2.24 (1.36-3.18) | 0.66 (0.49-0.82) | 1.85 (1.15-2.6) | 0.1 (-0.1-0.3) | 41.25 (24.46-57.9) | -0.02 (-0.17-0.13) |
| Bangladesh | 1.42 (0.84-2.44) | 1.67 (1.54-1.81) | 0.8 (0.49-1.38) | 0.96 (0.85-1.07) | 0.77 (0.47-1.33) | 0.71 (0.6-0.82) | 18.21 (10.76-31.13) | 0.61 (0.51-0.7) |
| Barbados | 9.89 (7.41-12.99) | 1.53 (1.34-1.73) | 3.12 (2.33-4.09) | 0.82 (0.69-0.96) | 2.06 (1.56-2.61) | 0.34 (0.25-0.44) | 48.88 (36.55-62.08) | 0.32 (0.22-0.41) |
| Belarus | 7.84 (6.05-9.73) | 4.89 (4.73-5.06) | 2.49 (2-3.07) | 3.58 (3.39-3.78) | 1.64 (1.32-1.98) | 2.74 (2.5-2.99) | 43.01 (34.22-52.81) | 2.74 (2.46-3.02) |
| Belgium | 13.08 (10.92-15.17) | 1.23 (0.96-1.51) | 4 (3.49-4.52) | 0.25 (0.09-0.4) | 2.69 (2.35-2.96) | -0.37 (-0.48--0.25) | 54.91 (49.32-60.29) | -0.47 (-0.64--0.31) |
| Belize | 2.86 (2.41-3.43) | 1.58 (1.3-1.86) | 1.07 (0.93-1.23) | 0.89 (0.56-1.22) | 0.8 (0.7-0.9) | 0.5 (0.14-0.86) | 20.13 (17.6-22.97) | 0.58 (0.24-0.93) |
| Benin | 0.13 (0.04-0.21) | 3.67 (3.52-3.82) | 0.09 (0.03-0.14) | 3.44 (3.28-3.61) | 0.09 (0.03-0.14) | 3.4 (3.23-3.57) | 2.08 (0.64-3.4) | 3.26 (3.11-3.42) |
| Bermuda | 21.45 (17.3-27.4) | 1.55 (1.14-1.97) | 4.93 (4.06-6.36) | 0.28 (0.01-0.55) | 2.49 (2.09-3.11) | -0.73 (-0.92--0.54) | 57.64 (47.97-72.25) | -0.76 (-0.95--0.57) |
| Bhutan | 1.84 (0.92-3.3) | 2.4 (2.3-2.51) | 1.09 (0.56-1.99) | 1.85 (1.77-1.93) | 1.07 (0.56-1.93) | 1.65 (1.59-1.72) | 24.58 (12.43-44.49) | 1.48 (1.41-1.54) |
| Bolivarian Republic of Venezuela | 3.89 (2.79-5.25) | 1.49 (1.23-1.75) | 1.73 (1.27-2.28) | 0.78 (0.57-0.99) | 1.45 (1.08-1.9) | 0.44 (0.25-0.63) | 37.31 (27.45-49.43) | 0.41 (0.23-0.6) |
| Bosnia and Herzegovina | 2.5 (1.68-3.35) | 2.82 (2.58-3.06) | 1.29 (0.87-1.72) | 2.02 (1.87-2.17) | 1.18 (0.8-1.59) | 1.68 (1.57-1.79) | 27.36 (18.46-36.36) | 1.63 (1.49-1.77) |
| Botswana | 3.11 (2.11-4.59) | 2 (1.87-2.13) | 1.93 (1.35-2.82) | 1.66 (1.46-1.85) | 1.88 (1.33-2.78) | 1.56 (1.35-1.78) | 46.7 (31.86-68.3) | 1.42 (1.19-1.65) |
| Brazil | 4.65 (4.35-4.92) | 2.08 (1.87-2.3) | 2.11 (1.96-2.23) | 1.48 (1.31-1.66) | 1.81 (1.67-1.91) | 1.23 (1.08-1.39) | 43.77 (41.32-45.58) | 0.97 (0.81-1.13) |
| Brunei Darussalam | 4.55 (3.23-6.08) | 0.93 (0.78-1.07) | 2.39 (1.68-3.18) | 0.33 (0.15-0.5) | 2.16 (1.53-2.82) | 0.12 (-0.07-0.31) | 48.01 (34.64-62.54) | -0.05 (-0.21-0.11) |
| Bulgaria | 3.68 (2.81-5.03) | 3.03 (2.8-3.27) | 1.49 (1.17-1.95) | 2.71 (2.47-2.94) | 1.19 (0.92-1.54) | 2.52 (2.26-2.78) | 29.66 (22.91-38.34) | 2.47 (2.23-2.71) |
| Burkina Faso | 0.13 (0.04-0.22) | 4.17 (4.01-4.33) | 0.09 (0.03-0.15) | 3.99 (3.82-4.15) | 0.09 (0.03-0.15) | 3.96 (3.79-4.12) | 2.13 (0.72-3.58) | 3.84 (3.7-3.99) |
| Burundi | 1.2 (0.56-2.28) | -0.86 (-1.07--0.66) | 0.82 (0.39-1.55) | -1.19 (-1.39--0.99) | 0.84 (0.4-1.6) | -1.2 (-1.4--1.01) | 20.4 (9.5-39.41) | -1.34 (-1.55--1.13) |
| Cambodia | 0.8 (0.45-1.6) | 2.65 (2.41-2.89) | 0.46 (0.26-0.96) | 2.1 (1.9-2.31) | 0.44 (0.25-0.92) | 1.89 (1.69-2.08) | 11.09 (6.24-22.84) | 1.8 (1.62-1.99) |
| Cameroon | 0.2 (0.06-0.33) | 3.67 (3.52-3.82) | 0.13 (0.04-0.21) | 3.45 (3.29-3.61) | 0.13 (0.04-0.22) | 3.4 (3.23-3.57) | 3.12 (0.97-5.14) | 3.3 (3.14-3.47) |
| Canada | 15.9 (13.75-18.49) | 0.53 (0.27-0.79) | 4.32 (3.8-4.87) | -0.12 (-0.28-0.03) | 2.6 (2.31-2.85) | -0.58 (-0.68--0.48) | 52.98 (48.39-57.47) | -0.94 (-1.05--0.84) |
| Central African Republic | 0.5 (0.28-0.76) | 0.12 (0.07-0.17) | 0.36 (0.19-0.55) | -0.01 (-0.07-0.05) | 0.37 (0.2-0.55) | -0.02 (-0.08-0.04) | 9.64 (5.22-14.66) | -0.05 (-0.11-0.01) |
| Chad | 0.11 (0.04-0.18) | 4.55 (4.33-4.78) | 0.08 (0.03-0.12) | 4.44 (4.2-4.69) | 0.08 (0.03-0.13) | 4.43 (4.18-4.69) | 1.85 (0.64-3.1) | 4.35 (4.1-4.61) |
| Chile | 8.18 (7.1-9.42) | 1.99 (1.71-2.28) | 3.01 (2.71-3.34) | 0.66 (0.41-0.91) | 2.29 (2.09-2.5) | -0.02 (-0.27-0.23) | 52.51 (48.53-57.04) | -0.17 (-0.41-0.07) |
| China | 2.19 (1.37-2.9) | 5.96 (5.35-6.56) | 0.81 (0.52-1.07) | 4.05 (3.38-4.73) | 0.62 (0.4-0.81) | 3.11 (2.4-3.83) | 16.12 (10.09-21.35) | 3.15 (2.47-3.84) |
| Colombia | 5.13 (4.12-6.32) | 2.25 (2.03-2.47) | 1.99 (1.64-2.39) | 1.12 (0.91-1.32) | 1.55 (1.28-1.87) | 0.55 (0.34-0.75) | 38.78 (32.14-47.05) | 0.45 (0.25-0.65) |
| Commonwealth of the Bahamas | 18.36 (14.05-23.46) | 1.29 (1.09-1.48) | 6.55 (5.23-8.18) | 0.58 (0.49-0.68) | 4.71 (3.86-5.7) | 0.16 (0.12-0.21) | 117.52 (94.29-145.13) | 0.1 (0.06-0.14) |
| Comoros | 2.06 (1.08-3.03) | 0.55 (0.44-0.65) | 1.33 (0.72-1.95) | 0.3 (0.2-0.39) | 1.34 (0.73-1.98) | 0.25 (0.16-0.34) | 32.41 (17.3-47.83) | 0.1 (-0.02-0.23) |
| Congo | 1.13 (0.7-1.69) | 1.13 (0.94-1.31) | 0.72 (0.46-1.07) | 0.7 (0.54-0.86) | 0.71 (0.45-1.06) | 0.59 (0.44-0.74) | 17.73 (11.24-26.58) | 0.49 (0.34-0.65) |
| Cook Islands | 0.79 (0.55-1.09) | 2.26 (2.04-2.49) | 0.33 (0.23-0.44) | 1.29 (1.07-1.5) | 0.26 (0.18-0.35) | 0.82 (0.62-1.02) | 6.36 (4.38-8.66) | 0.89 (0.67-1.11) |
| Costa Rica | 9.39 (7.71-11.24) | 3.08 (2.87-3.28) | 3.34 (2.84-3.82) | 2.1 (1.91-2.3) | 2.47 (2.16-2.77) | 1.55 (1.36-1.73) | 63.78 (55.89-71.8) | 1.81 (1.61-2) |
| Croatia | 10.84 (8.71-13.74) | 2.55 (2.29-2.82) | 3.42 (2.85-4.11) | 1.58 (1.38-1.78) | 2.3 (1.96-2.7) | 0.97 (0.79-1.15) | 51.4 (43.86-61.06) | 0.85 (0.65-1.05) |
| Cuba | 13.08 (10.87-15.83) | 1.73 (1.59-1.87) | 3.55 (3.03-4.14) | 0.9 (0.81-1) | 2.06 (1.77-2.34) | 0.26 (0.18-0.34) | 50.61 (43.84-57.7) | 0.35 (0.26-0.45) |
| Cyprus | 11.53 (7.17-16.25) | 2.9 (2.48-3.33) | 3.55 (2.33-4.71) | 0.86 (0.61-1.11) | 2.42 (1.55-3.24) | -0.3 (-0.47--0.12) | 48.47 (31.31-64.99) | -0.17 (-0.36-0.01) |
| Czech Republic | 8.37 (6.9-10.11) | 1.62 (1.25-1.99) | 3.04 (2.55-3.55) | 0.48 (0.28-0.68) | 2.26 (1.89-2.63) | -0.14 (-0.29-0.01) | 50.83 (43.16-58.58) | -0.31 (-0.46--0.17) |
| Democratic People's Republic of Korea | 0.73 (0.43-1.25) | 1.23 (1.06-1.4) | 0.34 (0.19-0.58) | 0.57 (0.47-0.68) | 0.29 (0.16-0.5) | 0.29 (0.2-0.38) | 8.04 (4.58-13.74) | 0.31 (0.23-0.39) |
| Democratic Republic of the Congo | 0.52 (0.19-0.86) | 0.45 (0.01-0.88) | 0.34 (0.13-0.57) | 0.18 (-0.21-0.57) | 0.34 (0.12-0.57) | 0.11 (-0.27-0.49) | 8.47 (3.09-14.34) | 0.11 (-0.27-0.49) |
| Denmark | 15.98 (13.81-18.32) | 2.63 (2.06-3.21) | 4.49 (3.93-5.06) | 1.41 (1.02-1.81) | 2.77 (2.49-3.08) | 0.55 (0.21-0.88) | 54.88 (49.93-60.38) | 0.05 (-0.29-0.38) |
| Djibouti | 2.47 (1.38-3.79) | 0.95 (0.86-1.03) | 1.59 (0.91-2.39) | 0.71 (0.66-0.76) | 1.59 (0.92-2.41) | 0.64 (0.59-0.68) | 38.56 (21.88-59.24) | 0.56 (0.51-0.61) |
| Dominica | 7.66 (5.09-10.95) | 0.79 (0.62-0.96) | 3.19 (2.14-4.46) | 0.49 (0.38-0.6) | 2.53 (1.73-3.46) | 0.32 (0.23-0.4) | 60.28 (41.81-83.74) | 0.45 (0.37-0.54) |
| Dominican Republic | 4.9 (3.28-6.81) | 3.03 (2.8-3.26) | 1.96 (1.37-2.68) | 2.41 (2.25-2.58) | 1.53 (1.07-2.09) | 2.02 (1.88-2.16) | 38.32 (26.81-53.27) | 2.09 (1.99-2.2) |
| Ecuador | 3.88 (2.96-4.97) | 4.57 (4.21-4.93) | 1.78 (1.4-2.28) | 3.8 (3.49-4.11) | 1.53 (1.22-1.91) | 3.43 (3.13-3.74) | 37.29 (29.45-47.22) | 3.32 (3.02-3.62) |
| Egypt | 0.83 (0.52-1.55) | 2.17 (2.02-2.32) | 0.43 (0.28-0.81) | 1.49 (1.32-1.65) | 0.39 (0.25-0.74) | 1.17 (0.99-1.35) | 9.55 (6.16-17.39) | 1.18 (1.01-1.35) |
| El Salvador | 1.64 (1.08-2.28) | 2.95 (2.78-3.12) | 0.68 (0.46-0.9) | 1.87 (1.77-1.98) | 0.55 (0.38-0.73) | 1.34 (1.25-1.44) | 14.74 (9.79-19.67) | 1.38 (1.28-1.48) |
| Equatorial Guinea | 1.76 (0.98-2.82) | 4.85 (4.58-5.11) | 1.03 (0.59-1.62) | 4.03 (3.79-4.26) | 0.99 (0.57-1.54) | 3.75 (3.52-3.99) | 24.4 (13.69-38.96) | 3.57 (3.35-3.79) |
| Eritrea | 1.71 (0.9-2.58) | 0.77 (0.58-0.96) | 1.16 (0.61-1.73) | 0.52 (0.33-0.71) | 1.18 (0.62-1.78) | 0.5 (0.3-0.69) | 29.33 (15.38-44.34) | 0.35 (0.15-0.54) |
| Estonia | 16.64 (13.56-20.12) | 4.26 (3.97-4.56) | 4.59 (3.84-5.43) | 2.73 (2.44-3.02) | 2.72 (2.33-3.1) | 1.68 (1.37-1.98) | 67.13 (57.54-77.14) | 1.48 (1.12-1.85) |
| Ethiopia | 1.01 (0.51-1.78) | 1.6 (1.21-1.99) | 0.66 (0.34-1.17) | 1.08 (0.72-1.44) | 0.67 (0.34-1.18) | 0.97 (0.63-1.32) | 15.64 (7.91-27.81) | 0.75 (0.4-1.1) |
| Federated States of Micronesia | 0.65 (0.33-1.07) | 0.79 (0.74-0.83) | 0.39 (0.2-0.62) | 0.42 (0.35-0.48) | 0.38 (0.19-0.59) | 0.28 (0.2-0.36) | 9.72 (4.85-15.69) | 0.29 (0.23-0.36) |
| Fiji | 1.45 (0.93-2.04) | 1.43 (1.27-1.59) | 0.88 (0.58-1.2) | 1.21 (1.09-1.34) | 0.85 (0.57-1.15) | 1.15 (1.03-1.27) | 20.73 (13.49-28.91) | 1.21 (1.05-1.36) |
| Finland | 15.1 (12.95-17.36) | 1.49 (1.26-1.72) | 4.12 (3.61-4.61) | 0.52 (0.36-0.67) | 2.49 (2.2-2.76) | -0.21 (-0.35--0.06) | 51.63 (46.68-56.42) | -0.46 (-0.61--0.32) |
| France | 13.95 (11.75-16.48) | 1.94 (1.64-2.24) | 4.14 (3.57-4.81) | 0.69 (0.46-0.93) | 2.75 (2.35-3.11) | -0.09 (-0.3-0.13) | 55.48 (48.94-62.46) | -0.3 (-0.53--0.08) |
| Gabon | 2.13 (1.19-3.09) | 1.2 (1.1-1.3) | 1.28 (0.72-1.84) | 0.78 (0.71-0.85) | 1.24 (0.71-1.78) | 0.64 (0.58-0.7) | 30.9 (17.37-45.17) | 0.56 (0.5-0.63) |
| Georgia | 2.96 (2.47-3.52) | 6.31 (5.56-7.07) | 1.48 (1.26-1.73) | 6.2 (5.45-6.96) | 1.32 (1.14-1.53) | 6.18 (5.43-6.95) | 38.46 (33.11-44.33) | 5.98 (5.23-6.73) |
| Germany | 17.27 (15.01-19.6) | 1.51 (0.95-2.07) | 4.34 (3.83-4.79) | 0.85 (0.51-1.19) | 2.4 (2.14-2.6) | 0.28 (0.09-0.47) | 50.73 (46.4-54.62) | 0.04 (-0.17-0.24) |
| Ghana | 0.2 (0.06-0.4) | 5.18 (4.98-5.38) | 0.13 (0.04-0.25) | 4.98 (4.76-5.21) | 0.13 (0.04-0.25) | 4.93 (4.69-5.17) | 3.04 (0.87-5.98) | 4.75 (4.52-4.98) |
| Greece | 11.37 (9.85-13.04) | 1.87 (1.68-2.07) | 3.58 (3.23-3.97) | 1.31 (1.17-1.44) | 2.46 (2.25-2.62) | 0.94 (0.84-1.05) | 51.6 (47.75-54.76) | 0.87 (0.79-0.95) |
| Greenland | 5.66 (4.18-8.28) | 0.84 (0.69-1) | 2.51 (1.85-3.67) | -0.17 (-0.32--0.03) | 2.09 (1.57-3.09) | -0.63 (-0.78--0.49) | 48.31 (36.76-70.67) | -0.69 (-0.82--0.56) |
| Grenada | 12.63 (10.42-15.11) | 2.72 (2.36-3.08) | 4.81 (4.13-5.54) | 1.99 (1.63-2.35) | 3.57 (3.1-4.04) | 1.58 (1.24-1.93) | 84.44 (73.31-96.69) | 1.49 (1.21-1.77) |
| Guam | 1.38 (1.03-1.86) | 0.61 (0.32-0.9) | 0.58 (0.43-0.8) | -0.05 (-0.33-0.22) | 0.47 (0.35-0.64) | -0.39 (-0.68--0.1) | 13.36 (9.96-17.61) | 0.17 (-0.09-0.43) |
| Guatemala | 1.06 (0.87-1.26) | 1.94 (1.84-2.03) | 0.53 (0.45-0.62) | 1.08 (0.97-1.2) | 0.47 (0.4-0.55) | 0.7 (0.57-0.82) | 12.61 (10.75-14.78) | 0.85 (0.71-0.98) |
| Guinea | 0.34 (0.18-0.51) | 2.05 (2-2.1) | 0.23 (0.13-0.36) | 1.89 (1.82-1.95) | 0.24 (0.14-0.36) | 1.81 (1.75-1.88) | 5.67 (3.12-8.71) | 1.85 (1.77-1.93) |
| Guinea-Bissau | 0.15 (0.05-0.23) | 3.34 (3.2-3.49) | 0.1 (0.04-0.16) | 3.12 (2.97-3.27) | 0.11 (0.04-0.16) | 3.09 (2.93-3.24) | 2.58 (0.91-4.04) | 2.99 (2.83-3.14) |
| Guyana | 2.94 (2.25-3.92) | 4.09 (3.12-5.07) | 1.32 (1.02-1.71) | 3.29 (2.42-4.16) | 1.08 (0.84-1.38) | 2.95 (2.12-3.78) | 27.9 (21.29-36.26) | 2.98 (2.17-3.79) |
| Haiti | 2.37 (1.35-4.63) | 0.86 (0.77-0.96) | 1.36 (0.76-2.55) | 0.46 (0.41-0.51) | 1.27 (0.71-2.36) | 0.3 (0.25-0.35) | 31.03 (17.51-58.78) | 0.26 (0.2-0.31) |
| Honduras | 2.32 (1.5-3.46) | 2.47 (2.34-2.59) | 1.27 (0.83-1.87) | 2.19 (2.07-2.3) | 1.19 (0.78-1.73) | 2.04 (1.93-2.16) | 29.69 (19.34-44.03) | 1.88 (1.77-1.98) |
| Hungary | 4.15 (3.45-4.9) | 0.94 (0.63-1.25) | 1.97 (1.68-2.29) | 0.28 (0.01-0.55) | 1.72 (1.48-1.98) | 0.01 (-0.24-0.26) | 40.1 (34.21-46.58) | -0.18 (-0.42-0.07) |
| Iceland | 16.75 (14.07-19.81) | 1.41 (1.07-1.75) | 4.56 (3.92-5.25) | 0.63 (0.39-0.86) | 2.79 (2.39-3.13) | 0.07 (-0.1-0.24) | 57.5 (50.79-64.34) | -0.19 (-0.35--0.03) |
| India | 1.88 (1.45-2.44) | 2.44 (2.2-2.67) | 1.06 (0.83-1.4) | 1.82 (1.62-2.02) | 1.01 (0.8-1.34) | 1.61 (1.43-1.8) | 24.22 (19.08-32.16) | 1.46 (1.27-1.64) |
| Indonesia | 0.8 (0.51-1.48) | 1.98 (1.91-2.04) | 0.44 (0.28-0.84) | 1.64 (1.6-1.68) | 0.42 (0.26-0.79) | 1.5 (1.46-1.55) | 10.37 (6.7-19.35) | 1.38 (1.33-1.43) |
| Iraq | 2.97 (1.91-4.34) | 2.71 (2.39-3.02) | 1.39 (0.92-1.98) | 1.87 (1.62-2.12) | 1.2 (0.79-1.69) | 1.48 (1.27-1.69) | 29.64 (19.4-42.24) | 1.31 (1.13-1.5) |
| Ireland | 14.66 (12.3-17.19) | 1.67 (1.41-1.93) | 4.18 (3.56-4.79) | 0.19 (0.02-0.37) | 2.65 (2.31-2.97) | -0.73 (-0.89--0.57) | 54.29 (48.24-60.89) | -0.93 (-1.1--0.76) |
| Islamic Republic of Iran | 2.41 (1.47-3.1) | 3.38 (3.24-3.51) | 0.98 (0.63-1.21) | 2.35 (2.22-2.48) | 0.79 (0.51-0.96) | 1.84 (1.71-1.98) | 19.38 (12.38-23.03) | 1.75 (1.61-1.89) |
| Israel | 12.52 (10.52-14.54) | 1.75 (1.45-2.05) | 4.05 (3.55-4.55) | 0.5 (0.28-0.71) | 2.87 (2.51-3.15) | -0.22 (-0.41--0.03) | 58.31 (52.47-63.56) | -0.34 (-0.54--0.13) |
| Italy | 17.87 (15.53-20.2) | 1.64 (1.16-2.11) | 4.55 (4.04-5.05) | 0.85 (0.56-1.15) | 2.59 (2.31-2.78) | 0.23 (0.07-0.38) | 54.38 (49.67-57.99) | -0.06 (-0.22-0.09) |
| Jamaica | 14.38 (10.65-18.87) | 3.52 (3.04-4) | 4.97 (3.82-6.51) | 3.06 (2.6-3.52) | 3.5 (2.7-4.48) | 2.73 (2.29-3.18) | 83.97 (63.83-108.33) | 2.64 (2.18-3.09) |
| Japan | 6.42 (5.5-7.34) | 0.94 (0.59-1.28) | 1.97 (1.72-2.17) | -0.21 (-0.4--0.03) | 1.3 (1.12-1.4) | -0.92 (-1.06--0.79) | 25.68 (23.2-27.3) | -1.24 (-1.39--1.09) |
| Jordan | 2.85 (1.76-4.25) | 1.22 (1.04-1.41) | 1.19 (0.75-1.75) | -0.03 (-0.23-0.18) | 0.97 (0.62-1.42) | -0.62 (-0.85--0.39) | 23.48 (14.68-34.26) | -0.68 (-0.91--0.45) |
| Kazakhstan | 1.42 (1.16-1.74) | 2.06 (1.64-2.48) | 0.69 (0.57-0.81) | 1.45 (1.13-1.76) | 0.6 (0.5-0.7) | 1.19 (0.9-1.48) | 17.74 (14.84-20.9) | 1 (0.76-1.25) |
| Kenya | 2.11 (1.35-2.9) | 2.35 (2.21-2.48) | 1.33 (0.85-1.81) | 2.15 (2.06-2.24) | 1.32 (0.85-1.8) | 2.09 (1.99-2.18) | 31.74 (20.45-43.87) | 2.11 (2.02-2.2) |
| Kingdom of Eswatini | 3.85 (2.49-5.73) | 2.19 (1.94-2.44) | 2.43 (1.62-3.59) | 2.08 (1.71-2.46) | 2.37 (1.6-3.52) | 2 (1.62-2.39) | 62.53 (40.69-96.05) | 2.08 (1.66-2.51) |
| Kiribati | 0.1 (0.06-0.15) | 0.38 (0.31-0.46) | 0.07 (0.04-0.1) | 0.23 (0.16-0.31) | 0.07 (0.04-0.1) | 0.21 (0.14-0.29) | 1.76 (0.96-2.57) | 0.17 (0.09-0.25) |
| Kuwait | 3.3 (2.62-4.07) | 1.25 (0.44-2.06) | 1.11 (0.9-1.33) | 0.16 (-0.67-1.01) | 0.8 (0.65-0.95) | -0.44 (-1.29-0.42) | 19.4 (15.88-23.06) | -0.46 (-1.23-0.31) |
| Kyrgyzstan | 0.63 (0.48-0.81) | 4.07 (2.92-5.23) | 0.31 (0.24-0.4) | 3.37 (2.17-4.58) | 0.28 (0.22-0.35) | 3.07 (1.84-4.32) | 8.48 (6.55-10.6) | 3.28 (2.25-4.32) |
| Lao People's Democratic Republic | 0.68 (0.36-1.31) | 2.18 (2.06-2.3) | 0.41 (0.22-0.82) | 1.72 (1.63-1.81) | 0.4 (0.22-0.8) | 1.57 (1.5-1.64) | 10.11 (5.42-19.95) | 1.46 (1.39-1.53) |
| Latvia | 10.03 (8.19-12.22) | 3.75 (3.45-4.05) | 3.47 (2.91-4.14) | 2.79 (2.54-3.04) | 2.44 (2.07-2.86) | 2.23 (2.02-2.45) | 63.11 (53.82-74.68) | 2.24 (2.01-2.48) |
| Lebanon | 7.81 (5.3-10.79) | 2.44 (2.23-2.66) | 3.15 (2.16-4.26) | 1.18 (0.98-1.37) | 2.53 (1.71-3.46) | 0.57 (0.38-0.77) | 57.04 (40.04-78.48) | 0.41 (0.24-0.59) |
| Lesotho | 2.46 (1.56-3.76) | 3.01 (2.78-3.24) | 1.69 (1.09-2.59) | 3.14 (2.84-3.43) | 1.7 (1.1-2.57) | 3.1 (2.8-3.4) | 43.74 (27.53-67.44) | 3.21 (2.88-3.54) |
| Liberia | 0.12 (0.03-0.24) | 3.6 (3.22-3.97) | 0.08 (0.02-0.15) | 3.17 (2.82-3.53) | 0.08 (0.02-0.15) | 3.05 (2.7-3.41) | 1.82 (0.52-3.69) | 3.01 (2.66-3.36) |
| Libya | 4.68 (3.04-6.77) | 1.82 (1.49-2.16) | 2.23 (1.42-3.09) | 1.11 (0.84-1.38) | 1.96 (1.23-2.7) | 0.78 (0.54-1.03) | 47.29 (29.98-66.92) | 0.74 (0.5-0.98) |
| Lithuania | 11.69 (9.64-14.05) | 3.04 (2.87-3.22) | 3.75 (3.21-4.37) | 2.25 (2.1-2.4) | 2.52 (2.2-2.84) | 1.76 (1.61-1.9) | 65.57 (57.19-74.52) | 1.8 (1.62-1.98) |
| Luxembourg | 12.64 (10.73-14.68) | 1.87 (1.5-2.24) | 3.79 (3.3-4.3) | 0.61 (0.36-0.85) | 2.51 (2.23-2.77) | -0.17 (-0.36-0.01) | 51.18 (45.39-56.47) | -0.37 (-0.57--0.18) |
| Madagascar | 1.29 (0.67-1.89) | -0.12 (-0.34-0.1) | 0.85 (0.45-1.25) | -0.3 (-0.5--0.1) | 0.86 (0.45-1.27) | -0.35 (-0.55--0.15) | 21.19 (10.84-31.4) | -0.39 (-0.58--0.19) |
| Malawi | 0.91 (0.55-1.3) | 1.56 (1.52-1.61) | 0.6 (0.37-0.85) | 1.19 (1.13-1.25) | 0.61 (0.37-0.87) | 1.1 (1.04-1.17) | 14.68 (8.89-20.93) | 1.08 (1.01-1.16) |
| Malaysia | 2.2 (1.68-3.23) | 2.33 (2.22-2.44) | 1.02 (0.81-1.49) | 1.55 (1.41-1.7) | 0.87 (0.69-1.29) | 1.2 (1.03-1.36) | 21.91 (17.37-31.69) | 1.17 (1.05-1.29) |
| Maldives | 1.42 (1.01-2.02) | 2.03 (1.9-2.16) | 0.64 (0.44-0.89) | 0.74 (0.62-0.85) | 0.55 (0.37-0.75) | 0.17 (0.04-0.3) | 12.64 (8.98-16.96) | 0.04 (-0.09-0.16) |
| Mali | 0 (0-0) | 1.42 (1.34-1.51) | 0 (0-0) | 1.19 (1.11-1.28) | 0 (0-0) | 1.14 (1.05-1.22) | 0 (0-0) | 1.06 (0.98-1.14) |
| Malta | 9.14 (7.59-10.71) | 1.84 (1.62-2.07) | 2.75 (2.34-3.18) | 0.58 (0.41-0.75) | 1.83 (1.56-2.1) | -0.19 (-0.35--0.04) | 38.65 (33.3-43.69) | -0.19 (-0.34--0.04) |
| Marshall Islands | 0.57 (0.28-0.99) | 0.87 (0.84-0.9) | 0.36 (0.17-0.61) | 0.69 (0.63-0.74) | 0.35 (0.17-0.59) | 0.64 (0.58-0.71) | 8.94 (4.34-15.57) | 0.62 (0.55-0.69) |
| Mauritania | 0.2 (0.06-0.34) | 3.97 (3.84-4.1) | 0.12 (0.04-0.2) | 3.52 (3.39-3.66) | 0.12 (0.04-0.2) | 3.39 (3.25-3.53) | 2.76 (0.85-4.68) | 3.18 (3.05-3.32) |
| Mauritius | 4.43 (3.88-5) | 4.1 (3.18-5.02) | 1.99 (1.82-2.16) | 3.59 (2.7-4.49) | 1.67 (1.54-1.77) | 3.39 (2.51-4.28) | 42.85 (39.23-45.21) | 3.41 (2.52-4.3) |
| Mexico | 3.59 (3.1-4.03) | 1.83 (1.65-2.02) | 1.55 (1.37-1.74) | 1.1 (0.98-1.23) | 1.28 (1.14-1.44) | 0.74 (0.64-0.84) | 33.95 (29.91-38.28) | 0.78 (0.68-0.89) |
| Mongolia | 0.54 (0.37-0.73) | 2.85 (2.61-3.09) | 0.3 (0.21-0.41) | 2.19 (2.02-2.36) | 0.28 (0.2-0.38) | 1.93 (1.79-2.08) | 8.4 (5.91-11.5) | 2.01 (1.86-2.16) |
| Montenegro | 2.64 (1.82-3.58) | 1.76 (1.49-2.04) | 1.28 (0.89-1.69) | 1.45 (1.23-1.68) | 1.14 (0.81-1.48) | 1.31 (1.1-1.52) | 25.55 (18-33.98) | 1.11 (0.96-1.27) |
| Morocco | 1.71 (1-2.69) | 2.88 (2.7-3.06) | 0.9 (0.55-1.42) | 2.28 (2.13-2.43) | 0.82 (0.51-1.29) | 2.01 (1.87-2.15) | 20.47 (12.32-32.26) | 1.96 (1.84-2.08) |
| Mozambique | 1.81 (0.92-2.91) | 1.76 (1.67-1.84) | 1.25 (0.66-1.98) | 1.65 (1.56-1.75) | 1.28 (0.68-2.03) | 1.59 (1.49-1.68) | 31.53 (16.22-50.41) | 1.64 (1.54-1.74) |
| Myanmar | 0.68 (0.42-1.3) | 2.56 (2.42-2.7) | 0.39 (0.24-0.75) | 1.99 (1.9-2.08) | 0.37 (0.23-0.72) | 1.8 (1.73-1.87) | 9.33 (5.7-18) | 1.69 (1.62-1.76) |
| Namibia | 2.37 (1.62-3.34) | 1.98 (1.89-2.06) | 1.43 (0.99-1.98) | 1.58 (1.45-1.7) | 1.38 (0.96-1.9) | 1.44 (1.31-1.57) | 34.25 (23.08-48.48) | 1.37 (1.22-1.53) |
| Nepal | 1.33 (0.66-2.22) | 2.42 (2.14-2.7) | 0.81 (0.43-1.36) | 1.94 (1.67-2.21) | 0.8 (0.41-1.33) | 1.77 (1.5-2.03) | 18.77 (9.54-31.49) | 1.66 (1.39-1.93) |
| Netherlands | 14.32 (12.4-16.57) | 2.49 (1.94-3.05) | 4.45 (3.93-4.94) | 0.55 (0.34-0.76) | 3.15 (2.78-3.43) | -0.62 (-0.73--0.51) | 64.47 (58.18-69.49) | -0.79 (-0.9--0.68) |
| New Zealand | 25.35 (21.98-29.15) | 1.37 (1.12-1.62) | 6 (5.19-6.74) | 0.64 (0.45-0.82) | 3.15 (2.73-3.47) | 0.09 (-0.07-0.25) | 67.57 (60.03-73.84) | -0.11 (-0.26-0.03) |
| Nicaragua | 1.21 (0.82-1.6) | 2.38 (2.23-2.53) | 0.53 (0.38-0.7) | 1.55 (1.41-1.7) | 0.44 (0.31-0.58) | 1.14 (0.98-1.29) | 11.64 (8.14-15.13) | 1.17 (1.03-1.32) |
| Niger | 0.08 (0.03-0.16) | 3.32 (3.16-3.48) | 0.06 (0.02-0.11) | 3.14 (2.98-3.3) | 0.06 (0.02-0.11) | 3.12 (2.96-3.28) | 1.37 (0.41-2.63) | 2.96 (2.81-3.11) |
| Nigeria | 1.2 (0.42-1.92) | 2.86 (2.63-3.1) | 0.77 (0.28-1.2) | 2.44 (2.25-2.63) | 0.78 (0.3-1.19) | 2.29 (2.11-2.46) | 17.84 (6.39-27.9) | 2.35 (2.16-2.53) |
| Northern Mariana Islands | 2.68 (1.99-3.88) | -1.12 (-1.4--0.83) | 1.29 (1-1.86) | -1.35 (-1.6--1.09) | 1.13 (0.87-1.64) | -1.45 (-1.69--1.21) | 27.14 (20.94-38.89) | -1.32 (-1.58--1.06) |
| Norway | 19.83 (17.25-22.37) | 1.41 (0.96-1.86) | 5.55 (4.91-6.1) | 0.45 (0.23-0.67) | 3.42 (3.04-3.69) | -0.25 (-0.33--0.17) | 68.12 (62.22-72.59) | -0.53 (-0.66--0.41) |
| Oman | 3.45 (2.25-4.76) | 2.81 (2.48-3.14) | 1.47 (0.99-2.02) | 1.78 (1.45-2.12) | 1.19 (0.8-1.61) | 1.3 (0.96-1.65) | 27.97 (18.69-39.14) | 1.13 (0.76-1.49) |
| Pakistan | 2.64 (1.84-3.81) | 1.72 (1.61-1.82) | 1.71 (1.2-2.46) | 1.44 (1.3-1.58) | 1.72 (1.21-2.5) | 1.35 (1.19-1.5) | 40.86 (28.8-58.75) | 1.32 (1.16-1.48) |
| Palestine | 3.73 (2.19-4.94) | 1.73 (1.65-1.82) | 1.77 (1.06-2.33) | 0.96 (0.91-1.01) | 1.54 (0.92-2.02) | 0.61 (0.53-0.68) | 37.18 (21.86-49.08) | 0.59 (0.53-0.64) |
| Panama | 6.33 (4.89-7.96) | 1.64 (1.46-1.82) | 2.48 (1.98-3.05) | 0.85 (0.68-1.02) | 1.96 (1.53-2.38) | 0.46 (0.29-0.63) | 49.7 (39.12-60.68) | 0.53 (0.37-0.7) |
| Papua New Guinea | 0.36 (0.14-0.64) | 0.59 (0.46-0.73) | 0.22 (0.09-0.41) | 0.45 (0.34-0.56) | 0.22 (0.09-0.42) | 0.43 (0.33-0.52) | 5.54 (2.18-10.22) | 0.46 (0.36-0.56) |
| Paraguay | 2.96 (1.94-4.23) | 2.25 (2.13-2.37) | 1.38 (0.91-2) | 1.77 (1.66-1.89) | 1.2 (0.79-1.73) | 1.52 (1.41-1.64) | 29.06 (19.31-41.36) | 1.47 (1.37-1.57) |
| Peru | 4.46 (3.1-6.55) | 2.42 (2.12-2.73) | 1.81 (1.26-2.58) | 1.07 (0.78-1.36) | 1.46 (1.02-2.09) | 0.41 (0.14-0.69) | 35.86 (24.96-51.6) | 0.41 (0.14-0.69) |
| Philippines | 0.85 (0.62-1.14) | 1.8 (1.71-1.89) | 0.46 (0.34-0.63) | 1.67 (1.61-1.72) | 0.42 (0.32-0.58) | 1.58 (1.53-1.63) | 11.07 (8.24-14.66) | 1.59 (1.56-1.63) |
| Plurinational State of Bolivia | 3.19 (2.15-4.78) | 1.55 (1.46-1.63) | 1.79 (1.23-2.67) | 0.97 (0.88-1.05) | 1.69 (1.16-2.53) | 0.73 (0.65-0.81) | 40.73 (27.94-61.06) | 0.62 (0.53-0.71) |
| Poland | 4.7 (4.17-5.27) | 2.02 (1.46-2.57) | 2.6 (2.34-2.83) | 1.62 (1.21-2.03) | 2.49 (2.25-2.72) | 1.53 (1.17-1.9) | 55.05 (50.32-59.92) | 1.07 (0.71-1.42) |
| Portugal | 4.91 (4.3-5.61) | 0.95 (0.56-1.34) | 2.55 (2.26-2.83) | 0.44 (0.22-0.65) | 2.45 (2.15-2.71) | 0.28 (0.1-0.46) | 50.81 (45.82-55.92) | -0.04 (-0.22-0.15) |
| Principality of Monaco | 23.46 (11.54-39.15) | 1.86 (1.76-1.97) | 6.86 (3.49-10.95) | 1.22 (1.17-1.27) | 4.4 (2.29-6.84) | 0.76 (0.74-0.79) | 93.34 (47.42-150.07) | 0.61 (0.59-0.63) |
| Puerto Rico | 19.04 (15.04-23.7) | 1.89 (1.61-2.17) | 4.69 (3.83-5.65) | 0.62 (0.42-0.83) | 2.54 (2.12-2.97) | -0.35 (-0.52--0.17) | 62.31 (51.81-73.67) | -0.27 (-0.43--0.1) |
| Qatar | 4.04 (2.42-6.58) | 2.27 (1.98-2.55) | 1.48 (0.93-2.38) | 0.51 (0.16-0.86) | 1.12 (0.7-1.78) | -0.34 (-0.75-0.07) | 25.32 (15.71-40.27) | -0.27 (-0.62-0.09) |
| Republic of Cabo Verde | 0.26 (0.09-0.44) | 4.01 (3.94-4.08) | 0.14 (0.05-0.23) | 3.45 (3.35-3.55) | 0.13 (0.05-0.22) | 3.19 (3.07-3.31) | 3.02 (1.11-5.08) | 3.11 (3-3.22) |
| Republic of C么te d'Ivoire | 1.92 (1.22-2.74) | 1.3 (1.2-1.39) | 1.24 (0.82-1.77) | 0.96 (0.91-1.02) | 1.25 (0.84-1.77) | 0.87 (0.82-0.92) | 29.53 (19.16-42.05) | 0.83 (0.76-0.9) |
| Republic of Korea | 5.75 (3.26-7.79) | 4.44 (3.99-4.89) | 1.76 (1.01-2.32) | 2.14 (1.88-2.4) | 1.16 (0.66-1.53) | 0.83 (0.67-1) | 24.74 (14.64-32.59) | 0.55 (0.35-0.75) |
| Republic of Moldova | 3.09 (2.59-3.63) | 3.02 (2.51-3.54) | 1.13 (0.99-1.3) | 2.06 (1.63-2.5) | 0.82 (0.74-0.92) | 1.53 (1.14-1.92) | 22.16 (19.81-24.85) | 1.52 (1.14-1.91) |
| Republic of Nauru | 0.96 (0.46-1.78) | 0.08 (-0.3-0.47) | 0.58 (0.28-1.05) | -0.26 (-0.56-0.05) | 0.56 (0.27-0.98) | -0.39 (-0.66--0.11) | 14.36 (6.47-26.7) | -0.23 (-0.48-0.02) |
| Republic of Niue | 1.12 (0.59-1.64) | 1.35 (1.3-1.4) | 0.59 (0.31-0.83) | 0.98 (0.92-1.03) | 0.53 (0.28-0.75) | 0.83 (0.76-0.9) | 13.25 (6.82-18.95) | 0.75 (0.69-0.82) |
| Republic of Palau | 0.11 (0.06-0.16) | 0.61 (0.57-0.65) | 0.05 (0.03-0.08) | 0.34 (0.29-0.4) | 0.05 (0.03-0.07) | 0.24 (0.18-0.3) | 1.21 (0.7-1.77) | 0.1 (0.04-0.16) |
| Republic of San Marino | 8.56 (4.91-13.75) | 0.64 (0.14-1.15) | 2.42 (1.47-3.74) | -0.07 (-0.51-0.37) | 1.53 (0.96-2.31) | -0.59 (-1--0.18) | 32.55 (19.89-49.54) | -0.51 (-0.91--0.11) |
| Republic of the Gambia | 0.19 (0.11-0.28) | 1.93 (1.78-2.08) | 0.12 (0.07-0.18) | 1.7 (1.56-1.85) | 0.12 (0.07-0.18) | 1.62 (1.48-1.77) | 2.93 (1.75-4.31) | 1.59 (1.42-1.76) |
| Romania | 2.99 (2.5-3.5) | 2.36 (2.23-2.5) | 1.48 (1.28-1.68) | 1.68 (1.54-1.82) | 1.32 (1.14-1.49) | 1.4 (1.25-1.54) | 32.45 (27.95-36.68) | 1.15 (1-1.29) |
| Russian Federation | 4.76 (4.31-5.19) | 2.59 (2.12-3.05) | 1.81 (1.66-1.96) | 1.75 (1.43-2.07) | 1.37 (1.26-1.48) | 1.34 (1.09-1.59) | 35.48 (32.66-38.34) | 0.94 (0.73-1.15) |
| Rwanda | 2.09 (1.19-3.06) | 0.31 (0.02-0.6) | 1.35 (0.79-1.98) | -0.34 (-0.61--0.07) | 1.36 (0.81-2.02) | -0.43 (-0.69--0.17) | 32.82 (18.91-49.15) | -0.66 (-0.94--0.38) |
| Saint Kitts and Nevis | 9.73 (7.66-12.13) | 1.82 (1.49-2.15) | 3.73 (3.02-4.51) | 0.71 (0.46-0.96) | 2.77 (2.27-3.27) | 0.1 (-0.12-0.33) | 63.04 (50.97-75.96) | -0.02 (-0.24-0.2) |
| Saint Lucia | 12.68 (10.08-15.73) | 1.32 (1.04-1.6) | 4.57 (3.66-5.56) | 0.34 (0.13-0.54) | 3.34 (2.76-3.97) | -0.2 (-0.38--0.01) | 80.73 (66.34-97.66) | -0.05 (-0.23-0.13) |
| Saint Vincent and the Grenadines | 5.98 (4.99-7.16) | 1.52 (1.33-1.72) | 2.31 (2.01-2.67) | 0.92 (0.73-1.1) | 1.76 (1.55-1.98) | 0.6 (0.42-0.78) | 44.04 (38.44-50.33) | 0.7 (0.55-0.85) |
| Samoa | 1.14 (0.76-1.88) | 0.79 (0.73-0.85) | 0.63 (0.43-1) | 0.44 (0.39-0.49) | 0.59 (0.39-0.92) | 0.3 (0.25-0.35) | 14.44 (9.71-22.98) | 0.34 (0.31-0.38) |
| Sao Tome and Principe | 0.18 (0.06-0.31) | 5 (4.82-5.18) | 0.11 (0.04-0.18) | 4.65 (4.46-4.85) | 0.11 (0.03-0.18) | 4.52 (4.32-4.73) | 2.53 (0.79-4.11) | 4.33 (4.13-4.53) |
| Saudi Arabia | 3 (1.93-4.17) | 3.61 (3.5-3.72) | 1.27 (0.79-1.71) | 2.17 (2.1-2.24) | 1.04 (0.65-1.39) | 1.49 (1.4-1.57) | 25.68 (16.38-34.33) | 1.63 (1.54-1.72) |
| Senegal | 0.16 (0.05-0.26) | 3.92 (3.74-4.11) | 0.1 (0.03-0.17) | 3.67 (3.49-3.85) | 0.1 (0.03-0.17) | 3.62 (3.44-3.8) | 2.4 (0.78-4.07) | 3.51 (3.32-3.69) |
| Serbia | 2.51 (1.75-3.33) | 1.66 (1.41-1.91) | 1.62 (1.15-2.14) | 0.9 (0.75-1.04) | 1.63 (1.15-2.14) | 0.58 (0.46-0.71) | 37.9 (26.42-49.88) | 0.62 (0.51-0.72) |
| Seychelles | 3.53 (2.61-4.73) | 1.61 (1.45-1.78) | 1.72 (1.29-2.26) | 1.11 (0.94-1.28) | 1.51 (1.13-1.98) | 0.89 (0.72-1.07) | 37.12 (27.71-48.52) | 0.78 (0.63-0.93) |
| Sierra Leone | 0.11 (0.03-0.19) | 3.68 (3.49-3.87) | 0.07 (0.02-0.12) | 3.45 (3.27-3.62) | 0.07 (0.02-0.13) | 3.39 (3.21-3.57) | 1.71 (0.51-3.01) | 3.36 (3.18-3.55) |
| Singapore | 3.39 (2.87-3.94) | 1.07 (0.83-1.31) | 1.07 (0.94-1.21) | -0.54 (-0.68--0.39) | 0.73 (0.64-0.81) | -1.44 (-1.55--1.33) | 15.13 (13.62-16.67) | -1.74 (-1.86--1.62) |
| Slovakia | 9.76 (6.68-13.88) | 1.91 (1.81-2.01) | 3.31 (2.27-4.46) | 1.11 (1.02-1.21) | 2.34 (1.57-3.12) | 0.64 (0.54-0.74) | 54.77 (37.35-74.69) | 0.5 (0.4-0.6) |
| Slovenia | 9.75 (7.99-11.53) | 2.19 (1.85-2.53) | 3.44 (2.88-4.04) | 1.05 (0.83-1.27) | 2.58 (2.22-2.99) | 0.45 (0.26-0.65) | 55.68 (47.22-64.92) | 0.11 (-0.08-0.31) |
| Socialist Republic of Viet Nam | 0.69 (0.47-0.9) | 3.93 (3.88-3.98) | 0.32 (0.21-0.42) | 3.04 (3-3.09) | 0.27 (0.18-0.36) | 2.6 (2.55-2.65) | 6.52 (4.42-8.85) | 2.59 (2.54-2.64) |
| Solomon Islands | 0.41 (0.19-0.74) | 0.93 (0.78-1.07) | 0.26 (0.12-0.46) | 0.62 (0.49-0.74) | 0.25 (0.12-0.46) | 0.52 (0.4-0.63) | 6.6 (3.1-11.82) | 0.66 (0.54-0.78) |
| Somalia | 0.74 (0.31-1.56) | -0.36 (-0.41--0.31) | 0.53 (0.23-1.12) | -0.45 (-0.5--0.41) | 0.55 (0.24-1.16) | -0.44 (-0.48--0.4) | 13.63 (5.69-29.17) | -0.54 (-0.59--0.5) |
| South Africa | 3.91 (2.52-4.86) | 1.87 (1.73-2.01) | 2.18 (1.41-2.67) | 1.56 (1.44-1.68) | 2.03 (1.32-2.47) | 1.38 (1.24-1.52) | 51.66 (33.59-63.96) | 1.34 (1.21-1.48) |
| South Sudan | 1.66 (0.86-2.62) | 0.15 (0.07-0.23) | 1.11 (0.59-1.74) | -0.05 (-0.12-0.01) | 1.13 (0.6-1.76) | -0.1 (-0.16--0.05) | 27.66 (14.22-43.92) | -0.13 (-0.2--0.06) |
| Spain | 15.13 (12.85-17.56) | 0.8 (0.58-1.01) | 3.87 (3.36-4.37) | 0.19 (0-0.38) | 2.21 (1.91-2.45) | -0.27 (-0.46--0.08) | 45.93 (40.9-50.17) | -0.64 (-0.83--0.45) |
| Sri Lanka | 2.83 (1.7-4.4) | 2.93 (2.76-3.11) | 1.19 (0.74-1.83) | 1.84 (1.66-2.01) | 0.97 (0.61-1.44) | 1.3 (1.12-1.48) | 23.71 (14.31-35.2) | 1.24 (1.06-1.42) |
| Sudan | 1.12 (0.67-1.83) | 1.78 (1.6-1.96) | 0.62 (0.37-1.01) | 1.21 (1.06-1.35) | 0.58 (0.35-0.97) | 0.98 (0.85-1.1) | 14.81 (8.7-24.38) | 0.95 (0.82-1.08) |
| Suriname | 4.97 (3.39-6.91) | 1.77 (1.59-1.95) | 2.17 (1.49-3.01) | 1.19 (1.01-1.36) | 1.77 (1.22-2.44) | 0.89 (0.73-1.06) | 44.81 (30.77-61.5) | 0.91 (0.75-1.08) |
| Sweden | 10.26 (8.63-12) | 0.59 (0.26-0.92) | 3.49 (3.04-4.01) | -0.17 (-0.34-0.01) | 2.64 (2.29-2.97) | -0.59 (-0.68--0.5) | 50.03 (44.23-56.25) | -0.82 (-0.96--0.67) |
| Switzerland | 15.54 (13.07-18.24) | 0.7 (0.14-1.26) | 4.11 (3.52-4.7) | 0.07 (-0.36-0.5) | 2.41 (2.1-2.71) | -0.41 (-0.75--0.07) | 48.75 (43.34-54.63) | -0.78 (-1.12--0.44) |
| Syrian Arab Republic | 1.21 (0.73-1.86) | 2.47 (2.25-2.69) | 0.53 (0.33-0.78) | 1.49 (1.31-1.67) | 0.45 (0.28-0.66) | 1.02 (0.85-1.19) | 11.08 (6.78-16.38) | 0.91 (0.77-1.06) |
| Taiwan (Province of China) | 6.55 (5.5-7.64) | 2.89 (2.56-3.21) | 1.98 (1.75-2.24) | 2.08 (1.76-2.41) | 1.29 (1.16-1.42) | 1.57 (1.23-1.9) | 32.8 (29.82-35.68) | 1.44 (1.14-1.73) |
| Tajikistan | 0.36 (0.24-0.53) | -0.57 (-0.87--0.27) | 0.22 (0.14-0.31) | -0.66 (-0.92--0.39) | 0.21 (0.14-0.3) | -0.7 (-0.96--0.45) | 6.11 (4.06-8.88) | -0.75 (-1.03--0.47) |
| Thailand | 1.77 (1.1-3.37) | 2.28 (2.08-2.48) | 0.7 (0.43-1.31) | 1.13 (0.9-1.35) | 0.55 (0.34-1.04) | 0.54 (0.31-0.78) | 14.06 (8.76-26.55) | 0.67 (0.43-0.9) |
| The former Yugoslav Republic of Macedonia | 1.91 (1.22-2.57) | 1.88 (1.69-2.07) | 1.06 (0.69-1.41) | 1.27 (1.09-1.46) | 1.01 (0.66-1.32) | 1.04 (0.85-1.22) | 22.23 (14.42-29.33) | 0.86 (0.7-1.02) |
| Timor-Leste | 0.44 (0.27-0.88) | 1.68 (1.47-1.88) | 0.26 (0.16-0.52) | 1.28 (1.09-1.46) | 0.26 (0.16-0.51) | 1.13 (0.95-1.3) | 6.39 (3.84-12.63) | 1.09 (0.89-1.29) |
| Togo | 0.13 (0.04-0.23) | 3.9 (3.78-4.02) | 0.09 (0.03-0.15) | 3.67 (3.53-3.82) | 0.09 (0.03-0.15) | 3.62 (3.47-3.78) | 2.1 (0.6-3.65) | 3.54 (3.38-3.69) |
| Tokelau | 0.83 (0.42-1.25) | 1.49 (1.45-1.54) | 0.44 (0.21-0.64) | 0.94 (0.91-0.97) | 0.4 (0.19-0.58) | 0.71 (0.67-0.74) | 10.14 (4.99-15.01) | 0.73 (0.7-0.77) |
| Tonga | 3.41 (2.28-4.92) | 0.88 (0.75-1.01) | 1.84 (1.27-2.57) | 0.66 (0.5-0.83) | 1.7 (1.17-2.37) | 0.57 (0.39-0.75) | 41.61 (28.27-59.2) | 0.54 (0.37-0.7) |
| Trinidad and Tobago | 11.05 (8.08-14.42) | 1.84 (1.69-1.99) | 3.85 (2.88-4.95) | 0.78 (0.65-0.91) | 2.73 (2.07-3.49) | 0.17 (0.04-0.3) | 68.79 (51.55-89.07) | 0.25 (0.12-0.38) |
| Tunisia | 3.87 (2.39-5.73) | 2.52 (2.48-2.56) | 1.66 (1.05-2.42) | 1.52 (1.46-1.58) | 1.39 (0.86-1.97) | 1.04 (0.96-1.12) | 32.34 (20.41-47.24) | 1.05 (0.98-1.13) |
| Turkey | 5.67 (3.88-7.8) | 2.48 (2.28-2.68) | 2.33 (1.69-3.21) | 1.09 (0.9-1.29) | 1.89 (1.39-2.62) | 0.43 (0.24-0.63) | 45.96 (33.7-62.25) | 0.3 (0.11-0.48) |
| Turkmenistan | 2.08 (1.53-2.83) | 6.58 (5.97-7.2) | 1.09 (0.82-1.48) | 6.12 (5.52-6.72) | 0.99 (0.74-1.34) | 5.92 (5.33-6.52) | 31.83 (23.83-43.57) | 6.04 (5.45-6.64) |
| Tuvalu | 0.62 (0.32-0.93) | 1.37 (1.25-1.49) | 0.36 (0.19-0.53) | 0.95 (0.84-1.07) | 0.34 (0.18-0.5) | 0.82 (0.71-0.93) | 8.7 (4.61-12.94) | 0.76 (0.66-0.87) |
| Uganda | 4.71 (3.22-7.2) | 1.93 (1.82-2.04) | 3.08 (2.16-4.57) | 1.56 (1.42-1.7) | 3.11 (2.17-4.65) | 1.49 (1.34-1.64) | 73.85 (50.54-114.45) | 1.39 (1.22-1.57) |
| Ukraine | 3.03 (2.22-4.04) | 1.74 (1.53-1.95) | 1.19 (0.88-1.55) | 1.6 (1.36-1.84) | 0.92 (0.66-1.19) | 1.55 (1.28-1.83) | 26.4 (18.91-34.28) | 1.41 (1.16-1.67) |
| United Arab Emirates | 5.61 (3.87-7.61) | 1.71 (1.34-2.08) | 2.94 (2.06-3.93) | 1.54 (1.11-1.97) | 2.69 (1.93-3.59) | 1.5 (1.04-1.96) | 57.68 (41.04-77.14) | 0.65 (0.28-1.03) |
| United Kingdom of Great Britain and Northern Ireland | 20.81 (19.47-21.86) | 1.67 (1.38-1.96) | 5.23 (4.82-5.48) | 0.71 (0.57-0.85) | 2.89 (2.64-3.02) | -0.01 (-0.11-0.09) | 59.77 (56.16-62.37) | -0.28 (-0.38--0.19) |
| United Republic of Tanzania | 2.11 (1.2-3.13) | 0.88 (0.74-1.01) | 1.35 (0.78-1.98) | 0.59 (0.49-0.69) | 1.35 (0.79-1.99) | 0.52 (0.42-0.62) | 32.79 (18.66-48.95) | 0.47 (0.37-0.56) |
| United States of America | 5.32 (4.97-5.57) | 0.12 (-0.16-0.4) | 2.95 (2.7-3.09) | -0.46 (-0.61--0.32) | 2.84 (2.58-2.99) | -0.68 (-0.8--0.57) | 57.79 (54.27-60.26) | -1.05 (-1.17--0.93) |
| United States Virgin Islands | 10.19 (6.15-16.31) | 0.76 (0.48-1.04) | 3.29 (2.03-5.18) | 0.09 (-0.17-0.34) | 2.2 (1.34-3.5) | -0.37 (-0.62--0.12) | 55.06 (33.6-87.99) | -0.19 (-0.41-0.04) |
| Uruguay | 8.52 (7.41-9.64) | 1.3 (1.17-1.44) | 3.58 (3.24-3.97) | 0.57 (0.46-0.68) | 2.94 (2.67-3.21) | 0.22 (0.12-0.32) | 67.11 (61.84-72.99) | 0.09 (-0.01-0.18) |
| Uzbekistan | 0.38 (0.29-0.51) | 1.66 (1.2-2.12) | 0.21 (0.16-0.26) | 1.35 (0.96-1.74) | 0.19 (0.15-0.24) | 1.18 (0.81-1.55) | 5.81 (4.4-7.42) | 1.27 (0.91-1.64) |
| Vanuatu | 0.47 (0.23-0.76) | 0.7 (0.58-0.81) | 0.3 (0.14-0.49) | 0.51 (0.42-0.6) | 0.3 (0.14-0.48) | 0.46 (0.38-0.53) | 7.55 (3.66-12.06) | 0.55 (0.45-0.65) |
| Yemen | 0.83 (0.44-1.48) | 1.48 (1.31-1.66) | 0.49 (0.27-0.88) | 1.09 (0.94-1.23) | 0.48 (0.26-0.86) | 0.94 (0.8-1.07) | 11.96 (6.41-21.14) | 0.85 (0.71-0.99) |
| Zambia | 3.69 (1.57-6.54) | 2.87 (2.42-3.32) | 2.34 (1.03-4) | 2.34 (1.99-2.69) | 2.31 (1.04-3.88) | 2.18 (1.85-2.5) | 59.44 (24.57-104.61) | 2.32 (1.97-2.67) |
| Zimbabwe | 5.29 (2.87-7.6) | 1.39 (1-1.79) | 3.51 (1.94-5.05) | 1.4 (0.97-1.82) | 3.53 (1.96-4.97) | 1.41 (1-1.82) | 88.68 (48.75-126.74) | 1.62 (1.16-2.07) |

***ASPR*** age-standardized incidence rate, ***ASIR*** age-standardized incidence rate, ***ASMR*** age-standardized mortality rate, ***ASDR*** age-standardized disability-adjusted life-year rate, ***EAPC*** estimated annual percentage change, ***UI*** uncertainty interval, ***CI*** confidence interval


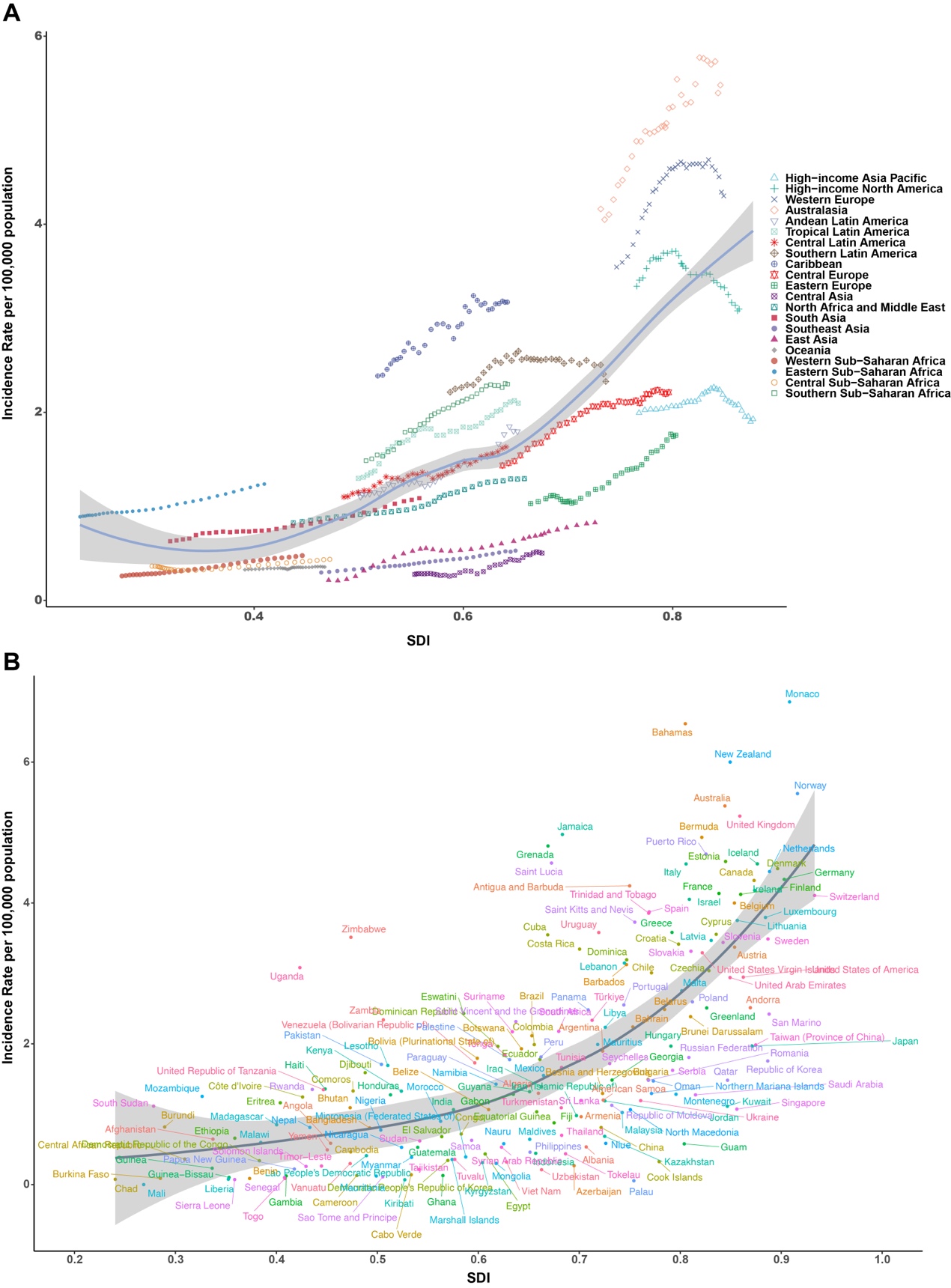


**FigureS1**. MM ASIR (based on SDI) for 21 regions and 204 countries and regions. **A** MM ASIR for 21 regions from 1990 to 2021 based on SDI. **B** MM ASIR for 204 countries and regions based on SDI (2021). ***ASIR*** age-standardized incidence rate, ***MM*** multiple myeloma, ***SDI*** sociodemographic index


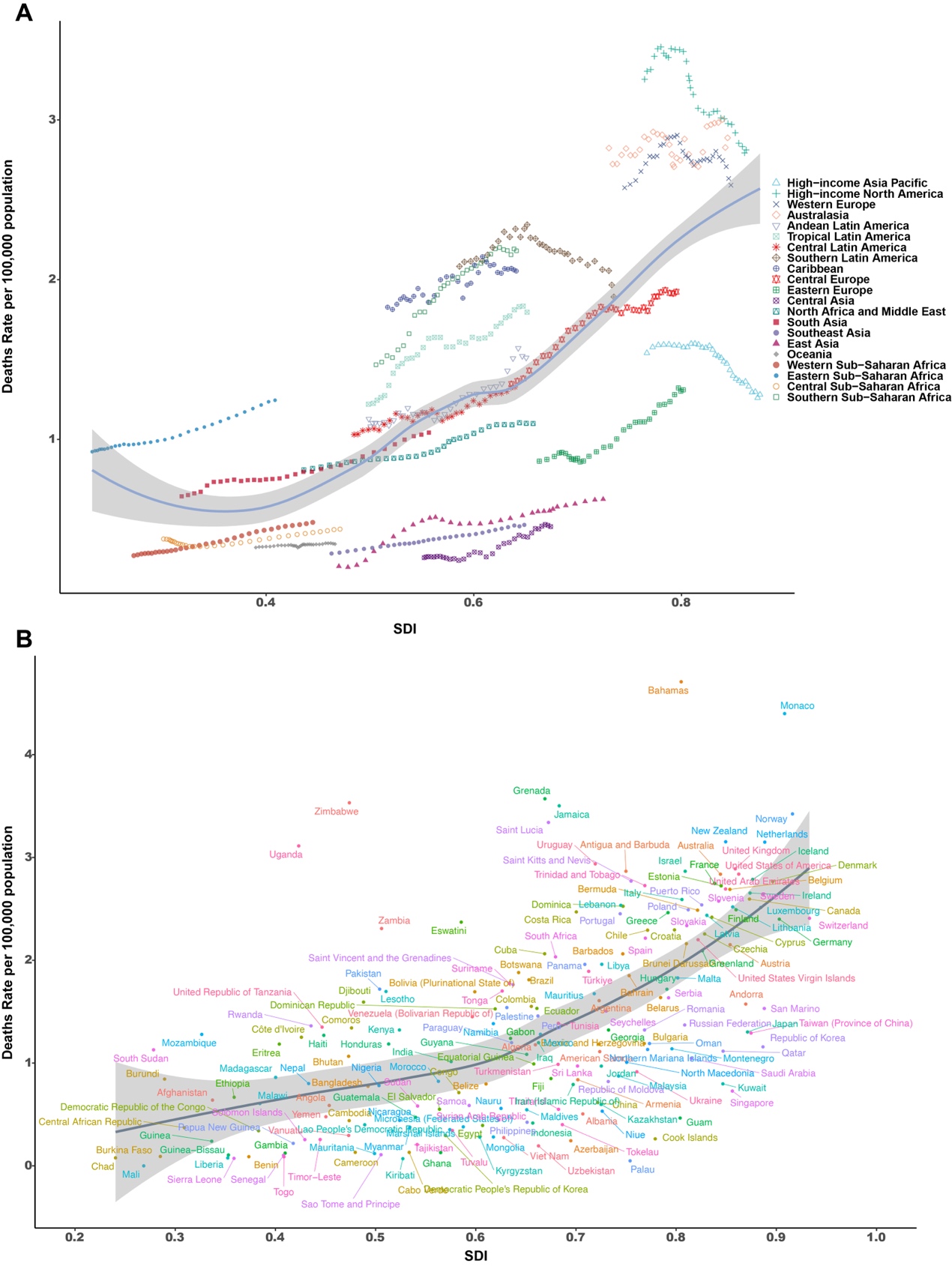


FigureS2. MM ASMR (based on SDI) for 21 regions and 204 countries and regions. **A** MM ASMR for 21 regions from 1990 to 2021 based on SDI. **B** MM ASMR for 204 countries and regions based on SDI (2021). ***ASMR*** age-standardized mortality rate, ***MM*** multiple myeloma, ***SDI*** sociodemographic index


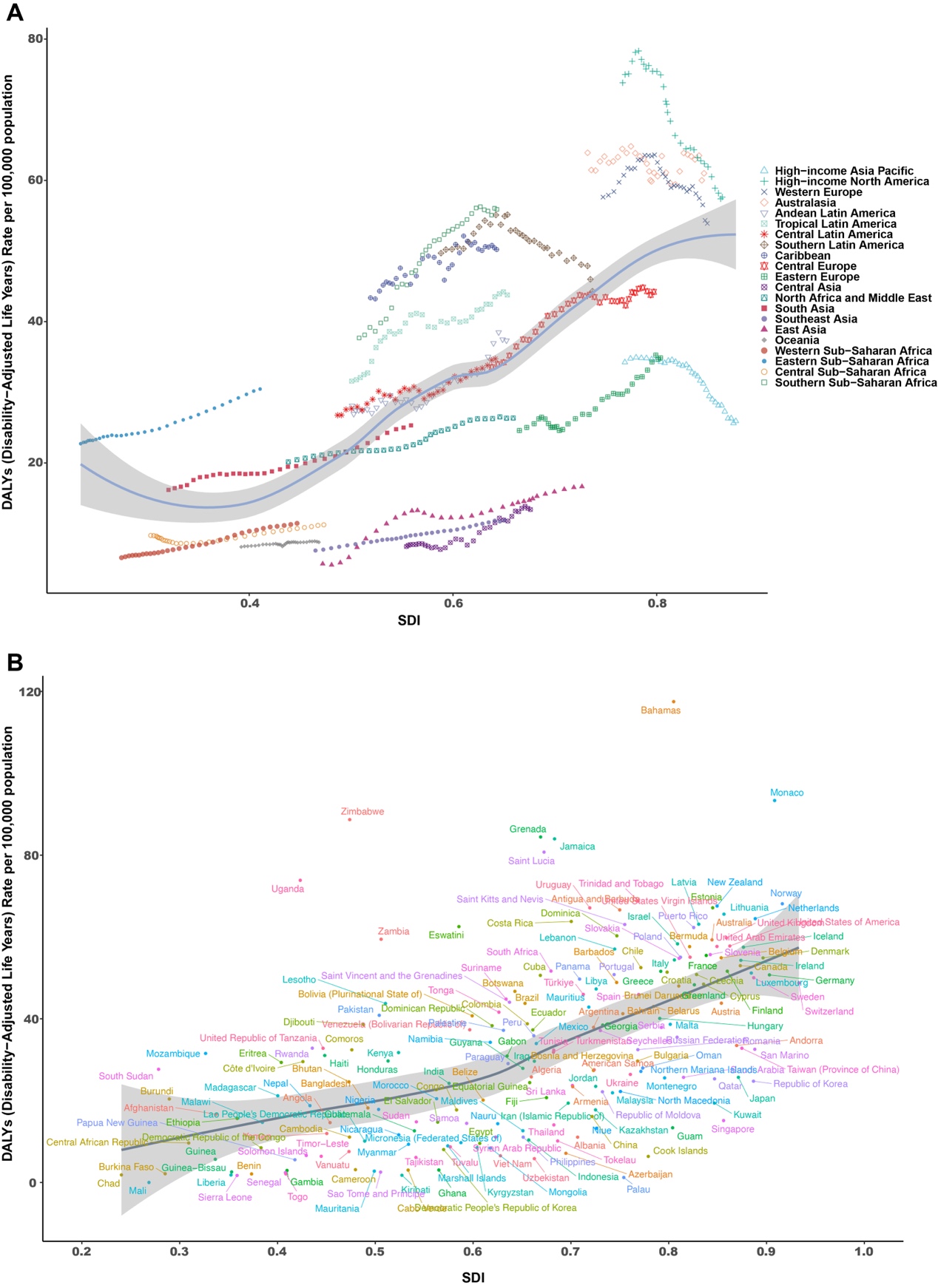


FigureS3. MM ASDR (based on SDI) for 21 regions and 204 countries and regions. **A** MM ASDR for 21 regions from 1990 to 2021 based on SDI. **B** MM ASDR for 204 countries and regions based on SDI (2021). ***ASDR*** age-standardized DALYs rate, ***MM*** multiple myeloma, ***SDI*** sociodemographic index


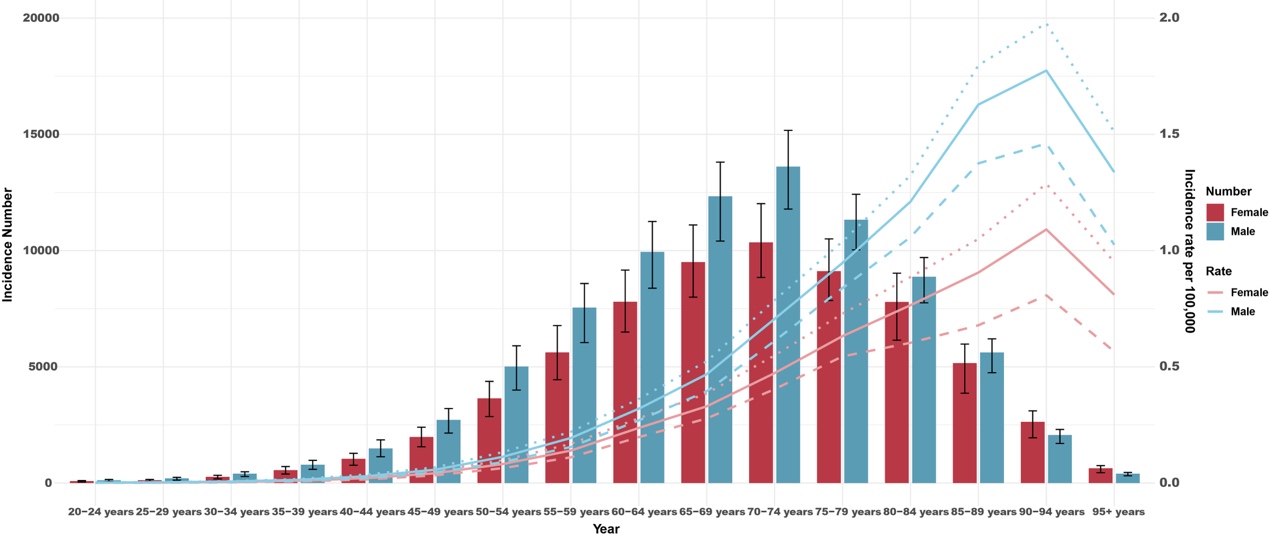


FigureS4. Global number of incidence cases and incidence estimates of multiple myeloma per 100,000 population by age and sex, 2021; Dotted and dashed lines indicate 95% upper and lower uncertainty intervals, respectively.


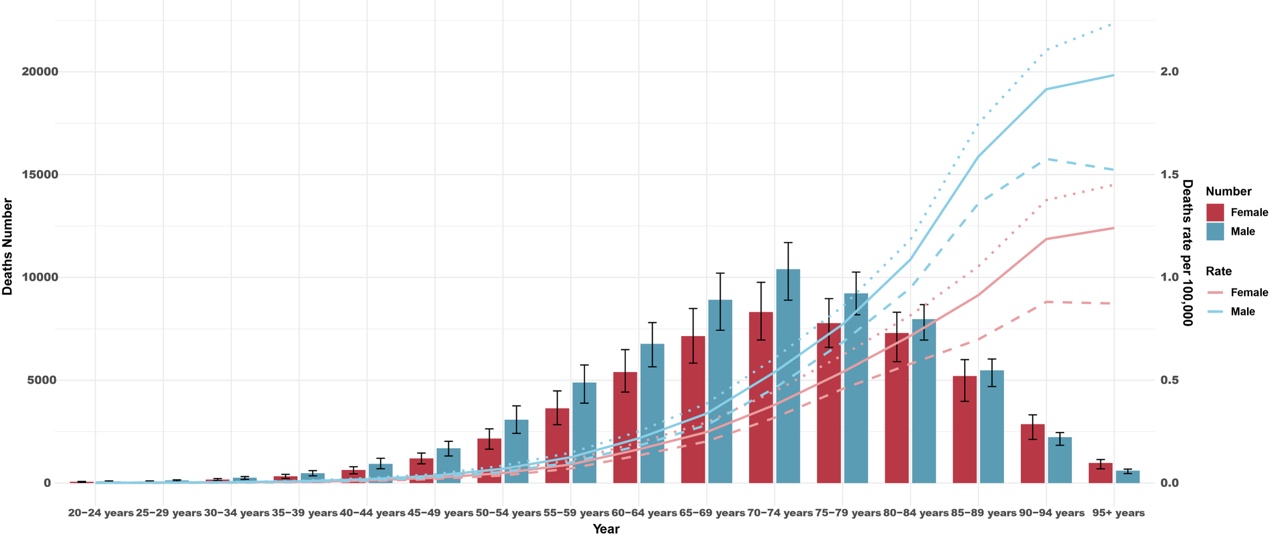


FigureS5. Global number of death cases and death estimates of multiple myeloma per 100,000 population by age and sex, 2021; Dotted and dashed lines indicate 95% upper and lower uncertainty intervals, respectively.


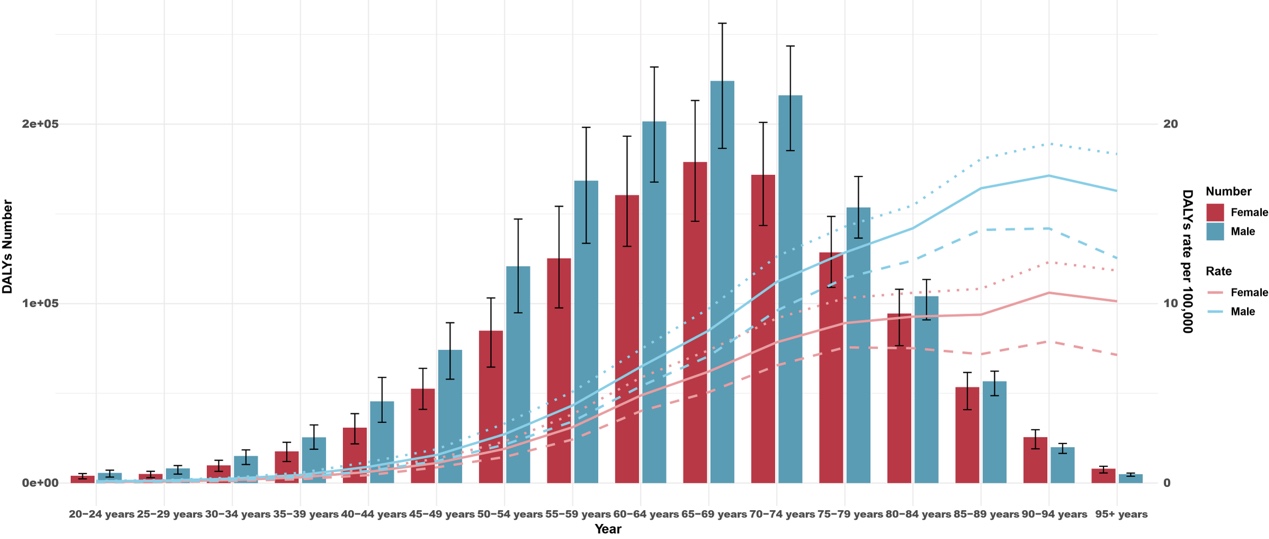


FigureS6. Global number of DALYs cases and DALYs estimates of multiple myeloma per 100,000 population by age and sex, 2021; Dotted and dashed lines indicate 95% upper and lower uncertainty intervals, respectively.


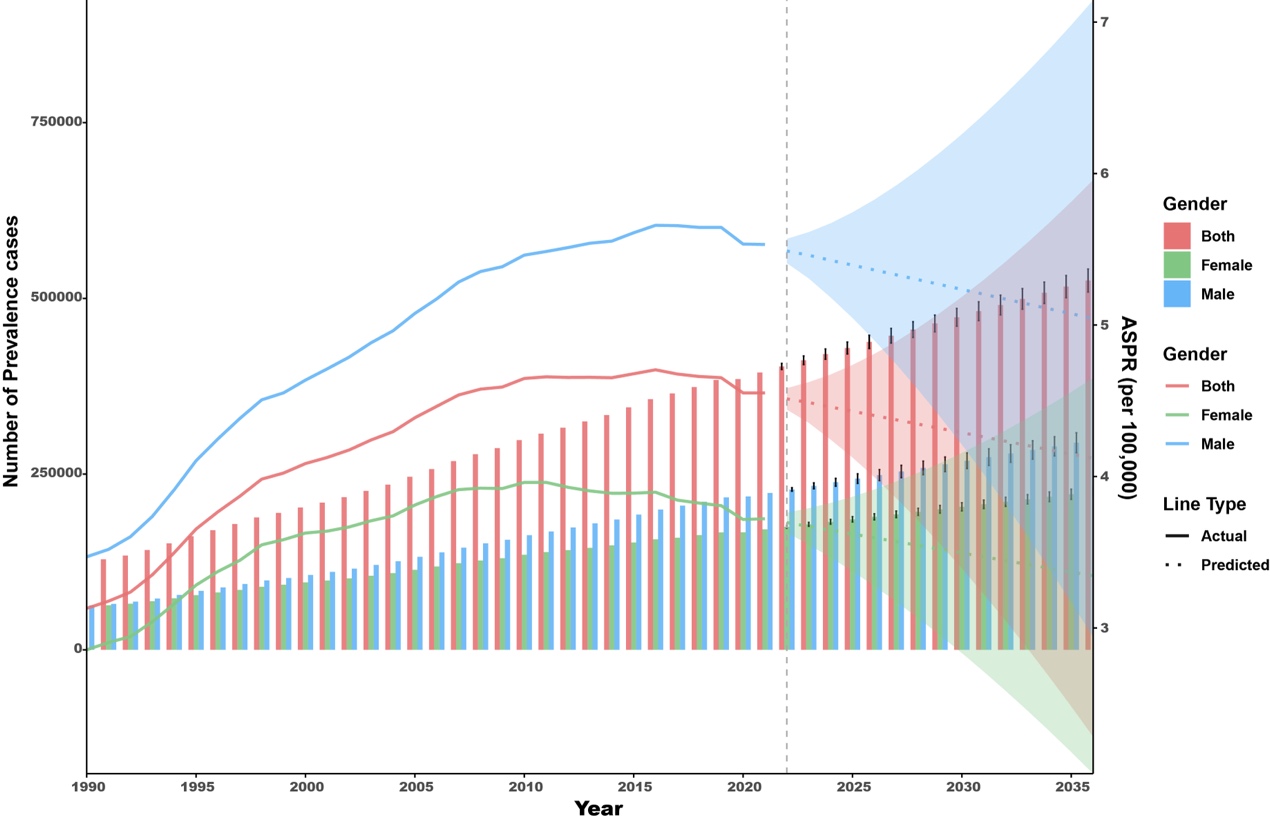


FigureS7. Forecasting the Global Prevalence and ASPR of Multiple Myeloma: A 15-Year Projection Using the ARIMA Model. The dashed line represents the predicted year, and indicates 95% upper and lower uncertainty intervals, respectively.


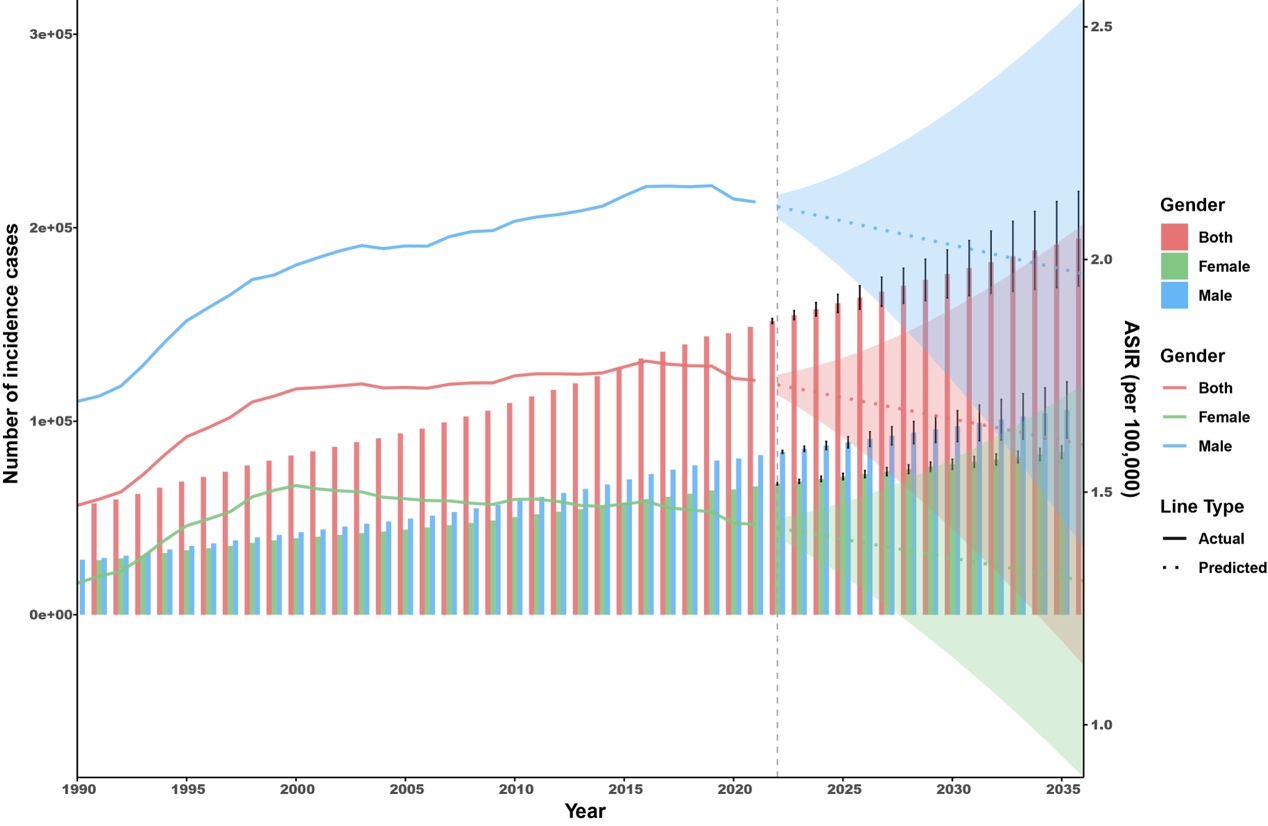


FigureS8. Forecasting the Global Incidence and ASIR of Multiple Myeloma: A 15-Year Projection Using the ARIMA Model. The dashed line represents the predicted year, and indicates 95% upper and lower uncertainty intervals, respectively.


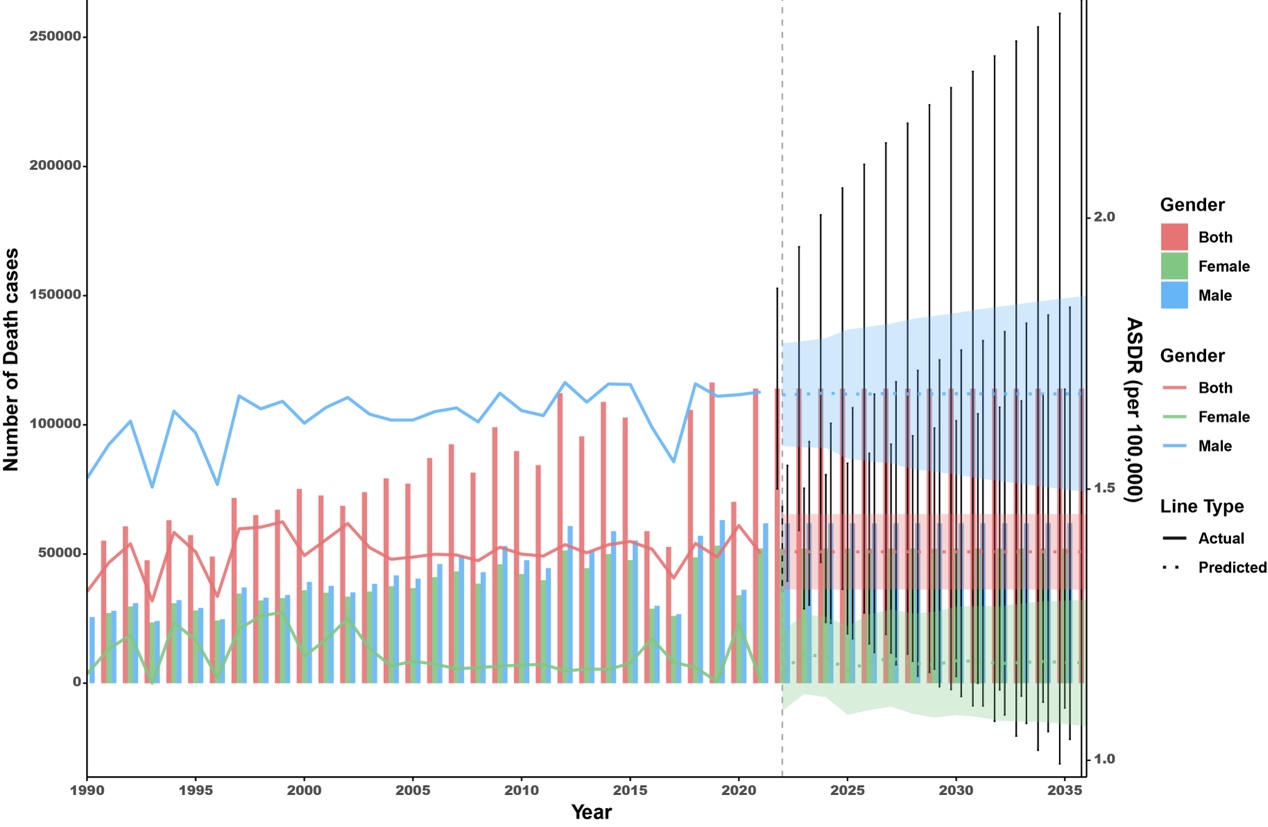


FigureS9. Forecasting the Global Mortality and ASMR of Multiple Myeloma: A 15-Year Projection Using the ARIMA Model. The dashed line represents the predicted year, and indicates 95% upper and lower uncertainty intervals, respectively.


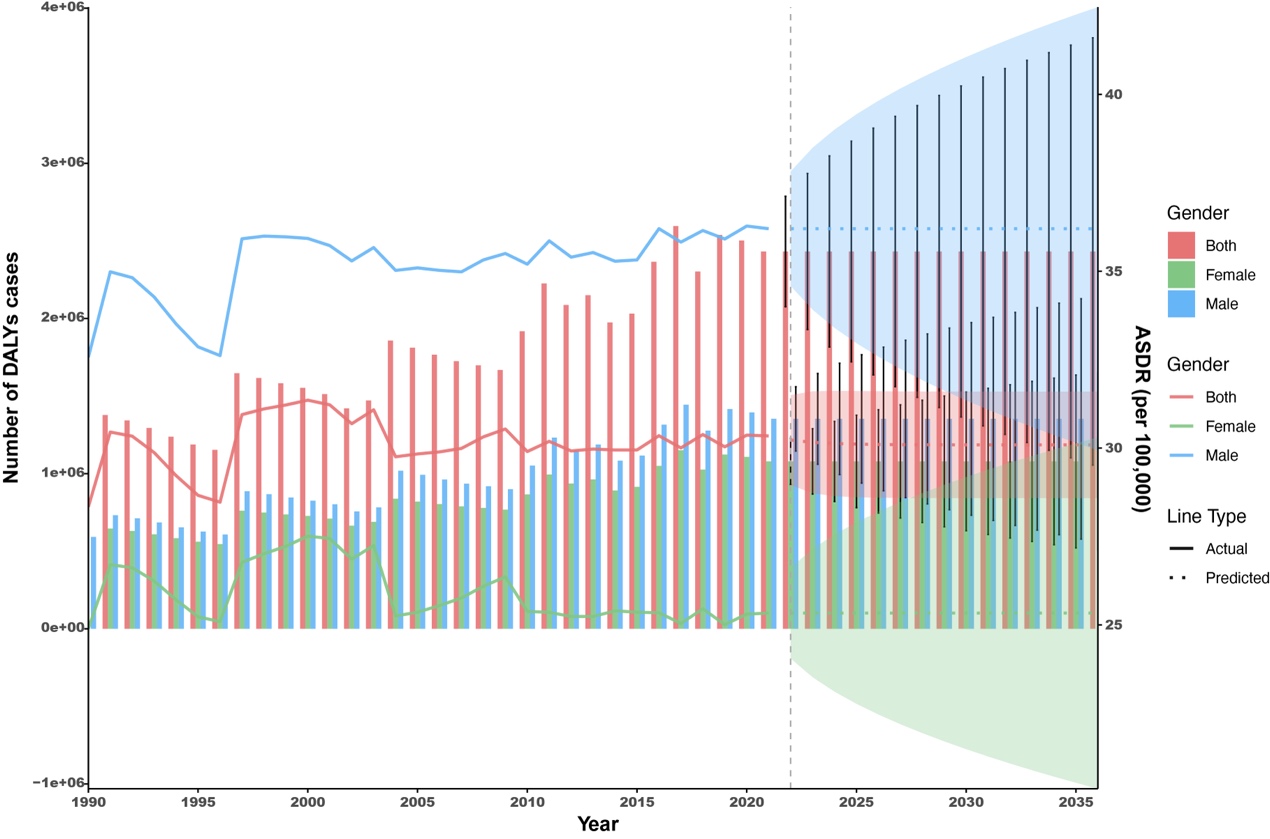


FigureS10. Forecasting the Global DALYs and ASDR of Multiple Myeloma: A 15-Year Projection Using the ARIMA Model. The dashed line represents the predicted year, and indicates 95% upper and lower uncertainty intervals, respectively.
